# Targeting Microorganisms in Lignocellulosic Biomass to Produce Biogas and Ensure Sanitation and Hygiene

**DOI:** 10.3390/microorganisms14020299

**Published:** 2026-01-27

**Authors:** Christy Echakachi Manyi-Loh, Stephen Loh Tangwe, Ryk Lues

**Affiliations:** 1 Centre of Applied Food Sustainability and Biotechnology, Central University of Technology, Bloemfontein 9301, South Africa; 2 Estate and Infrastructure, Central University of Technology, Bloemfontein 9301, South Africa

**Keywords:** biomass, microorganisms, anaerobic digestion, bacterial inactivation, biogas, sanitation, hygiene

## Abstract

Microbial components are part of the composition of all waste, including lignocellulosic biomass (e.g., agricultural, domestic, industrial, and municipal wastes) generated via human activities. If little attention is given to these wastes or if they are not adequately managed, they tend to end up in the environment (soil, water, and farmland), decomposing naturally through microbial activities, producing greenhouse gases, causing eutrophication, preventing sunlight penetration, and depleting oxygen in the water. Several treatment methods are applicable to these wastes. However, anaerobic digestion is presented as the best option to properly treat the waste. It is regarded as the best technique to achieve sustainable energy development in both developing and developed countries. During anaerobic digestion, the organic matter in the waste is converted via the concerted activities of microbes belonging to different trophic levels, in the absence of oxygen, to yield biogas (renewable energy), bio-fertiliser, and sanitisation of the waste, rendering it better and safer for human handling. Varying levels of loss of bacterial viability and their antibiotic-resistance genes are observed with this process, as bacteria differ in susceptibility to temperature, pH, nutrient scarcity, and the presence of antimicrobials. Anaerobic digestion of agricultural residues and the immediate processing (post-treatment) of the digestate help to stabilise the digestate, making it safe for land applications, tackling waste management, and protecting food chains from contamination, in addition to the environment. This review focuses on the anaerobic digestion of lignocellulosic biomass, yielding biogas as energy, alongside sanitising the wastes by inactivating microbial components found therein, therefore reducing the contamination potential of the effluent or digestate discharged from the biodigester following the process. Several findings registered by different researchers through different studies performed in different countries under different scenarios while employing varying methods have been assembled in a chronological fashion to emphasise similarities and divergences or variations that deepen knowledge pertaining to the significance of the anaerobic digestion process in terms of the microbial interactions responsible for producing energy, addressing sanitisation and hygiene crisis, and the post-treatment of the digestate to ensure its use as biofertiliser. In other words, it is a comprehensive review that synthesises knowledge from multiple fields covering comparative aspects of anaerobic digestion in terms of sanitation, hygiene, and energy production and consolidates it in a single document to present and address the problem of waste management through anaerobic digestion technology.

## 1. Introduction

Owing to rapid industrialisation and urbanisation, human activities produce huge quantities of different waste, including lignocellulosic waste, and they may include agricultural, forestry, or wood waste [1]. Agriculture is at the centre of policy agendas across the world due to its importance and relevance. This sector must provide adequate food of high quality for a population that is on a continuous rise. In most low- and middle-income countries, including South Africa, agriculture generates huge volumes of biomass residues, for example, lignocellulosic biomass. Lignocellulosic biomasses are readily available, consisting of agricultural residues/wastes (e.g., corn straw, corn cobs, other crop residues, and animal manure). They are characterised based on their biodegradability, renewability, biocompatibility, and abundance, making them highly valuable [2]. The methods employed to conventionally manage these wastes relate to huge environmental and public health hazards, including air, water, and soil pollution [3]. Moreover, these disposal options carry considerable costs and logistical limitations [4]. Traditionally, the generated animal manure is applied on land as an option to attain both management of animal manure and enhancement of agricultural soil quality [5], serving as an organic fertiliser for crop and pasture growth. The existing management practices involving manure vary widely on a global scale. However, animal manure may contain varying degrees of microbial and chemical contaminants [6], being influenced by livestock practices, which tend to vary from one individual to the next. More elaborately, several contaminants, including antibiotics, heavy metals, hormones, antibiotic-resistance genes, and pathogens found in the manure, may be released into the environment. This attracts great attention because substantial levels of pathogens have been detected in the soil and agricultural products following long-term manure application. In addition, the land application of manure leads to considerable pollution of the soil, water, and greenhouse gas emissions due to high levels of organics and nutrients [7]. Nonetheless, when manure is appropriately handled and circulated, it can be applied to fields as organic fertiliser, justifying the potential for contamination of soil and water [8]. An alternative to sustainable agriculture can be reached through fertilisation with organically derived fertiliser that provides adequate nutritious food for everybody while curtailing environmental risks [9]. Consequently, there has been an incessant pursuit for options to correctly handle the waste for the reason that water and environmental policies are regulated on a continuous basis to make sure the management of the ever-rising waste created by the growing activities in animal farming and agricultural activities is performed [10].

Animal manure consists of complex molecules in terms of nutrients and a variety of microorganisms endowed with the capacity to decompose organic matter [11]. In addition, livestock and other agricultural waste are materials of great interest owing to their relative abundance and availability worldwide [12]. The different lignocellulosic wastes are renewable feedstocks harbouring energy-stored compounds, serving as the principal substrates for biogas production, and assisting in effective waste management. Managing waste through anaerobic digestion (AD) technology explores the activities of microorganisms, which transform the organic matter into a main renewable bioenergy source. Microbial metabolic activities are instrumental for production, functionalisation, processing, etc. For the circular economy, microbial metabolism would be exploited for agri-food waste valorisation, and/or biofuel production [13]. The complex community of microorganisms that convert wastes into biogas through anaerobic digestion is collectively termed “microbiota”, performing a series of biological reactions [14]. The microbiota involves a great diversity and relies on several factors, including feedstock type, seed inoculum, temperature, granulation, aeration, mixing speed, pre-treatment type, digester design, organic loading rate (OLR), solids retention time (SRT), and hydraulic retention time (HRT) [15]. Owing to the complex and heterogenous ecosystem and the massive diversity of uncharacterised microbes in anaerobic digesters, the word “black box” is usually used in describing these anaerobic digesters, and the AD microbiome is known as “black matter”. The term “black matter“ emphasises that, regardless of the wide applications, microbial communities engaged in the AD process are not fully understood [16].

Anaerobic digestion is a microbe-driven biochemical process. Therefore, the interactions between the microbial communities are the core of the process [17], indicating that the role of the microorganisms is undisputable. They work interactively in a consortium through four complicated but coordinated and interdependent biochemical reactions or stages, including hydrolysis, acidogenesis, acetogenesis, and methanogenesis, making use of their machinery to degrade organic matter in the waste to biogas; each is performed by different microbial groups [18]. Apparently, the stability of this process and the subsequent generation of the biogas yield are greatly dependent on the synergistic interactions added to the appropriate balance of substrate flow between the different microbial communities [19]. Syntrophy is the basic mechanism that sustains microbial communities in AD, as the methanogenic archaea feed on the by-products of bacteria [20]. Castro-Ramos et al. [21] described the complex syntrophic relationship as obligate mutualistic cooperation in which the microorganisms live, and so, variations expressed in one group can modify the chain of anaerobic sequences and, thus, the complete AD process [22]. The entire microbiota in an anaerobic digester system is essential for the various phases of the AD, along with stabilising the redox potential of the system [15]. Kabaivanova et al. [23] analysed the bacterial diversity in the thermophilic anaerobic degradation of straw and noted that more than one-third of bacteria belonged to class Clostridia (32.9%), followed by Bacteroidia (21.5%), Betaproteobacteria (11.2%), Gammaproteobacteria (6.1%), and Alphaproteobacteria (5%). The most prominent genera among them were *Proteiniphilum*, *Proteiniborus*, and *Pseudomonas*. A fraction of 1.37% represented archaeal microflora belonging to the genera that were most abundant in the thermophilic bioreactor, including *Methanocorpusculum*, *Methanobacterium*, *Methanomassiliicoccus*, *Methanoculleus*, and *Methanosarcina*.

Knowledge of the microbial population in each stage, alongside their metabolic properties and interactions, is crucial to improve the performance of AD [21]. The advent of novel molecular techniques, including whole genome sequencing, next-generation sequencing (NGS), and omics approaches (e.g., genomics, metagenomics, transcriptomics, proteomics, etc.), provides an in-depth understanding of the microbial communities involved in AD [15]. By so performing, revealing insights into the distinctive interactions occurring among the communities in terms of their structure, dynamics, and metabolic pathways (functions). As a result, they help to guide in identifying and controlling various factors pivotal in optimising the biogas process, enhancing its stability, and improving overall performance [14]. Biogas, a renewable energy source, is produced during the anaerobic degradation of these wastes by anaerobic microorganisms in an airtight chamber known as the biodigester [24]. However, owing to the occurrence of the three biopolymers in plant-based lignocellulosic biomass (cellulose, lignin, and hemicellulose), the substrate demonstrates recalcitrance to the anaerobic digestion process, hampering optimal biogas yield. Although this ability can be addressed via pre-treating the substrates, the process may generate inhibitory or toxic compounds that will affect the bacterial activity in the hydrolysis and fermentation processes during anaerobic digestion [25]. In other words, the agricultural residues, comprising mostly high lignocellulosic content, are usually present with a high carbon to nitrogen (C/N) ratio, making them comparatively poor substrates for biogas production. Nonetheless, to achieve the optimum C/N ratio of 20–30:1 for biogas, they are often co-digested with other mixtures of waste, including animal manure. Table 1 shows the differential composition of agricultural residues and animal manures based on physicochemical and microbiological parameters.

Furthermore, animal manures have been reported to be suitable substrates for anaerobic digestion, capable of facilitating the degradation of the organic matter in the biomass to yield methane (biogas) and carbon dioxide and traces of hydrogen, nitrogen, and hydrogen sulphide, in addition to a nutrient-rich fertiliser [42]. The production of animal manure has been on a continuous rise as the global total demand for meat and its derivatives experiences a rise caused by increased affluence and improved income among the middle-class population [43]. The high demand for livestock products results in a corresponding response by farmers to involve the routine application of antibiotics in intensive animal farming to ensure sustainable production [44]. Anaerobic co-digestion of animal manure and agricultural plant-based waste will assist in the efficient management of the waste, reducing their quantities as well as producing biogas, which can serve as energy for cooking in rural areas of the country that are not yet connected to the national grid. This process will aid in sanitising these wastes (deactivating bacterial pathogens and antibiotic-resistance genes, ARGs), since they are treated in a confinement, preventing odour and greenhouse gas release from animal wastes into the environment, which might lead to climate change/global warming, and in turn affect public health.

The AD process can cause a reduction in bacterial and antibiotic resistance concentrations through several mechanisms, including temperature, pH, enzymatic degradation, and microbial competition for nutrients. Possibly, several factors, including the substrate being treated, the specific pathogen present and its level, and the operating process condition, can vary the effectiveness of pathogen reduction concentration during AD [45].

Temperature: In the main, the temperature of AD impacts both the growth and the persistence of microorganisms in the system, since different microbial groups exhibit variable temperature tolerance. Thus, process temperature is a salient factor that enforces a strong selective pressure on the level and composition of microbial communities within the digester [46]. The temperature at which the AD process is being operated plays a role in pathogen inactivation; thus, it is a very important factor in pathogen inactivation during AD. The temperature at which the system is operated can be described as psychrophilic, mesophilic, or thermophilic [47]. Thermal conditions applied to AD could provoke the destruction of pathogens via the break-up of the gel structure and cell lysis [48]. Generally, thermophilic conditions are more effective at inactivating pathogens because of the higher growth rates of microorganisms and an increase in metabolic activity. The higher temperatures enable the reactions to take place at a faster rate while causing a greater reduction in pathogen levels. Thus, thermophilic AD is favourable for treating waste with high pathogen levels [45]. In addition, anaerobic digestion performed at thermophilic temperatures resulted in the effective removal of antibiotic resistance genes harboured by microorganisms. Budatalla et al. [49] registered through quantitative polymerase chain reaction (PCR) analysis and metagenomic sequencing that higher temperatures notably increased the reduction in ARGs, with the most considerable reductions observed at 65 °C. The authors further confirmed the reductions as temperature-dependent and correlated with alterations in the microbial community structure; for example, specific bacterial genera like *Alicycliphilus, Macellibacteroides*, *Dokdonella*, *Ahniella*, *Thauera*, and *Zoogloea* associated with ARGs showed decreased abundance at elevated temperatures. However, the major limitation faced by AD systems operating at high temperatures is the cost involved in supplying the energy for heating the system.

pH: Fundamentally, pH is a direct representation of hydrogen ion (H^+^) and hydroxyl ion (OH^−^) concentrations in a milieu [50], and it is crucial for microbial activity and pathogen inactivation. Notwithstanding, microorganisms can be categorised based on the optimum pH that favours their growth into acidophiles, neutrophiles, and alkaliphiles, yet the optimum pH for AD occurs in the range 6.8–7.4. Nonetheless, Akkermans et al. [51] reiterated that microorganisms tend to thrive and grow optimally at a pH of approximately 7. Hence, greater deviations from the neutral pH toward acidity or alkalinity can affect the survival of pathogens during the AD process, therefore facilitating the sanitisation of the waste material [47]. Castro-Ramos et al. [21] noted that a deviation from optimal pH conditions exerts huge effects on shaping the microbial communities that are responsible for performing essential processes, including AD. Zamri et al. [52] affirmed that solution pH is among the key factors influencing the performance of functional microbes in the process. Therefore, indicating that the microbes involved in this process will respond differently to pH changes as they belong to different physiological and nutritional groups. Clearly, pH affects microbial activity, yet metabolic processes/reactions also alter the pH [53]. In this light, Tang and colleagues [54] noted that variation in pH caused by the degradation of different metabolites at the different phases during the AD process can greatly contribute to bacterial inactivation. pH is affected by the level of volatile fatty acids (VFAs) and the buffering capacity of the system. Seemingly, Jiang et al. [55], in their study, co-digested food waste and pig manure and identified free VFAs as a significant factor in effectively reducing *E. coli* and total coliform counts to levels below the limit of detection (LOD, 10^2^ CFU/g) within 4–7 days. The alkalinity of the digesting medium offers buffering capacity, assisting in maintaining stable pH levels within the digester [45]. The concentration of VFAs can be determined by the initial total solid concentration, which in turn is strongly dependent on the ratio between inoculum and substrate [56]. Apparently, Otite and colleagues [47] noted that about 1.5 log CFU/mL of *Clostridium* was reduced by acidogenic conditions under an inoculum substrate ratio of 0.25 and 0.50. More elaborately, the AD process may be linked with *in situ* formation of alternating changes in acid/alkaline conditions, which encourages the generation of free VFAs and free ammonia (FA) at different stages of digestion. Thus, leading to an efficient and high bacterial inactivation. More especially, at the acidogenesis stage, during which volatile fatty acids are formed, the AD system will experience a drop in the pH level due to the accumulation of VFAs. Sun and co-authors [57] narrated that the accumulation of organic acids may originate from the fact that the high level of organic acids produced by the acidogens is not proportionally removed by the slow-growing methanogens that are meant to degrade the end products of the acidogenesis phase. Free ammonia produced from protein digestion demonstrates antimicrobial activity. At this point, VFAs and FA are considered as pathogen inhibitors [45]. Tang et al. [54] explained that the low pH induced VFAs during acidogenesis dominated *E. coli* inactivation, while the synergistic effects of OH^−^ and FA caused inactivation of *E. coli* cells during methanogenesis. Sun et al. [57] demonstrated a complete failure of the AD process under low pH conditions due to a decrease in the activity of methanogenic biomass.

Enzymatic degradation: The anaerobic digestion is performed by microorganisms alongside their enzymes, especially at the hydrolytic stage, where microbes secrete a host of hydrolytic enzymes (e.g., hydrolases, lipases, amylases, or cellulases), causing the breakdown or degradation of polymeric compounds into simpler, more biodegradable subunits [58]. The activity of these enzymes is often temperature-dependent and acts within specific temperature ranges. However, extremely high temperatures lead to denaturation of the enzymes that are necessary for the metabolic processes, resulting in the loss of their functions in sustaining the viability of microorganisms [59]. Consequently, the microorganisms may become inactivated or die off.

Microbial competition: The initial load and diversity of microorganisms vary across the different feedstocks or substrates [45]. Similarly, each substrate varies from another based on its nutrient makeup in terms of polymers (e.g., carbohydrates, lipids, and proteins). The presence of a varied microbial community can outcompete pathogens for resources and produce inhibitory substances, contributing to pathogen reduction [60]. In addition, indigenous, well-adapted microorganisms outcompete pathogenic or non-adapted bacteria and scavenge for limited resources, metabolise them, and produce inhibitory metabolites, shifting the pH, thereby creating a hostile environment. The competition between microorganisms can be pointed out in terms of the factors needed for their growth and survival, e.g., oxygen. Based on the requirement of oxygen for growth, it is obvious that strict anaerobes will outcompete facultative and microaerophilic organisms during this process, which tends to occur under anaerobic conditions [42]. Thus, the condition under which the process is operating (strictly anaerobic) is itself a strong selective pressure on the level and structure of the prevailing microbial communities existing in the digester at any given time.

Other factors influencing the fate of pathogens and antibiotic resistance genes occurring in waste during anaerobic digestion have been mentioned further in the manuscript under the section Inactivation of Bacterial Pathogens and their antibiotic-resistant counterparts. Overall, AD will help in curtailing gastrointestinal diseases caused by animal-borne pathogens as well as the dissemination of antibiotic resistance among bacterial pathogens. Therefore, anaerobically treating lignocellulosic waste (crop residues) together with animal manure (co-digestion) exerts synergistic effects on process stability and methane generation and enriches the microbial population, enabling contact between the substrates and the microbes or enzymes, as well as increasing the biodegradable components.

On the other hand, the pathogenic bacteria occurring in animal manure include *Salmonella*, *Shigella*, *Campylobacter*, *Yersinia*, and *Listeria* species, which are true pathogens causing gastrointestinal tract diseases (diarrhoea) in humans [61,62]. Diarrhoea is attributed to inadequate sanitation and hygiene, a situation very common in sub-Saharan African regions [63]. Bwire et al. [64] revealed that informal settlements in Bwaise, Kampala, have one of the highest rates of cholera in the country of Uganda, wherein outbreaks happen during the rainy season and are very closely connected with poor sanitation and wastewater discharge. This, therefore, highlights the need for continuous efforts to achieve and provide improved sanitation. In addition, Kariuki and Dougan [65] noted an underestimated emergency in the sub-Saharan Africa region due to antibiotic resistance among key bacterial pathogens of public health significance, which acted as a major hindrance to providing basic healthcare needs for communities. Moreover, Johnson and colleagues [66] have noted the high prevalence of human immunodeficiency virus (HIV) in South Africa, and such individuals rely on the consumption of antibiotics to boost their immunity. Also, it has been highlighted that HIV-positive diagnosed individuals are more vulnerable to developing enteric fever with frequent relapses [67]. These compounds further the public health implications of these enteropathogens should they enter the food chain due to accidental, unplanned, or deliberate release of the animal manure caused by rainfall, storms, etc. Interestingly, some countries are water-scarce countries (e.g., South Africa). Therefore, the existing water bodies need to be protected from pollution.

Globally, much of the population has limited access to basic public sanitation, energy, and fertiliser. In Kampala, Uganda, microscale (small-scale) anaerobic digestion has been considered as an on-site waste management solution, which creates an opportunity for low-cost waste management that is decentralised and produces beneficial co-products of renewable energy and organic fertiliser [68]. In South Africa, biogas digesters are principally constructed and installed in the Western, Kwa-Zulu Natal, and Eastern Cape provinces of the country, and some may not be functional or operational because of a lack of expertise and knowledge on the anaerobic digestion process [69,70]. Following World War II, economic and demographic development provoked an increase in agricultural production efficiency, which led to a simultaneous rise in large concentrations of livestock operations and commercial fertiliser production [71]. However, on some land, degradation has occurred due to erosion, desertification, tillage, and unsustainable agricultural practices, leading to a remarkable decline in productivity [72]. Moreover, the rising world population has increased the demand for food, requiring an increase in agricultural production. Consequently, farmers are encouraged to use manure rather than completely replace it with commercial fertilisers. Moreover, partial replacement of synthetic or commercial fertiliser with digestate may permit substantial savings in small-scale farms in low-income countries, considering the increasing costs of these fertilisers [73].

Animal manure has been employed since time immemorial, or for centuries, as a source of nutrients and organic matter for soils in agriculture for crop production [74]. Its composition (chemical and microbiological) and quality vary with animal type as well as the season of manure production, which has demonstrated profound effects on soil health in terms of bulk density, aggregate stability, infiltration, water-holding capacity, soil fertility, and biological properties [71]. The extent of manure’s impact on the soil is affected by factors such as the physical and chemical properties of the type, climate, rate, and timing of application. However, the presence of pathogenic microorganisms in animal manure poses potential risks to the health of animals, humans, and crops when applied to the soil. A plant’s health can be affected via the splashing of contaminated soil particles from the soil surface to the crops due to rainfall or overhead irrigation. In addition, contamination of food crops can occur through direct contact of the plant surfaces with animal manure added to attachment to plant surfaces and uptake through the roots of plants [75,76].

To avert or address these mishaps associated with the application of untreated animal manure for crop production, it would be beneficial to reduce the pathogenic microorganisms prior to application. Apparently, the presence of pathogens or other components in manure may compromise food safety, restraining the direct use of manure as an organic fertiliser. Accordingly, processes involving the recycling and recovery of manure seem to be the solution. Compared to raw substrates, digestates present notable advantages, including lower pathogen concentration and enhanced nutrient availability for absorption by plants, along with their slow-release characteristics, which considerably reduce the risk of water and soil pollution [77]. The digestate produced from the anaerobic digestion process can be employed as a biofertiliser after being subjected to further treatment techniques, post-treatment [73]. This is because it is recounted that the digestate still harbours some level of biodegradable organic matter as well as a certain level of pathogens, depending on the temperature at which the anaerobic digestion procedure was conducted. Thus, the quality of the digestate necessitates risk assessment [78], thereby making post-treatment procedures obvious.

Post-treatment is advantageous over pretreatment incorporated prior to anaerobic digestion because it targets only fractions that were not degraded during anaerobic digestion, but pretreatment focuses its actions on both the parts that are easy and difficult to degrade through the anaerobic digestion process [79]. The ideology behind post-treatment is that energy, money, and chemicals are only utilised on biomass portions recalcitrant to biological degradation to elevate treatment efficiency while reducing the expenses incurred during the treatment [12]. Several researchers have explained the significance associated with the post-treatment of digestate, both for the recovery of residual biogas potential, recovery of nutrients, and hygienisation of the digestate, thereby curtailing the environmental and public health threats associated with the direct application of digestate discharged from the digester or raw manure [12,80]. Romio et al. [12] recounted that reducing the residual methane potential from the final digestate has the tendency to minimise the risk of methane emissions during the handling, storage, and subsequent application of the digestate. Moreover, producing more biogas from the same substrate tends to decrease the demand for biomass feedstock (which could at times be costly or limited), and there could be a more efficient management of the resources of the biogas plant. Therefore, the recovery of biogas from the digestate has the capacity to render the whole anaerobic digestion process more environmentally and economically sustainable.

**Figure 1:**
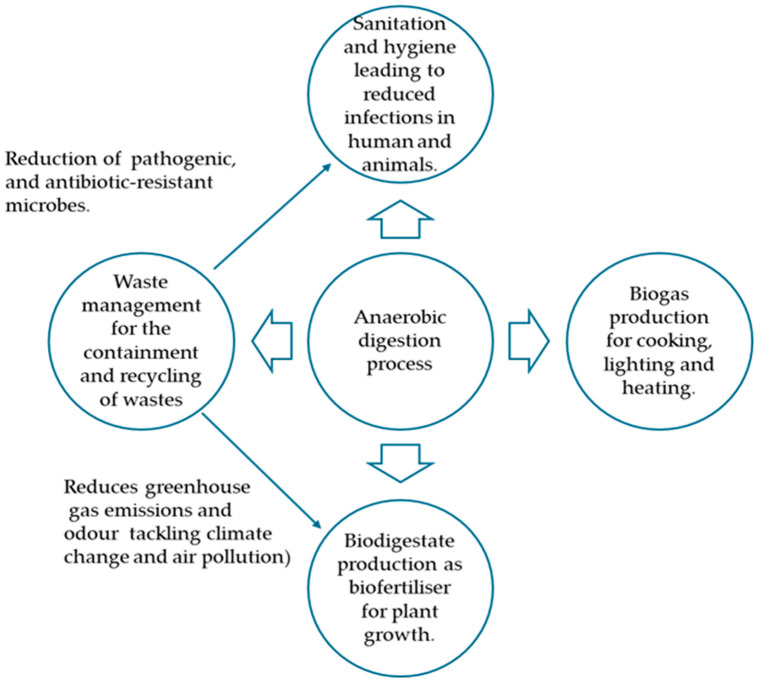
Manifold benefits of anaerobic treatment of organic wastes.

Against this background, this manuscript focuses on the application of anaerobic digestion technology as a tool to address the sanitation and hygiene crisis caused by the deliberate or accidental release of agricultural waste, especially animal manure. In addition, producing biogas from waste helps to alleviate the energy crisis, along with environmental pollution from the emission of greenhouse gases. More elaborately, this review provides a comprehensive overview of the anaerobic digestion process, shedding light on process dynamics, factors affecting the process, microorganisms, their interactions, and metabolic pathways, resulting in the production of biogas, improving environmental sanitation and hygiene, as well as producing digestate, a biofertiliser [81]. Overall, the many benefits of anaerobic treatment of agricultural residues are as presented in Figure 1. Precisely, it is a review on anaerobic digestion of lignocellulosic biomass yielding biogas as energy, alongside sanitising wastes by inactivating microbial components found therein, therefore reducing the contamination potential of the effluent or digestate discharged from the biodigester following the process. A thorough search into literature through PubMed, Google Scholar, ScienceDirect, and Google Engine was performed to gather salient information on anaerobic digestion of organic wastes. The search terms or phrases included anaerobic digestion, inactivation of bacterial pathogens in manure, post-treatment of digestate, antibiotics, antibiotic resistance, and co-digestion of waste, among others. Several findings registered by different researchers through different studies performed in different countries under different scenarios, while employing varying methods, have been assembled in a chronological fashion to emphasise similarities and differences pertaining to the significance of the anaerobic digestion process in terms of energy generation, sanitisation, and the post-treatment of the digestate to ensure its use as biofertiliser. In other words, it is a comprehensive review that synthesises knowledge from multiple fields covering comparative aspects of anaerobic digestion in terms of sanitation, hygiene, and energy production, and unifies it in a single place to present and address the problem of waste management. In this light, the current manuscript adds an extra step to the previously circulating knowledge in the field of anaerobic digestion, organic waste treatment, and renewable energy, as it simplifies and shows in detail the manifold benefits of treating organic matter contained in agricultural residue or waste.

The multiple benefits of the AD process are expressed as follows: First, the waste that would have ended up in landfills or incinerators generates energy, which is valuable for use following purification. As a waste management strategy, the process provides significant environmental advantages over traditional waste management systems (landfill and incineration) in that it reduces atmospheric emissions originating from uncontrolled decomposition of organic waste, decreases global warming potential, and adds to minimising fossil fuel consumption [52]. The primary interest in AD is the production of biogas, a renewable and sustainable energy source of high methane content. Interestingly, Rossi and co-authors [82] in their study recovered 145 NL biogas/d from dry thermophilic anaerobic treatment of the organic fraction of municipal solid waste recovered from a door-to-door collection in Florence, Italy. Niya et al. [14] reported that biogas obtained through AD demonstrates multipurpose applications, including combustion for heat production, electricity generation, and as a viable vehicular fuel. Similarly, Kowalczyk-Juśko et al. [83] noted the generation of 1.8 MW CHP power by a biogas plant in Poland, anaerobically co-digesting substrates (e.g., meat waste, industrial sewage sludge, digestate, 90,000 Mg/year) to produce biogas, which, after drying and initial desulfurisation, was burned in cogeneration engines (CHP). Also, the authors further highlighted the realisation of 80,000 Mg/year digestate from the quantity of multiple substrates being co-digested. Malet et al. [84] purported that developing renewable energy production is central to reducing reliance on fossil fuels and to reducing anthropogenic greenhouse gas (GHG) emissions. In detail, the authors explained that biogas production replaces fossil energy with renewable energy, thereby avoiding CO_2_ emissions and therefore offering additional benefits to climate action. Thirdly, the use of bio-digestate emerging from AD as a biofertilizer or an organic amendment limits the use of chemical fertiliser. Parra-Orobio et al. [85], in a study, noted that biodigestate obtained via a two-stage anaerobic digestion (acetogenic/methanogenic configuration) gave the best performance among others in terms of better physicochemical, microbiological, and parasitological characteristics, being a class B material, without exceeding the threshold limits for heavy metals, faecal coliforms (FC < 1000 CFU 100 mL^−1^), *Salmonella* spp. (0 CFU g^−1^) and viable helminth eggs (0 HE g^−1^). Moreover, Fernández-Rodríguez et al. [86] described the digestate as a mineral reservoir, containing meaningful quantities of ions and metals that are needed and valuable for plant development. As such, its use as an organic amendment is an environmentally sound and economically feasible treatment. Therefore, it fosters the development of a more sustainable agriculture [87]. Accordingly, Parra-Orobio and colleagues [85] found that the digestate presented with greater agricultural use in that, at a 5% dose application, it produced a germination index > 120%, attributing the effect to the presence of humic and fulvic acids and with N and P concentrations > 1%. In the same vein, Kowalczyk-Juśko et al. [83] noted the reduction in greenhouse emissions by replacing mineral fertiliser with digestate. The authors further commented that GHG emissions of 25.8–44.5 Mg CO_2_ equivalent were avoided when a total quantity of digestate was applied to an area of 1 ha annually. Apparently, the application of digestate can contribute to reducing the emission of gases [88]. The supply of carbon-rich digestate to soils aids in storing carbon in soil organic matter, thereby contributing to atmospheric CO_2_ removal. In addition, the use of nitrogen-rich digestate on agricultural soils functions in avoiding GHG emissions related to the production, distribution, and land application of synthetic nitrogen mineral fertilisers [84]. Finally, the microbial load in biowaste in terms of species level, types, and population is indicative of the potential to cause disease and infections. The infectious dose (defined as the estimated number of organisms needed to produce infection in 50% of normal adult humans), denoted as colony-forming units (cfu) to provoke infection in a susceptible host, is affected by the pathogen type/species, the route of exposure, and the status of the immune system of the intended host [89]. Thus, the infectious dose spans from as low as 10 cells to over 100 or 1000 s of cells based on the microbial species. When subjected to anaerobic digestion, the microbial fraction of the biowaste can become significantly reduced, wherein microbial inactivation is caused by several factors, including pathogen types, temperature, intermediate products, and operation modes. These factors could be described as the determinants of the inactivation efficiencies [90]. Interestingly, Tang and colleagues [54] noted a >6 log10 CFU/mL reduction in *E. coli* and a 2.37–3.87 log10 reduction in *E. coli* abundance in all tested conditions during the acidogenesis stage through the batch anaerobic co-digestion of food waste and waste-activated sludge and attributed such findings to the effect of pH. Consequently, such a decline in the bacterial load corresponds to a drastic drop in the potential of the biodigester to cause infection or disease. Moreover, the authors further mentioned the realisation of a CH_4_ yield of 256.7 mL/g VS.

## 2. Anaerobic Digestion

**Figure 2:**
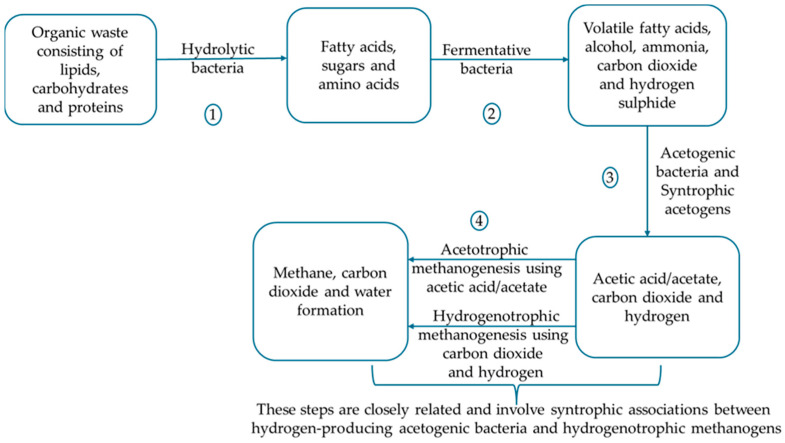
Anaerobic digestion process with different phases. Numbers 1–4 denote the four phases: 1—hydrolysis, 2—Acidogenesis, 3—Acetogenesis, and 4—Methanogenesis.

**Figure 3:**
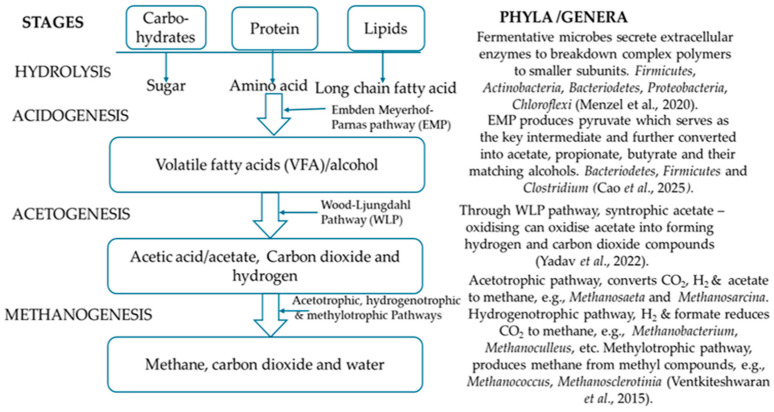
Annotated schematic of the anaerobic digestion process, highlighting the stages, pathways, and microorganisms involved. [17,20,104,110].

It is defined as the degradation of organic matter in a substrate in the absence of oxygen to yield methane, carbon dioxide, and other trace gases. The process is performed by the collaborative efforts and activities of four groups of microorganisms through four stages/phases, namely hydrolysis, acidogenesis, acetogenesis, and methanogenesis [91], with each stage and group of microorganisms producing metabolites that serve as the precursors for the subsequent phases [92]. The microorganisms involved in the process are categorised based on the phase at which they perform their roles, including hydrolytic bacteria, acidogenic bacteria, acetogenic bacteria, and methanogenic microbes [93]. Figure 2 represents the different phases in anaerobic digestion together with the category of microbes involved at each stage. For a detailed understanding of this process, an annotated schematic on AD, highlighting the stages, metabolic pathways, and the examples of microbes involved, is shown in Figure 3.

The anaerobic digestion process, also termed the biogas process or biomethanation, is a complex and controlled biochemical process that begins with the conversion of large polymers (lipids, proteins, carbohydrates) into monomeric or smaller subunits, including fatty acids, amino acids, and sugars. This conversion is brought about by hydrolysis through the activity of hydrolytic facultative/obligate anaerobic bacteria [94]. The hydrolytic bacteria make use of extracellular enzymes, including lipases, proteases, and cellulases [58]. Chang et al. [95] noted that accessibility to substrates, which in turn relies on chemical composition, greatly impacts the rate of this phase. In the next phase, the acidogenesis phase, the hydrolysed substances originating from the previous phase are subjected to degradation by acidogenic bacteria, converting them into volatile fatty acids, including acetate, propionate, valerate, butyrate, carbon dioxide, ammonia, and hydrogen. In the acetogenesis phase, acetogenic bacteria convert volatile fatty acids to acetate and hydrogen. Finally, the methanogenic archaea, in the methanogenesis phase, through two different pathways piloted by two distinct groups, produce methane. That is, acetoclastic methanogenic archaea ferment acetate substrate, or the hydrogenotrophic methanogenic archaea (carbon dioxide reduction pathway) reduce the substrate, carbon dioxide with hydrogen, or methylotrophic methanogenesis, wherein methyl compounds are converted to methane [96]. The acetoclastic and hydrogenotrophic/methylotrophic methanogenic archaea are vital for stable anaerobic digestion. Yuan et al. [97] noted that the generated biogas consists of 45–75% methane, 25–55% carbon dioxide, and traces of other gases, including hydrogen sulphide and water vapour.

Apparently, the process is not only gaining attention on a global scale due to its potential for treating organic waste [98], which reduces water quality and creates a danger to public health if not properly managed and contained, but it also produces biogas, a renewable source of energy in the form of methane. Silva and co-authors [99] affirmed its advantages, including a reduction in greenhouse gas emissions, alongside the decontamination and reduction in the volume of waste. In addition, it contributes to the circular economy as one of the end products. The digested effluent, or digestate, has the capacity to be reused as a soil fertiliser or improver owing to its composition (rich content of inorganic nitrogen and phosphorus) [100]. Nkoa [101] affirmed that anaerobic digestion can be exploited in seven key areas: (i) treatment of municipal sewage sludge, (ii) treatment of industrial wastewater from agro-food and fermentation industries, (iii) treatment of livestock waste, (iv) treatment of the organic fraction of municipal solid waste, (v) co-digestion of livestock wastes and the organic fraction of municipal solid waste, (vi) treatment of energy crops, and (vii) co-digestion of animal slurries with energy crops. This biological treatment method is regarded as one of the most favourable waste management approaches because its process parameters can be regulated or optimised to attain optimal digestion with respect to biogas productivity and digestate quality [11].

Anaerobic sludge digestion operates as a mechanism to promote the development of a sustainable society. For example, it strengthens the Sustainable Development Goal (SDG) of “zero hunger” (SDG2) through digestate or biosolids in food production or by handling methane and hydrogen to support “affordable and clean energy” (SDG7) [102].

Taking into consideration Figure 2 and Figure 3, the anaerobic digestion process recovers energy and materials from organic wastes to meet the principles of the circular economy. Anaerobic digestion is a microbe-driven biochemical process. Therefore, the interactions between the microbial communities are the core of the process [17]. The stability of this process and the subsequent generation of the biogas yield are greatly dependent on the synergistic interactions added to the appropriate balance of substrate flow between the different microbial communities [19]. Syntrophy is the basic mechanism that sustains microbial communities in AD, as the methanogenic archaea feed on the bacteria’s by-products [20]. The link between metabolic pathways and microorganisms is essential, as microbial interactions determine metabolic pathway differentiation in an anaerobic digestion process [103]. Cao et al. [20] explained that syntrophy in AD is governed by a mutually dependent thermodynamic relationship between bacteria and archaea (methanogens). In this light, a cascade of microorganisms constituting bacterial phyla, such as Firmicutes, Bacteriodetes, and Proteobacteria (the first three phases), engage in the breakdown of the organic matter in the wastes, producing intermediates that are finally converted into methane (at the fourth phase) by acetrotrophic, hydrogenotrophic, and methylotrophic methanogenic archaea. The breakdown at the very beginning of the AD process, termed hydrolysis, wherein complex polymers are disintegrated by fermentative microbes using secreted extracellular enzymes into monomers [104] is presented below:

(C_6_H_10_O_5_) n + nH_2_O = nC_6_H_12_O_6_ (enzymatic hydrolysis) (1)

where C_6_H_10_O_5_ denotes polysaccharides that are converted to simpler sugars, C_6_H_12_O_6_.

This justifies that the microbial species do not function in isolation but are involved in syntrophic interactions wherein one species lives off the metabolic by-products of another [105]. Moreover, the organisms at each stage depend on the end products of the previous, using them as precursors. In addition, the dominant population of organisms varies markedly, depending on substrate composition alongside environmental conditions. The process consists of four sequential stages. However, the acidogenesis stage stands as the key determinant of the metabolic pathways because the VFAs produced influence the subsequent reactions [17]. In other words, Zhou et al. [106] emphasised that the acidogenic phase decides the metabolic pathway of biomass conversion. In addition, facultative microbes utilise carbon and hydrogen to create an anaerobic environment during the acidogenesis phase. Each type of VFA formed at this phase adopts a specific metabolic pathway, which changes with the different conditions and dominance of the microbial strains or species. The divergence of the metabolic pathways through multiple channels in the acidogenic phase is the governing factor of formation along with the distribution of VFAs in AD [17]. This group of bacteria, acidogenic bacteria, may prevent methanogenesis as they grow much faster than the methanogenic group of organisms [107], and under specific operating parameters, they can provoke agglomeration of acidic intermediates, lower the pH, and inhibit methanogenesis [108].

C_6_H_12_O_6_ → 2CH_3_CH_2_OH + 2CO_2_ (2)

C_6_H_12_O_6_ + 2H_2_ → 2CH_3_CH_2_COOH + 2H_2_O (3)

C_6_H_12_O_6_ → 3CH_3_COOH (4)

C_3_H_7_O_2_N + 2H_2_O → C_2_H_4_O_2_ + NH_3_ + CO_2_ + 2H_2_ + ATP (5)

C_4_H_9_O_3_ + NH_3_ + CO_2_ + H_2_ + ATP (6)

4CH_3_COCOO^−^ + 4H_2_O → 5CH_3_COO^−^ + 2HCO_3_^−^ + 3H^+^ (7)

Also, remarkable is the acetogenesis phase in determining the overall efficiency of the anaerobic digester, because approximately 70% of methane is formed when the acetate is used by the microbes to form methane. In this phase, both acetate formation and degradation occur via the syntrophic process by two groups of bacteria; acetate-forming acetogens convert CO_2_ and H_2_ to acetate, while the syntrophic acetate-oxidising bacteria (SAOB) oxidise the acetate, converting it into H_2_ and CO_2_, functioning as acetate-degrading microbes [109]. Of great importance in maintaining the high efficacy and stability of the AD process is the interplay between SAOB, which produces hydrogen, and the hydrogenotrophic methanogens that consume it because some short-chain fatty acids, alongside propionate, can prohibit methanogenesis [107].

nC_6_H_12_O_6_ = 3nCH_3_COOH (acetate formation) (8)

C_6_H_12_O_6_ + 2H_2_O ↔ 2CH_3_COOH + 2CO_2_ + 4H_2_ (9)

CH_3_CH_2_OH + H_2_O → CH_3_COO^−^ + 2H_2_ + H^+^ (10)

In the final phase, known as methanogenesis, the products obtained from acetogenesis are utilised as precursors, and special microenvironmental conditions, including oxidation reduction potential (ORP) < 250, VFA accumulation < 2500 mg/L, and ammonia < 1000 mg/L, are required for their conversion to methane and other end products via the acetotrophic, hydrogenotrophic, and methylotrophic pathways [110,111]

CH_3_COOH = CH_4_ + CO_2_ (acetotrophic pathway) (11)

CO_2_ + 4H_2_ = CH_4_ + 3H_2_O (hydrogenotrophic pathway) (12)

2CH_3_CH_2_OH + CO_2_ = CH_4_ + 2CH_3_COOH (methylotrophic pathway) (13)

Anaerobic digestion has been widely accepted and employed as a form of energy recovery that aligns with the propensity to avoid, reduce, reuse, recycle, recover, and treat waste to curtail disposal and focus on the circular economy. In precise terms, Άlvarez-Fraga et al. [53] emphasised the triple role of this well-established biological process to include (i) production of biomethane in the form of biogas, a renewable energy source; (ii) waste treatment, sanitisation, and stabilisation; and (iii) generation of digestate, a nutrient-rich organic fertiliser. However, the advantages of this promising and sustainable process for treating waste can be annulled due to improper management of the residual digestate. This is because of the possibility of regrowth of microorganisms during storage if conditions are favourable, thus leading to the recontamination of the digestate [112].

Terminologies used in describing the anaerobic digestion process are diverse. Overall, the process can be described as psychrophilic, mesophilic, or thermophilic based on the temperature of operation, as well as batch or continuous according to the mode of feeding of substrate and as mono- or co-digestion depending on the number of wastes utilised [113]. The AD process can take place as a single digestion (mono-digestion) or multiple digestions (co-digestion), in scenarios wherein a single substrate or a collection of two or more substrates is treated, respectively, in an anaerobic biodigester for the purpose of biogas production [114]. In addition, the process can be performed in different modes and operated at different temperatures, as presented below:

Single-stage anaerobic digestion (AD): It is the conventional mode of anaerobic digestion, and, in the single-stage process, methane (CH_4_) is considered the key component of biogas products aside from the by-product, CO_2_. In addition, microbes associated with all the stages of digestion, including hydrolysis, acidogenesis, acetogenesis, and methanogenesis, are combined in a single reactor to establish a synergistic microbial consortium for the metabolism of organics [115]. Apparently, the different groups of microbes performing the four different steps of the process need different operating conditions in terms of nutrition and physiology. Therefore, it is critically challenging to balance the complex microbial community [116]. The AD process occurring via a single-stage system could be an effective and inexpensive choice when treating substrates that are easily biodegradable.

Double-stage anaerobic digestion: In the two-stage AD system comprising H_2_ and subsequent CH4 production, the methanogenesis step is physically restricted to occurring in the second stage [117]. However, both hydrogen (H_2_) and methane (CH_4_) are obtained in isolated stages that are conducted via unrelated conditions. Moreover, AD processes occurring in a two-stage form have shown more tolerance to higher organic loading rates. In scenarios where hardly biodegradable substrates are being treated, the two-stage anaerobic digestion process was more advantageous with respect to biohythane production. The organics are degraded to H_2_, CO_2_, and organic acids in the former stage and are further transformed to CH_4_ in the latter stage. Therefore, different microbes can be enhanced at their optimum conditions in different stages, thus demonstrating better performance [116]. Notwithstanding, Si et al. [118] noted that the microbial communities in the two-stage AD usually differ from those in the single-stage AD, but the abundance and activities of specific anaerobic microbes determine the efficiency of the former.

Psychrophilic digestion: It is an AD process that occurs at temperatures less than 20 °C and involves both psychrotrophs and psychrophiles. Colder temperatures exert effects on the affinity of microbes to substrates, resulting in a restrained ability to metabolise substrates necessary for growth. The effects can further be expressed as physiological constraints on the fluidity and integrity of the microbial cell membrane [119], alongside increased viscosity of the cell membrane, causing decreased rates in the movement and diffusion of vital nutrients and metabolites that are appropriate for growth and metabolism [120]. Akindolire and colleagues [120] substantiated that VFAs are produced during hydrolysis, acidogenesis, and acetogenesis by faster-growing and less cold-sensitive bacteria; meanwhile, cold-sensitive methanogens that are slowly growing cannot utilise acidic substrates rapidly enough, converting them to methane through methanogenesis. This occurrence leads to acidification and decreased buffering potential in anaerobic biodigesters, a situation that is commonly caused by inequality in acid utilisation and production kinetics [121]. Following these, acid accumulates in the biodigesters, resulting in low pH levels, which alter the environment, making it unstable for methanogenic activities by the prescribed methanogens [122]. Thus, the performance of cold-sensitive microorganisms is impacted at colder temperatures, resulting in longer fermentation cycles as well as lower methane yields during anaerobic digestion [123].

Mesophilic digestion: Anaerobic biodigesters are usually operated at mesophilic and thermophilic temperature ranges, as temperature plays a decisive role in their operation. The temperature ranges may cause operational, energetic, ecological, and economic benefits. Temperature is described as one of the crucial conditions in the milieu suitable for the stable anaerobic digestion process, exerting an influence both on physicochemical factors and microbiota [124]. The influence of temperature on biological processes is affected by both the activity and growth of different microbial groups and the enzyme activities, enzyme reactions, and substrate diffusion rates, which might respond differently to the chosen temperature levels [125]. Alterations in microbial structure occur with temperature due to different specific growth optimum temperatures and/or associated growth conditions [124].

The mesophilic process takes place at temperatures between 20 and 45 °C, and the microorganisms performing the process are termed mesophiles. Precisely, mesophilic digestion occurs as a more stable process, though resulting in lower biogas production rates. Its disadvantage is that it does not cause pathogen reduction to appreciable concentrations, producing Class A biosolids (i.e., a biosolid containing no detectable levels of pathogens [126]). The microbial population, or microbiota, in terms of species richness and diversity, mirrors the process stability and flexibility of anaerobic digestion, with higher species diversity in mesophilic than in thermophilic conditions [127]. Moset et al. [128] indicated that a sign of process stability in both the mesophilic- and thermophilic-operated digesters treating manure included the dominance of the members belonging to the order Methanosarcinales. The representative bacterial phyla in mesophilic digesters include Firmicutes, Proteobacteria, Bacteriodetes, and Chloroflexi, as opposed to Firmicutes, Proteobacteria, Chloroflexi, and Actinobacteria, which usually dominate among the bacteria [129].

In terms of methane-producing organisms, Methanosarcinales are typically found in digesters, but the species vary between mesophilic and thermophilic operations, with the genus *Methanosaeta* occurring in the former and *Methanosarcina* in the latter. Moreover, Chen [130] noted that the growth and degradation rates of microorganisms at thermophilic temperatures are two to three times higher than in mesophilic conditions. In addition, the study by Moset et al. [128] demonstrated higher organic matter degradation, higher pH, and higher methane (CH_4_) yield, as well as a better percentage of ultimate methane yield retrieved and lower residual methane emissions under thermophilic conditions.

Thermophilic digestion: This process occurs at temperatures between 50 and 55 °C, and organisms operating at such high temperatures are described as thermophiles. In a thermophilic digester, hydrogenotrophic methanogenesis dominates, involving the orders Methanobacteriales (e.g., Methanothermobacter) and Methanomicrobiales (e.g., Methanoculleus) [131]. Thermophilic treatment of chicken manure, a nitrogen-rich substrate, is often associated with higher ammonia levels, and the operation is faced with process disturbances unlike mesophilic processes [132]. Notwithstanding, thermophilic anaerobic digestion processes exert a better hygienisation effect [133], and from the sanitary perspective, it is more important to treat manure harbouring pathogens when compared to mesophilic processes [134]. Although at low temperatures, the process of anaerobic digestion usually fails to produce methane, processing organic wastes via psychrophilic anaerobic digestion may be more beneficial in terms of the economic and ecological aspects involved in the generation of biogas, particularly in regions faced with colder conditions. This is because conventional solutions, including heating systems or insulation, can be used [135]. In a nutshell, Table 2 shows the differences between psychrophilic, mesophilic and thermophilic AD based on different process parameters.

Batch operation: In this type of anaerobic operation, the biomass (feedstock/substrate and inoculum) is fed into the digester in a single load at the beginning of the process or once until all the raw material or feedstock or substrate is loaded, and the digester is sealed for the duration anticipated for digestion (i.e., retention time). All the digestate (the digested material) is only discharged or emptied at the end of the process (retention time), and therefore, the microbial load of the effluent or digestate is usually of lower magnitude as compared to its counterpart (continuous operation), since all the waste resides or is retained in the biodigester for the entire retention time indicated for the digestion to be completed [142].

Continuous operation: In this scenario, the substrate is fed intermittently as well as the effluent or digestate is withdrawn or moved out of the biodigester. Therefore, not all the portions of the substrate will spend sufficient time within the digester. Consequently, the microbial level of the digestate is often very high, as the withdrawn portion must have spent limited time in the biodigester, without being exposed to all the stages involved in the anaerobic digestion process, in addition to the environmental conditions (temperature, pH) and the resulting breakdown products generated through substrate degradation [142].

Wet/dry anaerobic digestion: In wet processes, the total solid (TS) in the anaerobic digester is ≤10%, and it embraces a homogenous distribution of feedstocks and seed sludges alongside the availability of biodegradable organic matter in the digester, hence culminating in great specific biogas production as well as VFAs [143]. Clearly, there is the addition of large volumes of water to maintain the TS < 10%; it involves continuous mixing, and the digested sludges are handled, requiring costly dewatering processes. Meanwhile, dry anaerobic digestion takes place with TS in the range between 20 and 40%; it involves little or no water addition and does not need any continuous mixing. In addition, this type of digestion permits the utilisation of smaller reactor volumes, as it can handle larger organic loading rates, unlike wet AD [82].

Manure management is vital for the sustainable use of plant nutrients, as disease-causing pathogens can be distributed with manure. However, on small farms, the appropriate methods of treatment should employ resources that are available on the local level and are inexpensive but subsequently or later on provide resources to the farmers without ridiculous effects. Anaerobic digestion is an efficient way of stabilising biowastes by treating them with microbial consortia in an airtight chamber (devoid of oxygen) to recover potential renewable energy along with nutrient-rich organic fertiliser (biofertiliser) for sustainable waste management [144]. Animal manures have been reported to be suitable substrates for anaerobic digestion, capable of facilitating the degradation of the organic matter in the biomass to yield methane (biogas) and carbon dioxide and traces of hydrogen, nitrogen, and hydrogen sulphide as well as a nutrient-rich fertiliser [145]. Therefore, anaerobic digestion technology plays a vital part in establishing a circular economy in agriculture and contributes to the Sustainable Development Goals of the United Nations [146]. In addition, anaerobic digestion of organic substrates, which leads to the production of biogas, stands as one of the most important pillars of the transition into the circular economy concept [90,147]. In other words, Ekstrand and colleagues [58] highlighted that because of the multiple functions attached to the process (anaerobic digestion) and its high-value outputs, the process is viewed as fundamental to attaining a circular bioeconomy. In this light, the deep-rooted technology advocates for the reduction in waste through recycling and reuse as it participates in treating diverse forms of organic waste and wastewater, ensuring their conversion for bioenergy recovery, organic biofertiliser production, and environmental protection. According to Yu and colleagues [148], the anaerobic digestion process has the capacity to further affect the economic and social aspects of sustainable agriculture, alongside environmental benefits. In addition, manure storage tanks constructed on farms may serve as a first step in the AD treatment line and/or for solids separation [149].

The approach needed to address the sanitation crisis globally is multifaceted, owing to the interconnected effects of culture, economics, policy, and human behaviour on sanitation and its relationship with access to water [150]. Globally, a quick rise in population growth, industrialisation, and urbanisation has led to the generation of large volumes of waste, causing disposal challenges [3,151]. Huge quantities of waste are produced with diverse constituents, including organic fractions, toxic metals, and emerging contaminants (biological and chemical) [152]. The composition and features of waste produced across different regions, municipalities, and countries are influenced by economic status, food habits, living standards, literacy rates, rituals, sources of energy used, and topographical and climatic conditions [153]. Similarly, Leelavathy et al. [154] and Pandey et al. [155] remarked that every waste has specific properties, and the different types of waste are based on their sources and origins. Zhao and Liu [156] added that the abundance of the pathogens fluctuates with process configuration and conditions of operation. The distribution of pathogens by way of agricultural applications or the direct discharge of inadequately treated/managed waste presents serious challenges to environmental and public health [156]. The diverse sources of biowastes harbour varying levels of different pathogenic microorganisms (relative abundances), including bacteria, viruses, fungi, and parasites, causing a potential threat of uncontrollable spread into surface water, groundwater, and soils [157]. Therefore, these biowastes act as sources of pathogens (waterborne and soilborne) and a source of antibiotic-resistance genes. Naturally, water is an essential component in the existence of every living organism; hence, the protection of water resources is of the utmost importance. It is an indispensable resource for human survival [158]; water bodies make up the essential segment of the biosphere and occur in complementary association with worldwide metabolically functional systems. The availability of fresh water is greatly exposed to effects originating from global climatic changes, increases in human population, urbanisation, and pollution [159]. However, freshwater bodies are scarce globally, and groundwaters are faced with a critical situation based on the impacts of unmanaged huge quantities of waste [160]. Precisely, rivers play an important role in domestic, industrial, and agricultural activities [161]; therefore, protecting the water quality of rivers is exceedingly important due to serious water pollution and the scarcity of water reservoirs worldwide [162].

However, through various human activities, the accidental or deliberate release of waste into the environment may result in environmental pollution or impacts, which have adverse effects on the ecosystem, climate, and society. The impact or level of pollution tends to vary based on the characteristics of the site, the prevailing conditions, and the type of activities practised [163]. The discharge of waste into the soil and water sources may lead to pollution of the ecosystems, posing a danger to every form of life, whether directly or indirectly [152,164]. According to Urooj and colleagues [162], water pollution can occur from a point or non-point source. Water quality is affected by both the chemical and biological content of the waste, causing rapid depletion and pollution, which eventually leads to the decline of available water resources for human consumption and the compromising of ecological integrity.

Ali and co-authors [60] emphasised that water pollution is a global issue, as contaminated water is considered the primary source of waterborne infections, although the problem is worsened in underdeveloped countries by poor sanitation and water management and the lack of access to safe drinking water. In developed countries, regardless of advances in water treatment and sanitation, outbreaks continue in places with limited resources. Lin and colleagues [158] emphasised that water pollution impacted human health significantly, even though the effects vary according to region, age, gender, and other differences in degree. Surface water becomes unsuitable for human consumption because drinking unsafe water, along with poor environmental hygiene, can lead to gastrointestinal illness, inhibiting nutrient absorption and malnutrition. Apparently, lack of water and sanitation services also increases the incidence of diseases. Accordingly, Ali et al. [60] reported that each year, millions of people are faced with gastrointestinal infections and other disorders (e.g., dysentery, diarrhoea, cholera, schistosomiasis, helminthiasis) caused by microbial pollutants, including bacteria (*Escherichia coli*, *Vibrio cholerae*, *Salmonella* spp.), viruses, and protozoa (*Giardia lamblia*, *Cryptosporidium parvum*). Apparently, human health is negatively affected by the presence of multiple pathogenic microorganisms in water. Mogane et al. [165] identified the Vhembe district of Limpopo province, South Africa, as a hotspot for diarrhoeal-related diseases, which remains a public health concern, especially among children under the age of 5 years. The district or area is faced with inadequate water and sanitation infrastructure; notwithstanding, the authors further revealed through their findings the association of *Campylobacter jejuni*, *Salmonella enterica serovar Typhimurium*, *Shigella flexneri*, and *Yersinia enterocolitica* in open defecation sites and animal waste with the contamination of water sources in Vhembe District, South Africa.

In terms of soil pollution, agricultural waste and biosolids have been in continuous use as fertilisers or soil amendments on agricultural land. The traditional way of managing animal manure is direct application on agricultural land via different application strategies. It is one of the most cost-effective methods of control of pathogens. The ideology is that the spreading of waste on the surface of the soil exposes microbial pathogens to sunlight (UV light), high temperature, drying, freezing, thawing cycles, exposure to oxygen, and ammonia [166]. The exposure of the bacterial pathogens in the wastes to achieve thermal reduction in pathogens may reduce some pathogens, but the risks to human health persist. Before any interactions with the soil surface environment can cause a reduction in the numbers of the pathogens, the pathogens associated with biowastes in agricultural fields may enter the food chain. The major pathogens found in both animal and human waste are common in the different types of waste, though they may be different at the level of the strain/species. The species differ considerably from each other in terms of the number of viable cells necessary to cause diseases; therefore, some are highly infectious in that ingestion of fewer than 10 cells by a vulnerable or susceptible host might lead to infection. For instance, ingestion of inadequately washed ready-to-eat vegetables and windfall apples on soils applied with biowaste. Leafy vegetables and root crops in very close contact with soil surfaces may be of great danger to the health of the people. Individual pathogens on the soil surface may equally have direct contact with the bodies of humans, posing a risk to human health; moreover, soil and humans have shared a long and intimate relationship (Samaddar et al., 2021 [167]). Soil pollution, unlike other forms of environmental pollution, is concealed and accumulated as the soil exists as a microenvironment that is conducive for the growth and proliferation of bacterial communities, with its physical and chemical characteristics influencing the structure of these communities [168]. As opposed to other microbes, pathogens occurring in the soil environment are more adaptable to changes in the environment and demonstrate a stronger ability to compete for survival resources [168]. Soil pathogens are biological contamination posing possible threats to the soil’s ecosystem, plant health, food security, and human health [169].

The ability of microorganisms to search for water and food might aid their movement from the surface of the soil into the soil, which can occur via horizontal or vertical transportation. The transportation of microbes is affected by soil properties, pathogen type, land cover, and weather. Li and colleagues [169] commented that the presence and reproduction of microbial pathogens in the soil environment have tremendous negative effects on soil security and human health in different regions of a country and across the world. Within a particular country and from an urban to a rural setting, substantial differences in terms of abundance and species of soil pathogens, alongside the network structure of the soil microbial community, were observed based on the different degrees of pollution [168]. However, this practice (land application of animal manure) is associated with serious adverse effects of environmental contamination, affecting both the soil and all its components/properties as well as humans because of surface runoffs caused by heavy rainfall and floods into water bodies. Soil fertility is impacted by the presence of pollutants (chemical or biological) in the waste [162]. According to Somadas et al. [170], the presence of biowaste impacts soil properties via soil contamination, bioaccumulation in crops, groundwater contamination, and changes in soil characteristics.

It is a fundamental right for basic health to have access to clean water and proper sanitation. The global burden of sanitation-related diseases and access to safely managed waste call for attention and strict monitoring of waste management systems. Therefore, developing a technology that combines management, treatment, energy recovery, and the production of green energy from biowaste is necessary [27]. In this light, anaerobic digestion is regarded as one of the waste management processes that impacts public health positively. The process will improve soil and water quality by curtailing or mitigating the above-mentioned negative effects of untreated waste in the different ecological niches. In this light, the process results in the inactivation of pathogens in the waste, significantly decreasing the number of viable pathogens, including bacterial indicators (*E. coli, Salmonella*) and other microbial groups (e.g., viruses and parasites). Thus, reducing the pollution potential of anaerobically treated waste. Secondly, the anaerobic digestion process causes the breakdown and killing of infectious agents owing to the microbial activity and environmental conditions (temperature and retention period) within the biodigester. Thirdly, AD leads to the production of a safer byproduct (pathogen-free) or digestate that might harbour a reduced number of pathogens, which in turn improves the safety of water and soil. Generally, prior to anaerobic digestion, the level of biological contaminants, including microorganisms alongside their antibiotic-resistant counterparts, is usually high, endowed with a great chance of causing contamination of soil and water, leading to the transmission of diseases through the water and food chain. However, following the process, there is a drastic loss of such potential in contaminating surface and groundwater sources often used for drinking, adding to a resulting better waste management system, especially in areas with inadequate sanitation infrastructure, by stabilising waste and reducing the overall burden of disease. Therefore, the digestate, which is termed pathogen-free, when applied as a fertiliser on crops to improve growth, minimises the risk of infectious diseases that can be transmitted via contaminated food and water. Apparently, the possibility of the transfer of pathogens from the soil to the food is prevented. Elaborated benefits of the application of digestate are presented further in a section of this manuscript.

The practice of matching anaerobic digestion with post-treatment is observed as a technology of great promise, which contributes successfully to addressing the complex global sanitation problem [171]. The production of biogas via anaerobic digestion has demonstrated direct benefits to the second pillar (reducing the cost of product processing) and indirect benefits to the remaining pillars (energy consumption and employment) of the circular economy. Yilmazel and colleagues [172] stated that anaerobic digestion represents the channel through which value-added products, including phosphorus, volatile fatty acids, and nitrogen, can be recovered. Therefore, it is a very crucial step in the expansion of a circular economy in treatment plants receiving wastewater.

### 2.1. Factors Affecting Anaerobic Digestion

There are numerous aspects that can influence both the stability and productivity of the process, termed anaerobic digestion. No matter what the choice of the substrate, it is paramount to constantly monitor the parameters of the anaerobic digestion. The efficiency of such a process producing biogas is often interlinked and includes organic loading rate, temperature, hydraulic retention time, substrate characteristics, pH, carbon-to-nitrogen ratio, micronutrients, mixing rate, and moisture content [173]. The factors are described alongside their optimal ranges, and their effects on biogas yield as presented in Table 3. The complexity of the process involves different groups of microorganisms with various environmental and nutritional requirements for growth. Therefore, adjusting one or more of the operating conditions will affect the growth and activity of the microorganisms, which will in turn determine the yield and quality of the biogas and digestate [174]. 

### 2.2. Microorganisms Involved in the Anaerobic Digestion Process

Metagenomics is a powerful technique in studying the composition of microbial communities in terms of structure, function, and dynamics of an anaerobic digestion system, facilitating optimisation of the biogas process, enhancing stability, and improving its overall performance [14]. The complex community of microorganisms that convert wastes into biogas through anaerobic digestion is collectively termed “microbiota”, performing a series of biological reactions [14]. The microbiota involves a great diversity and relies on several factors, including feedstock type, seed inoculum, temperature, granulation, aeration, mixing speed, pre-treatment type, digester design, organic loading rate (OLR), solids retention time (SRT), and hydraulic retention time (HRT) [15]. Owing to the complex and heterogenous ecosystem and the massive diversity of uncharacterised microbes in anaerobic digesters, the word “black box” is usually used in describing these anaerobic digesters, and the AD microbiome is known as “black matter”. The entire microbiota in an anaerobic digester system is essential for the various phases of the AD, along with stabilising the redox potential of the system [15].

The efficiency of the complex biological process, anaerobic digestion, is greatly reliant on the sensitive nature of microorganisms. The makeup of the prevailing microbial community added to their activities relies on both the external operational parameters and internal conditions of the environment of the digester [109]. Therefore, the performance of these microorganisms can be affected by several factors, including substrate type, process environmental conditions (e.g., pH and temperature), bioreactor design, operational modes, and access to the medium [186]. The collaborative activities of various microorganisms or microbial communities are eminent in producing biogas and in maintaining a stable anaerobic digestion process [191]. The concerted activities of two groups of microorganisms are necessary for the bioconversion of organic waste into biogas, comprising methane, water, and other gases in trace amounts. These groups include bacteria and methanogens [192]. In general, these microorganisms (e.g., hydrolytic bacteria, acidogenic bacteria, acetogens, and methanogens) work interactively in four complicated and interdependent biochemical reactions, degrading organic matter into biogas [15].

Acetogens are strict anaerobes that perform either autotrophic nutrition (using CO_2_ or CO as a carbon source for cellular synthesis) or heterotrophic nutrition (utilising formate or methanol as the sole source of C) and are responsible for converting the end products of the acidogenic phase into acetic acid, CO_2_, and H_2_ [193,194]. In the acetogenic phase, both acetate formation and degradation occur via the syntrophic process by two groups of bacteria: acetate-forming acetogens, which convert CO_2_ and H_2_ to acetate, while the syntrophic acetate-oxidising bacteria (SAOB) oxidise the acetate, converting it into H_2_ and CO_2_, functioning as acetate-degrading microbes [109]. Of great importance in maintaining the high efficacy and stability of the AD process is the interplay between SAOB, which produces hydrogen, and the hydrogenotrophic methanogens that consume it, because some short-chain fatty acids, alongside propionate, can prohibit methanogenesis [107]. Similarly, the acetate-forming acetogens produce acetate and hydrogen from organic acids and carbohydrates; the huge quantity of hydrogen decreases the pH of the system. Therefore, it can be consumed in either of the two ways: firstly, for the formation of methane, and secondly, for the formation of long-chain organic acids (e.g., propionic acid and butyric acid). Propionate and butyrate are the two VFAs for acetate production. However, their conversion to acetate cannot occur spontaneously due to the Gibbs free energy of the reaction [194]. In the anaerobic digesters, the Gram-negative, motile, and strictly anaerobic bacterium, *Syntrophobacter wolinii*, metabolises propionate to acetate. However, if it does not occur, the metabolite is bound to accumulate in the cells of this bacterium. The accumulation of propionate in the system is an indicator of stress; likewise, butyrate. In this light, the syntrophic relationship between propionate-degrading bacteria and methanogens is crucial, such as the association between *Syntrophobacter* and *Methanobrevibacter*, to ensure the stability of the process. The concentration of VFAs in the system may vary based on the feedstock, initial inoculum, operating conditions, and reactor configuration. Usually, VFAs are produced in the AD process, and the high concentration of these acids due to their continuous accumulation in the system decreases the pH, resulting in souring and ultimate failure of the system. Propionic acid produces a stronger disruption effect than the others. The removal of H_2_ from the digesting system can take place through hydrogenotrophic methanogenesis, sulphate-reducing bacteria, and homoacetogens [107].

It is apparent that interspecies hydrogen transfer is crucial in syntrophic relationships because it prevents the stockpiling of H_2_ as it is utilised by methanogens to reduce CO_2_, in addition to helping the acetogens to grow because they can survive at very low concentrations of H_2_ [195]. In general, keep the balance of the anaerobic system. Alongside interspecies hydrogen transfer, interspecies formate transfer occurs in the syntrophic relationship that exists between acetogens and methanogens. The mechanisms governing these transfers are recognised as indirect or mediated interspecies electron transfer (MIET). Interspecies H_2_ transfer occurs between two cells, acetogens and methanogens, in syntrophic interactions, since both do not have the capability to oxidise organic matter individually. Consequently, they exchange electrons through hydrogen transfer to perform the degradation. Hydrogen is a small molecule that diffuses easily, but its concentration is paramount to acetogens and methanogens. Owing to its low solubility, the transfer distance of H_2_ is low (10 μm). The close association of acetogens and methanogens in anaerobic granules, soils, and aquatic systems is preferred. Hence, hydrogen is the most common electron carrier [195]. On the other hand, interspecies formate transfer seems favoured when the distance between microbial cells is higher than 10 μm. Nevertheless, the rate of interspecies formate transfer is higher than that of interspecies hydrogen transfer, and the former mechanism tends to play a vital role in the syntrophic propionate-degrading co-cultures.

Direct interspecies electron transfer (DIET) is a phenomenon whereby electron transfer takes place from one cell to another without being mediated by any reduced molecules, whether hydrogen or formate. The electron transfer between the two cells powerfully relies on the durability of the cell contact; therefore, it explains the syntrophic relationship of the microbial cells in the anaerobic aggregates. This mechanism is most likely to occur when the hydrogen partial pressure is increased. Some of the distinctive features or advantages associated with DIET include higher speed of electron transfer and no need for hydrogen/formate shuttles, as it occurs through bacterial pili, cytochromes, or nanowires (cell–cell contact) among syntrophic microbial communities [196]. According to Wang et al. [196], the rate of electron transfer in DIET is determined by interspecies distance, the number of cytochromes and nanowires, microbial community resistivity, and cell-nanowire cofactor electron transfer rate constant.

Sulphate-reducing bacteria (SRB) represent another group of anaerobic microorganisms living in an anaerobic digester and perform a significant role in AD in that they can inhibit methanogenesis as they compete with methanogens for the substrates, hydrogen, and acetate to reduce sulphate with the methanogens [197]. They may live in syntrophic association with methanogens to metabolise propionate or butyrate. Two major types of SRB occur, performing either of these functions: oxidising the substrates to acetate, and examples include *Desulfobulbus*, *Desulfomonas*, *Desulfotomaculum*, and *Desulfovibrio*, and others can oxidise organic acids, including acetate, to CO_2_. In this category, the genera include *Desulfobacterium*, *Desulfobacter*, *Desulfosarcina*, *Desulfococcus*, and *Desulfonema*.

Microbial interactions also occur during methanogenesis. The methanogens belong to the domain Archaea, and the most identified phylum is Euryarchaeota, consisting of hydrogenotrophic, acetoclastic, and methylotrophic methanogens, which represent the three methanogenesis pathways for methane production. The hydrogenotrophic pathway produces approximately 28% methane content, while the remaining fraction (72%) originates from the activities driven by the acetoclastic pathway, but the proportions may be influenced by the substrate type [198]. The methanogens are described as strict anaerobic microbes that are highly sensitive to environmental parameters, for example, exhibiting intolerance to oxygen. In addition, this group of microorganisms demonstrates diversity in morphology and physiology, giving rise to seven orders, namely, Methanosarcinales, Methanopyrales, Methanococcales, Methanomicrobiales, Methanobacteriales, Methanomassiliicoccales, and Methanoplasmatales [199]. Harirchi and colleagues [15] noted that most of the microbial communities engaged in anaerobic digestion are prokaryotes, even though some eukaryotes (fungi and protozoa) are also involved in the process. Based on cytochrome, methylotrophic methanogens can be differentiated into two groups: strict hydrogen-dependent, i.e., those lacking cytochrome, and those harbouring cytochrome, thus endowed with the ability to oxidise methyl groups into CO_2_ [200].

Granule formation is one of the vital microbial interactions during methanogenesis in anaerobic digesters, but depending on the digester type and the reactor loading rates, the mechanism of granule formation may vary across the different types. Specifically, microbial granule formation is a strategy utilised by microbial cells in the digester to protect themselves against stressful environments [15]. A granule consists of three layers and resembles a filamentous consortium through which fluids and gases flow slowly. Examples of microbes forming granules include Methanosaeta, Gram-positive organisms with low G+C content, among others [201]. Moreover, Amani et al. [202] narrated that micro-spatial structures formed through granules and extracellular polymeric substances (EPSs) are of relevance for providing niches for different microbial communities for metabolite exchange among the microbes living in the granules, thus maintaining the function of the communities.

In addition, the type and level of microorganisms available for the anaerobic digestion process depend on the substrate types. Animal manure is made up of pathogenic microbes and parasitic eggs, demonstrating zoonotic concerns; the manure tends to vary in composition, affected by farm management, season of production/collection, livestock feeding, geographical location of the farm (environmental conditions), animal health, animal species, and physicochemical composition of the manure or manure storage [203].

Regarding the level or concentration of bacterial pathogens in the substrate (animal manure), microbiological analysis can be performed to detect and enumerate the bacterial pathogens, especially the major zoonotic bacterial species, e.g., *Listeria monocytogenes*, thermotolerant *Campylobacter* species, and *Salmonella* sp., as well as targeting faecal indicator bacterial species, e.g., *Escherichia coli*, *Enterococcus faecalis*, *Clostridium perfringens*, etc. This can be performed using culture-based techniques and molecular-based techniques (i.e., direct plate count or enrichment prior to selective plating or enrichment before polymerase chain reaction) [204] and expressed as colony-forming units per gram (cfu/g) or colony-forming units per millilitre (cfu/mL), or most probable number per gram (MPN/g), giving an indication of the sanitary efficiency of the anaerobic digestion process [205]. On the other hand, Thakur and colleagues [206] highlighted that the parasitic load can be measured by counting eggs per gram (EPG). Notwithstanding, the authors further explained that the concentration of selected bacterial pathogens tends to be reduced while monitoring as the waste undergoes anaerobic digestion treatment. The magnitude of the reduction seems to vary, but it depends on the initial contamination of the substrates used to charge or feed the biodigester. Indeed, Manyi-Loh and Lues [204], who evaluated the level of bacterial pathogens via monitoring of the co-digested medium (pig manure and sawdust waste) during psychrophilic anaerobic digestion, registered initial counts of 2 × 10^6^, 7 × 10^4^, 3 × 10^5^, 9 × 10^5^, and 1 × 10^4^ cfu/g of *E. coli*, *Salmonella*, *Yersinia enterocolitica*, *Campylobacter*, and *Listeria monocytogenes* to concentrations lower than the detection limit (DL = 10^2^ cfu/g substrate).

In terms of microbial composition, several molecular-based methods, including metagenomics, 16S rRNA amplification, and next-generation sequencing, have been employed to outline the different bacterial classes, genera, and species, as well as methanogens. Zheng and Li [116] reported that the functional bacteria categorised under the phyla Firmicutes, Bacteroidetes, and Proteobacteria were extensively identified in AD processes: the phylum Firmicutes is engaged in hydrolysis, acidogenesis, and acetogenesis phases of a broad spectrum of substrates. The rise in temperature and the change in pH that occur when the substrate is converted to end products during the anaerobic digestion process are involved in the destruction of pathogenic microorganisms. The different categories of microorganisms are affected differently; the total bacterial counts are reduced greatly with the advancement in time, based on whether the operating temperature is in the thermophilic or mesophilic regimen. The majority of infections can survive longer at mesophilic temperatures (30–42 °C) as opposed to thermophilic temperatures (50–55 °C) [206]. Salsali et al. [207] explained that a rise in temperature from 37 to 70 °C causes an increase in the fluidity and permeability of the cell, resulting in rapid diffusion of harmful substances into the cytoplasm, slowing down cell growth. Consequently, it inhibits the microbial pathogenic population.

The level of the microbial population, along with the microbial communities involved in the anaerobic digestion process, depends on the type of substrate, temperature, and pH conditions; the mode of operation (batch or continuous); the stage of the process; and the design of the anaerobic digesting unit. Similarly, Steinberg et al. [208] noted that the microbial community structure of an anaerobic digestion system can be determined by operational factors such as temperature, substrate type, sludge retention time, ammonia concentration, and organic loading rate. High phylogenetic resolution and good taxonomic classification at all ranks are essential in providing insightful links between the anaerobic digestion community and its performance [209,210]. High phylogenetic resolution can be obtained by using amplicon sequence variants. Accordingly, Cai and colleagues [98] registered an overrepresentation of genes encoding carbohydrate and protein metabolism functions during the treatment of industrial wastewater in a biodigester in Guangzhou, China, while an overrepresentation of genes encoding functions related to fatty acids, lipids, and isoprenoids was found with the treatment of municipal sludge in Shek Wu Hui, Hong Kong. The authors further stated that the findings reflected the plant’s feedstock.

Considering the temperature of operation, the microbial population involved in the digestion process can be categorised into psychrophilic, mesophilic, and thermophilic microorganisms. Psychrophilic microorganisms require longer retention times, ranging from 70 to 80 days, to convert the substrate into biogas as they grow and function in the temperature range of 10 to 25 °C. Also, mesophilic organisms grow and operate in the temperature ranges above those of the psychrophiles, occurring at 30 to 40 °C, and require 30 to 40 days to transform biomass into biogas. Thermophilic organisms grow and operate at temperature ranges higher than the previously stated two temperature regimes, in the range of 45 to 60 °C, during which they require a shorter retention period to generate biogas from substrates in 15 to 20 days [11]. Regardless of the temperature of operation of the process, biogas is produced from the concerted actions of three physiological groups of microorganisms, viz., hydrolytic-acidogenic bacteria, syntrophic acetogens, and methanogenic archaea. It is paramount to have knowledge of the microbiome of an anaerobic digesting system, including mechanisms of interspecies interaction, metabolic capacities of microorganisms, and the degree of functional redundancy within a community, for the optimisation and steering of the process of anaerobic digestion [11].

## 3. Global Perspective on Energy, Sanitation, and Hygiene and Mitigation Through Anaerobic Digestion of Lignocellulosic Wastes

### 3.1. Energy

Fossil fuels (coal, oil, etc.) serve as the conventional energy resources in most developing countries, including South Africa, and even in developed countries. These are non-renewable supplies that are equally tallied with certain mishaps, including increased cost prices, environmental and health pollution, and challenges of future availability [99,211]. These conventional energy source fuels are rapidly declining and are unable to meet the ever-rising energy demand of the population. The future of every country is bleak with sole reliance on any fossil fuel as a conventional source of energy. In addition, several countries employ wind, solar power, and hydropower as alternative energy sources, which are equally faced with challenges [94]. There is a great necessity to produce and make advancements in sustainable energy resources because of the fixed nature of fossil fuels and the greenhouse gas (GHG) emissions associated with their use, and the transition to a recycling-based society should be promoted [193]. Recently, there has been a need to seek alternative or complementary sources of energy, of which lignocellulosic biomass is envisaged to address the mishaps associated with finite energy sources. However, renewable alternatives, for example, biogas derived from waste biomass, could serve as adequate replacements, delivering solutions for power and heat generation [212]. Adewuyi [1] advocated that presently and in the future, lignocellulosic biomass, which is plant-sourced biomass, is a very significant substrate needed for many biotechnological processes, ensuring sustainability and producing renewable energy sources. Based on their plant nature, the waste is replenished naturally. Therefore, they are inexhaustible as they contain energy-stored compounds produced via the natural process of photosynthesis in the presence of sunlight. Huge volumes of these wastes are generated annually, and they are most abundant, consisting of significant levels of cellulose and hemicellulose that must be subjected to practical applications. This implies identifying possible uses of these materials via converting them into useful products, creating a win-win situation, which can equally be described as a waste-wealth application [1].

Owing to the mismanagement of this valuable waste, agriculture is regarded as the major contributor to climate change via the emissions of greenhouse gases and pollution of the air, soil, and water. Nonetheless, agriculture is the key driver of the economy of some developing countries, such as South Africa, resulting in huge quantities of animal manure. Also, in the European Union, livestock production is high, and Spain and Germany are noted as the biggest producers of pigs, while the bovine number is greatest in France [213]. In addition, China is one of the major livestock and poultry-producing countries globally, producing more than 4 billion tonnes of animal waste that are discharged each year [74]. Searchinger and colleagues [214] highlighted that the agricultural sector is responsible for approximately 40–60% of methane emissions globally. Seemingly, Wang et al. [196] mentioned that agricultural residues, including vegetable and crop residues and livestock manure, are among the most abundant organic waste worldwide. Conventional agricultural and food production systems usually produce huge quantities (millions of tons) of biomass energy resources in the form of agricultural waste, poultry, and livestock manure [122]. Approximately 12 tonnes of crop residue and 121 tonnes of animal manure are produced a year by a South African smallholder farm [215]. Specifically, poultry production is observed as one of the most rapidly growing sectors throughout the globe, as poultry is the major source of protein and the demand for its products is on a steady rise, caused by population growth, urbanisation, and changing dietary habits. Owing to a huge demand for poultry products and the corresponding increased production, the quantity of waste originating from this sector tends to increase day by day [206]. The degradation of organic wastes causes extensive contamination and deterioration of the ecosphere [179]. Precisely, this waste, when not properly managed, might decompose into the environment, resulting in gross pollution. The unhindered methane production from the degradation of the organic waste under anoxic conditions largely impacts the climate, causing a crisis [123]. The utilisation of sustainable agro-energy systems integrating crops, livestock, and bioenergy production has received profound interest and attention from farmers [11]. These wastes equally represent estimated biomass feedstock or substrates available for anaerobic digestion, as they possess a potential energy value that can be harnessed to produce biogas via anaerobic digestion.

On a global level, the World Biogas Association [216] recounted that more than 182,000 biodigesters are operated worldwide on various scales, and these showed a significant increase in numbers in recent decades. Moreover, approximately 84% of farms are operated by smallholder farmers, and millions of these farmers, especially in developing countries, have adopted biogas digesters for biogas production due to easy access to food waste and manure [217]. The adoption and development of biogas digesters have not taken full toll in African countries because of low temperatures that occur as one prominent factor affecting biogas generation during the winter period [135]. In some African countries, e.g., including South Africa, Ethiopia, Uganda, and Kenya, about 350, and over 10,000, 11,000 and 14,000 small-scale digesters have been installed, respectively [218]. In other sub-Saharan countries, including Kenya, the use of anaerobic digestion to limit the spread of faecal indicator organisms (FIOs) to a wider environment is of growing interest [76], and findings highlighted significantly low counts of FIOs in anaerobically treated cattle manure in both the inside and outside environments of households having installed biogas digesters [219]. According to Akpan et al. [220], the lack of awareness and suitable configuration of the treatment system could be the reason anaerobic digestion of food waste and animal manure is not a common practice in Nigeria. Notwithstanding, China and India are the two developing countries in which the largest numbers of well-developed small-scale digesters have been installed; these have been shown to improve the likelihood of the population living in rural communities [122].

In addition, the United States Environmental Protection Agency [221] reported more than 2100 biogas plants in the USA, of which 250 were farm-based plants digesting livestock manure. Similarly, Eurostat [213] stated that approximately 74% of primary biogas energy output was recovered from the anaerobic treatment of agricultural wastes, animal manure, and energy crops in biogas plants built in Germany. Within the European countries, Germany stands as a leader with close to 11,000 anaerobic digesters at farm scale, digesting a blend of animal manure and other co-substrates, including energy crops [222]. In the developed countries, agricultural biogas plants have increased in some European countries over the past decades [203]. Of the sampled countries, Germany has the maximum number of anaerobic digestion plants at 9989, followed by Switzerland (632), France (574), and the UK (545). Across the European countries, Άlvarez-Fraga et al. [53] published an approximate 209 TWh power generation capacity from biogas in 2018, which represented a fraction (7.4%) of the total net electricity generated. The annual energy yields from anaerobic digestion plants are 54,630 and 21,754 GWh/yr in Germany and the UK, respectively, followed by 2908, 2686, and 2169 GWh/yr in the Netherlands, France, and Brazil, respectively [213]. Similarly, the Swedish Energy Agency [223] remarked that 2.1 TWh of biogas is produced yearly from various wastes as follows: 35% from sewage sludge digestion, 49% from co-digestion of food waste and industrial waste, and the remaining fraction (16%) is from agricultural rest products and landfills.

Low-tech biodigesters are sustainable technologies employed in treating organic wastes, which lead to the production of clean energy (biogas) and biofertilizer (digestate) utilised by rural communities in low-income countries, endorsing the fulfilment of Sustainable Development Goals 6 (clean water and sanitation) and 7 (affordable and clean energy) [73,224]. In terms of electrical energy generation, Jaman and co-authors [225] projected measurements at an on-farm scale of 372 kWh, 382 kWh, and 518 kWh per day through the valorisation of agro-food waste, including food waste, cow dung, and co-digestion of food waste and cow dung, respectively.

#### A Comparative Analysis of Anaerobic Digestion Adoption, Technological Maturity, and Socio-Economic Benefits Between Developed and Developing Regions

Waste management (WM) practices vary drastically across the different regions of the world due to differences in income level, urbanisation, and population density [226]. Municipal solid waste (MSW) encompasses food and yard residues; paper, cardboard, fabrics (lignocellulosic waste), plastic, metals, and glass [227]; notwithstanding, the largest fraction of MSW is the organic fraction, and the quantities of MSW produced tend to vary across high-, middle-, and low-income countries [226]. Nevertheless, Piadeh and co-authors [228] highlighted that huge quantities of municipal organic waste occur in many developing countries, including Africa, the Middle East, parts of South America, and Southeast Asia. Therefore, different waste management processes are embraced to manage the waste. The inclusion of MSW in the United Nations Sustainable Development Goals (SDGs) recognises it as a critical sustainability challenge [229]. Overall, to ensure global sustainability, a shift is observed from a linear to circular bioeconomy, whereby MSW substitutes as inputs, and emissions (e.g., GHG) and energy leakage are minimised [230]. Waste management practices tend to be more sustainable as income levels rise, since these nations can invest in sustainable WM technologies. Globally, AD is a vital and essential approach, in high demand as a municipal organic waste management technology, offering the reduction in pollution and the production of biogas and fertilisers [231]. Accordingly, AD harbours potential pathways that effectively contribute to the United Nations Sustainable Development Goals (SDGs).

AD technology has received great attention based on its wide applicability in climate and environmental protection and its potential impact on the economy. Okaiyeto [232] expressed that the application of AD technology varies greatly across regions. There is an observed widespread adoption of this technology that has led to a mature industrial chain formed in Europe, especially in Germany and Nordic countries, owing to the stringent environmental regulations and renewable energy policies [233]. Seemingly, the prevailing technological innovations and advancements in pretreatment methods have propelled the adoption of AD in the United States [234]. In this region, growing interest has been built in adapting the technology for smaller farms, and a greater variety of feedstock is being driven by the added benefits of new revenue streams such as waste tipping fees, as well as reducing nuisance and liability issues, including pathogens, odour, and waste to be handled [235]. Conversely, Pilloni and Abu Hamed [236] noted that over fifty (50) years ago, government and non-governmental institutions encouraged the implementation of small biogas digesters in rural areas, largely in Asia, South America, and Africa. These developing nations are exposed to technical, economic, and policy-related challenges in adopting anaerobic digestion technology. Precisely, in African countries, insufficient funding, technology, and policy frameworks, further complicated by inadequate infrastructure, are the obstacles to the adoption of the said technology. Likewise, Roopnarain and Adeleke [237] registered that the lack of systematic support hindered the project sustainability of the biogas programmes initiated by the governments in Kenya and Uganda.

Based on a systematic review, Ibarra-Esparza and colleagues [226] analysed anaerobic digestion technology and its application in both developing and developed countries and noted that there is a large difference in the maturity level of AD systems between these countries, primarily due to the economic capacity of developed countries to invest in sustainable waste management systems. Over several decades, AD has been in use in developed countries, while its application is still at the initial stages of technological preparedness in developing countries [238]. The implementation of AD as a WM strategy and a waste-to-energy recovery technology has led to the development of AD plants with differing operating capacities worldwide. Italy, France, Germany, and the UK are leading in this field of AD technology with a high number of AD plants [239]. Developed countries tend to have large-scale AD systems operating as two-stage systems under thermophilic conditions because costs can be covered through economic resources to invest in all the needed elements [240], whereas developing countries have more small-scale AD systems installed at small farms and households, operating as single-stage systems under mesophilic conditions based on the tropical climates in these regions or areas [241]. Moreover, the United States Environmental Protection Agency [242] explained that there are strategies, policies, and priority actions involved in transforming to a waste-to-energy technology like AD, which requires higher investment or high cost reflected in terms of pretreatment processes and land considerations alongside mature technical development. Such higher investments and mature technical development are not feasible in many developing countries. In addition, Ibarra-Esparza et al. [226] noted that capital and operational expenditures involved in AD systems exist as the major challenges affecting their implementation level in both developed and developing countries. The application of AD systems entails investment in technology, and, apart from the biodigester itself, investment based on plant design, construction, monitoring systems, and power-generating technology is required [243]. On the other hand, AD in developed countries has attained maturity as it is fuelled by several factors. For instance, the rate of moving waste to AD in the European Union has increased because of a strong economic investment in the biomass and waste-to-energy sectors, coupled with the increased development and updating of policies concerning biogas plants and agreements, which provide countries with a greater incentive to invest in the renewable energy sector [244]. Undisputably, Ibarra-Esparza and colleagues [226], in their findings, delineate the difference in terms of technological maturity of AD between the developing and the developed countries by analysing both reviews and theoretical studies. It was realised that studies in the former were performed aiming at producing data that could serve as a baseline for the development of new policies to encourage sustainability through exploring the energy potential of AD of the organic fraction of MSW generated in certain areas. The performance of AD as a waste-to-energy technology in terms of economic and environmental benefits was evaluated and presented as the focus of the studies carried out in developed countries.

Additionally, the analysis of capital and operational costs of AD plants showed that costs tend to be higher for developing countries due to their need to import materials and equipment from developed countries. Technical, economic, and political challenges for the implementation of AD at a large scale in developing countries are centred on the lack of education about the adverse effects of climate change and clean energy initiatives [245], unacceptability by the communities [246], and lower environmental efforts between the public and private sectors [247]. Similarly, Almansa et al. [171] identified experience, economics, knowledge/training of personnel and users, and stakeholder analysis as challenges facing the utilisation of AD in developing countries. Even though Huang [143] cited bio-electrochemical methanation and membrane bioreactors as advanced technologies to augment biogas production efficiency, alongside co-digestion addressing challenging scenarios, high costs associated with investment, technical inefficiencies (technological gaps related to scalability), regulatory barriers, and extended payback periods are the critical challenges, particularly identified in developing countries. Huang [143] concluded that enhanced technical training, increased public awareness, improved policy support, and strengthened international collaboration and financial assistance are the steps required to address the challenges outlined in these developing regions.

With respect to socioeconomic benefits, the biogas produced through this process is used differently in both developed and developing countries; biogas is mostly employed in electrical power generation, utilised in stoves and lamps, and in thermal power generation in combined heat and power engines in developing countries, but mainly in the generation of commercial electricity in developed countries [248]. Biogas has a high calorific value, which can be exploited for power generation, allowing plants to accomplish energy self-sufficiency [249]. In addition, the integration of cogeneration technology enables facilities not only to satisfy their own heating requirements but also to supply thermal energy to surrounding areas, further augmenting economic benefits [145]. Varied uses are expressed for the digestate, ranging from use as a soil conditioner and forest recovery to disposing of the digestate in soil [192,250] in developing countries. On the other hand, developed countries aim at recovering VFAs, biomethane, biogas, and digestate as their key products via AD of organic waste, thus creating an important opportunity for diversification. The digestate obtained is composted for stabilisation, rendering it more suitable for applications as a fertiliser. Also, Le Pera et al. [251] mentioned the Italian full-scale AD plant located in Rende, which upgrades the biogas producing biomethane (CH4 97–99%), a gaseous or liquid fuel associated with numerous potential applications, including transportation. Other authors, Rossi et al. [82] and Valentino et al. [252], commented that the pilot-scale plants located in Florence and Treviso are focused on enhancing VFA production; a stream rich in VFAs can have valuable uses in the chemical industry as a starting substrate for alcohols, ketones, and esters.

### 3.2. Sanitation and Hygiene (Waste Management)

Presently, there is a continuous requirement to increase food production because of the ever-rising human population. Thus, provoking the application and adoption of new techniques and approaches in agriculture and animal husbandry [253]. Globally, there is an increase in total meat consumption [254] through animal farming. Animal farming is considered one of the oldest, fastest-growing economic sectors in Africa and one of the most traditional industries in the world [255]. Urbanisation, alongside a rising population and disposable incomes, provokes a higher demand for animal-derived food on the continent, resulting in increased domestic livestock production [256]. The rising demand of the population for meat and its derived products leads to the corresponding increase in livestock products and huge quantities of animal manure on a sustainable basis. Animal manure consists of organic matter, nutrients (phosphorus and nitrogen), micronutrients, antibiotics, and heavy metals, and is a renewable resource that is low-cost, largely available, and easily accessible. Sustainability in the production of meat and its derivatives is achieved via the routine application of huge quantities of antibiotics in animal farming to replace costly hygiene and sanitation measures, as well as to increase productivity [257].

However, animal manure may contain varying degrees of microbial (microorganisms and their antibiotic-resistant counterparts, antibiotic-resistance genes) and chemical contaminants (antibiotics), being influenced by livestock practices, which tend to vary from one individual to the next. On the other hand, the pathogenic bacteria identified in animal manure included *Salmonella*, *Shigella*, *Campylobacter*, *Yersinia*, and *Listeria* species, which are true pathogens causing gastrointestinal tract diseases (diarrhoea) in humans. Clearly, Abat and colleagues [258] narrated that key human pathogens are frequently isolated from meat-producing animals, thus making the bacterial infections in humans originating from animal-producing animals an increased global burden. Globally, diarrhoea is ranked second as the common cause of death and morbidity among children under the age of five (5). This suggests that infants are particularly susceptible to foodborne infections because their immune and metabolic systems are underdeveloped, resulting in more severe symptoms in this age group [259].

Some developing countries, especially sub-Saharan Africa (including Central, Eastern, Southern, and Western African countries), are faced with a high burden of infectious and communicable diseases (diarrhoea disease) plus antibiotic resistance, which greatly impacts public health, causing high mortality in low- and middle-income countries [260]. McCord et al. [68] emphasised that urban areas in Kampala, Uganda, carry out livestock farming, yet the residents have little capacity for the management of soil and animal waste. Consequently, the population inhabiting these urban areas is exposed to an increased risk of waterborne, foodborne, and vector-borne diseases. Also, it has been highlighted that HIV-positive individuals diagnosed with enteric fever are more vulnerable to developing enteric fever with frequent relapses [66]. Furthermore, Gaffan and colleagues [261] explained that access to water, sanitation, and hygiene is inadequate in sub-Saharan Africa, and the burden of diarrhoea is immeasurable relative to the rest of the world. The health of the population is greatly influenced by the food they consume and the environment in which they live. Boutayeb [262] remarked that the diseases occurring in these countries can exert a negative impact on education, income, life expectancy, and other health indicators, which impede human development. Sanitation and public health can be affected by improperly managed animal manure [219]. Through a joint monitoring programme, WHO and UNICEF [263] highlighted that access to appropriate sanitation facilities is a major challenge globally, with over one-third of the world’s population still lacking improved sanitation.

Some countries are water scarce, especially developing countries, e.g., South Africa, and manure is managed traditionally via direct application of the raw/untreated manure on farmlands by small-scale farmers in rural communities. In some scenarios, there is a lack of agricultural land for the spread of the manure. Over-application of manure to soil can lead to the accumulation of excessive nutrients in the soil with a great likelihood of polluting the surface and groundwater [264]. Other traditional methods include landfilling, lagoons and storage in tanks. These methods are associated with several environmental and public health challenges because, through hydrological processes including storms, rainfall, winds etc., the waste may eventually end up in water bodies and soil [204,225]. The presence or occurrence of manure may equally affect air quality as well as animals and humans. More elaborately, the adverse effects of poorly managed animal manure include aesthetic nuisance, uncontrolled methane emissions, and several waterborne and foodborne diseases [144].

Table 4 shows different microorganisms found in animal manure alongside the diseases caused in humans and animals. Most of these bacterial species cause food-borne diseases. Foodborne diseases represent an important public health concern, causing a considerable economic burden on both low- and high-income settings [265]. However, according to the World Health Organisation [266], the African region is facing the highest incidence and death rates because of these foodborne diseases. Nevertheless, owing to inadequate detection methods and surveillance systems in some regions of the world, several cases of foodborne disease go unreported [267]. Notwithstanding, among the pathogenic microorganisms, very close attention should be directed to the zoonotic pathogens, because these pathogens cause zoonotic diseases that can be transmitted between animals and humans, causing threats to the health of humans and animals [268]. The nutrients originating from animal manure but found in water bodies may cause eutrophication [269], leading to oxygen depletion and death of aquatic life, alteration in the water microbiomes and quality; this eventually leads to reduced productivity and economic losses faced via the cost incurred for treating contaminated water or bioremediation.

Overall, in low- and middle-income countries, the essential sanitation technologies across the sanitation chain involve the user interface (toilet), onsite storage, transport, treatment and disposal of waste, and recovery and reuse of resources [171]. Although all these links are important, the accessibility of waste treatment and resource recovery technologies tailored to specific communities’ needs appears to be the key component. Of significance is the implementation of a suitable solid waste management system with the potential to deal with high production of biowaste on the one hand and the increased need for water and energy resources on the other [270]. Anaerobic digestion offers a unique avenue that combines on-site waste treatment, pathogen inactivation, and resource recovery, making it effective and desirable for small-scale applications. Therefore, distributed anaerobic systems (decentralised), along with subsequent resource recovery technologies, can have a huge impact on the sanitation in underserved communities. In this light, decentralised anaerobic systems can be developed for individual households or for communities, with the decision influenced by the local conditions and constraints. At the level of the community, decentralised anaerobic systems are exposed to challenges that are described as context-specific, leading to technology failure and abandonment. These challenges are categorised into three major areas: economic challenges, the need for training, and associating stakeholder needs and preferences with potential products of anaerobic digestion substantiated to include limited resources available for commercialisation of products, unfavourable environmental conditions (e.g., low temperature), restricted space for installation, variable availability and seasonality of waste, and limited knowledge/training and engagement of stakeholders and users [171].

In addition, plant-based waste or lignocellulosic waste, including corn/wheat straws, corn cobs, sawdust, and others, are disposed of by landfilling, composting, and combustion, which are equally faced with soil, water, and air pollution effects [62,76]. To avert or mitigate the aforementioned deleterious effects of poor management of animal waste, a few techniques ranging from simple, low-cost, and highly complex strategies are necessary for the proper handling of these wastes. In addition, the enactment of relevant legislation, regulations, standards, and policies for monitoring manure management practices and ensuring compliance with environmental standards, alongside protecting public health, are available to promote sustainable handling of animal manure [269]. Some of these are enacted at the community, state, national, regional, and international levels. Treatment of animal waste via composting, anaerobic digestion, vermicomposting, and others, alongside animal-inclusive water, sanitation, and hygiene (A-WASH), are among the techniques described for the proper management of animal wastes [62,206,256].

**Table 4:** Selected microbial population in animal manure and associated infections caused in humans and animals.

Microorganisms	Description of the Organism	Infections Caused in Humans/Animals and Symptoms	Route of Infection	References
*Escherichia coli*	It is a Gram-negative bacterium that occurs as a commensal among the first, colonising the gut following birth. *E. coli* can be differentiated into phylogenetic lineages that are distinct, such as A, B1, B2, D1, D2, E, and Clade I. Accordingly, *E. coli*, through genetic changes based on the high plasticity of its genome, evolves producing different pathotypes different from their commensal counterparts, which are associated with different disease conditions. These pathotypes include EPEC(Enteropathogenic), EHEC (Enterohemorrhagic), ETEC (Enterotoxigenic), EIEC (Enteroinvasive), UPEC (Uropathogenic), APEC (Avian pathogenic), *E. coli*, etc.	The diseases are presented according to the different pathotypes described in the previous column, including acute and prolonged diarrhoea in children, haemorrhagic colitis and haemolytic uraemic syndrome, traveller’s diarrhoae, dysentery and watery diarrhoea, urinary tract infections, septicaemia, and severe respiratory and systemic infections in poultry.	Contaminated food and water	Mudau et al. [271]; Pokharel et al. [272].
*Salmonella* spp.	Gram-negative, non-spore-forming, aerobic, and facultative bacteria belonging to the family Enterobacteriacea. Pathogens with the greatest probability of being spread in the environment through animal slurry and sewage sludge. All serotypes are harmful to both humans and animals. Of increasing global public health concern to both animals and humans relating to antimicrobial resistance.	Food poisoning/gastroenteritis, which is characterised by fever, diarrhoae and abdominal cramps. Also, more severe systemic diseases, including typhoid fever.	Contaminated food and water	Billah and Rahman [273] and Lamichhane et al. [274].
*Campylobacter jejuni* *Campylobacter coli*	The genus *Campylobacter* belongs to the family Campylobacteriaceae. These two species are closely related by phylogenetic and genetic measures. The species can be differentiated using high-resolution melting curve analysis targeting variations in the sequence of the *cad*F gene. This gene mediates cell binding to the cell–matrix protein, Fibronectin. They are both incriminated in human gastroenteritis. Accordingly, antibiotic treatment is based on the species causing the infection (*C. jejuni* with Erythromycin, while *C*. *coli* is resistant to this drug).	Bacterial diarrhoael illness (traveller’s diarrhoae, which can be self-limiting), bacteraemia, abscess, and meningitis.	Consumption of contaminated food or water, direct contact with faeces of infected humans or animals	Zenebe et al. [275];
*Listeria monocytogenes*	It is a well-known Gram-positive, facultatively anaerobic, non-encapsulated, rod-shaped intracellular bacterium that is ubiquitous in nature, i.e., found everywhere. It belongs to the genus *Listeria*, family Listeriaceae, classified into four (4) major lineages 1 to IV, consisting of 14 serotypes. *L. monocytogenes* demonstrates great strain divergence coupled with increased antibiotic resistance as well as biofilm potential.	The bacterium causes listeriosis, which can occur as non-invasive, self-limiting gastroenteritis and invasive listeriosis. Clinically- associated manifestations such as encephalitis, pneumonia, meningitis, septicaemia, sepsis, brain infection, abortions or stillbirths, headache, backache, etc.	Contaminated ready-to-eat foods.	Getaneh et al. [276]; Manyi-Loh and Lues [277].
*Yersinia enterocolitica*	*Y. entrocolitica* is a species belonging to the genus *Yersinia* and family Yersiniaceae. It is a very important zoonotic pathogen, whose reservoir is pigs. *Y. enterocolitica* is considered the third bacterial cause of gastrointestinal infection in humans, residing in Europe, whilst its infection in West Africa is rare. Its pathogenicity relies on the expression of chromosome and plasmid determinants.	It causes yersiniosis in both humans and animals. Clinical manifestations include enteritis, colitis, secretory and bloody diarrhoae, fever, stomach pain, and vomiting. Yersiniosis is associated with significant economic effects relating to health and veterinary costs, trade restrictions, productivity and livestock losses, food recalls, and the need for compliance and public health measures.	It is foodborne and transmitted through contaminated meat, milk, and related products. Infection occurs through sick or asymptomatic carriers and contact with the faeces of infected animals.	Lemos et al. [278]; Sohoty et al. [279]; Grygiel-Górniak [280].
*Staphylococcus aureus*	It is a Gram-positive coccoid-shaped opportunistic bacterium, registered among the significant foodborne pathogens. The staphylococcal enterotoxins, a class of heat-stable enterotoxins with the ability to induce superantigen activity, lead to immunosuppression and T-cell proliferation. The enterotoxins demonstrate resistance to protein hydrolytic enzymes and low pHs, allowing them to maintain their activity in the gastrointestinal tract following ingestion. The biofilm ability of this bacterium threatens and exacerbates infections by permitting its attachment to pathological areas and livestock product surfaces.	In humans, the bacterium causes primary bacteraemia, otitis media, soft tissue infection, pneumonia, and septic arthritis. In animals, it causes mastitis, joint infections, skin infections, and bacterial chondronecrosis with osteomyelitis.	Foodborne, direct contact with skin.	Zhou et al. [281]; Song et al. [282].
*Shigella dysenteriae*	It is a Gram-negative, facultatively anaerobic, non-motile, non-spore-forming, non-lactose-fermenting, rod-shaped bacterium, which exists in humans as the only host. It produces Shiga toxin, and it is genetically highly correlated with *Escherichia coli.*	Shigellosis (bacterial dysentery) or bacillary dysentery in humans. It is an acute inflammation of the intestines, presenting as mild watery diarrhoea to severe inflammatory bacillary dysentery. In severe cases, haemolytic uraemic syndrome, kidney failure, and even death. Symptoms include fever, abdominal cramps, mucus and blood in the stool, and vomiting.	The faecal–oral route, contaminated food or water, or direct contact with infected persons (food handlers), and poor hygiene.	Ayele et al. [283]; Hmar et al. [284].
*Cryptosporidium parvum*	It is a protozoan parasite belonging to the genus *Cryptosporidium*, class Coccidia, and phylum Apicomplexa that occurs as a parasite in both humans and animals, exhibiting a monoxen cycle (i.e., completing its life cycle within a single host), with alternating asexual and sexual reproduction. Together with *C. hominis*, they occur as the only species of the genus *Cryptosporidium* that infect humans.	It causes cryptosporidiosis, a water-and food-borne zoonotic disease, with no available therapeutic drugs or vaccines that are effective for treatment and control. Associated symptoms include vomiting, diarrhoea, nausea, abdominal pain, and fever.	Faecal–oral route transmission by ingestion of viable oocysts deposited in the faeces of infected humans and animals, contaminating food or water, and is added to the respiratory route.	Helmy and Hafez, [285]; Enbom et al. [286].
*Vibrio cholerae*	It is described as a curved, rod-shaped, motile Gram-negative, zoonotic bacterium that inhabits aquatic environments. It is a major public health concern, especially in countries with poor sanitary conditions and areas affected by natural disasters. *V. cholerae* is a species of bacteria with a remarkable capacity to adapt and evolve, making it a great global concern as it raises the risk of cholera outbreaks and its distribution to new regions, thereby rendering control of the disease challenging.	It is the causative agent of cholera, via secreting an AB5 multimeric toxin, cholera toxin (CT), that binds directly to intestinal epithelial cells. Cholera is a severe and highly contagious diarrhoeal disease that can cause extreme dehydration, leading to cholera-associated deaths.	The faecal–oral route of transmission or indirectly through contaminated food and water or person-to-person contact.	Dominguez et al. [287], Montero et al. [288].
Rotavirus	It is a highly infectious virus associated with the most common cause of diarrheal-related deaths and the fifth (5th) highest cause of death in children under five (5) years. The virus demonstrates genetic heterogeneity across different strains, and it can infect a wide range of species, suggesting that it possesses the potential to produce highly virulent variants via gene reassortment.	Infections caused by Rotavirus are the leading cause of severe dehydrating gastroenteritis in children under the age of five (5).	Transmitted via the faecal–oral route in humans. In animals, the possibility of transmission is via ingestion of contaminated feed or water, exposure to contaminated environments, and contact with infected animals.	Crawford et al. [289]; Njifon et al. [290].
Avian influenza virus	It is described as a negative-sense single-stranded RNA virus with eight gene segments comprising haemagglutinin, neuraminidase glycoproteins, and six (6) internal genes. Avian influenza viruses are also known as bird flu or avian flu. These viruses are classified as a type of influenza virus. The evolution of Avian influenza A (through H_1_N_1_, H_2_N_2_, H_3_N_2_, H_5_N_1_) is a crucial driver for the emergence of pandemic strains. On a global scale, these viruses are challenging owing to widespread circulation and high mortality rates. Outbreaks of this virus affect all age groups, displaying distinct geographical epidemiological patterns of its infections in humans across regions, provinces, and countries.	Infections ranging from mild to severe, presenting with symptoms including fever, cough, sore throat, pneumonia, and acute respiratory distress syndrome.	Human transmission occurs via direct contact with infected birds, human-to- human transmission. Inter-premise airborne transmission and through virus-contaminated surfaces and materials.	Charostad et al. [291]; Kang et al. [292].
*Clostridium* spp.	Gram-positive, anaerobic spore-forming bacteria, with known pathogen species, including *C*. *perfringens* and *C*. *botulinum*, that produce toxins. Also, *C*. *difficile* is of high prevalence in animals. Presently, diseases caused by these bacterial species are persistent because of challenging diagnosis, vaccines and treatments are not readily available, and these bacterial species occupy habitats everywhere in the environment (ubiquitous).	Clostridial diseases are grouped into enteric, neurotoxic, and histotoxic. Human and animal botulism, gangrene, necrosis, pseudo-membranous enteritis, food poisoning, enterocolitis, and enterotoxemia. Symptoms include muscle weakness, vomiting, nausea, headache, respiratory failure, dizziness, severe abdominal pain, and diarrhoea.	Foodborne, i.e., ingestion of contaminated food, spores. Direct contact with animals (contaminated wounds) and indirect transmission via the environment.	Uzal et al. [293]; Wang et al. [196].
*Bacillus cereus*	Gram-positive, facultatively anaerobic, aerobic, endospore-forming, ubiquitously distributed, and opportunistic bacterium. During vegetative growth, the bacterium pre-forms an emetic toxin known as cereulide (a small peptide of molecular weight 1.2 kDa, which is responsible for the emetic syndrome). Cereulide is one of the important determinants of the pathogenicity of *B. cereus*. In addition, the emetic toxin is a cyclic dodecadepsipeptide, highly stable towards heat, acid, and digestive enzymes; therefore, it can be difficult to remove or become inactivated. This is because it comprises a repeated sequence of [D-O-Leu D-Ala L-O-Val D-Val]3. *Bacillus* spp. are regarded as safe bacteria endowed with remarkable abilities for encouraging plant growth.	It causes both local and systemic infections in humans. In other words, it causes gastrointestinal and food poisoning in addition to gastrointestinal infections (rare). It causes two types of gastrointestinal symptoms, the emetic type, which appears half an hour following ingestion of food intoxicated with cereulide, the emetic toxin. This is associated with nausea and vomiting. Secondly, the diarrhoeal form of food poisoning manifests as diarrhoea and abdominal cramps. The emetic syndrome is clinically indistinguishable from intoxication with enterotoxins of *Staphylococcus aureus*. In animals, it causes anthrax-like disease, manifesting as septicaemia and sudden death.	In humans, transmission of infections is via the ingestion of contaminated foods. Spore production and environmental transmission. Precisely, through cutaneous, inhalation, or gastrointestinal routes.	Dietrich et al. [294]; Calvigioni et al. [295]; Jiranantasak et al. [296].
*Enterococcus faecalis*	It is described as a Gram-positive, facultatively anaerobic, and opportunistic/nosocomial bacterium with the ability to resist or withstand harsh environmental conditions. Therefore, it demonstrates great adaptability. The bacterial species belonging to the genus *Enterococcus*, family Enterococcaceae, phylum Firmicutes, is also known as faecal streptococci. It is suggested as the most suitable bacterium to validate the hygienic treatment of biowaste in biogas plants. The bacterium produces proteins as virulence factors that enhance its pathogenicity. More attention is given to *E. faecalis* because of its extensive resistance to multiple antibiotics through plasmid and transposon transfer, chromosomal exchange, or mutations. Based on its ubiquitous nature, outstanding adaptative capability added to the propensity to acquire virulence and resistance genes, making them excellent sentinels for evaluating the spread or presence of the presence/spread of pathogenic and virulent clones and hazardous determinants across settings of the human–animal–environment triad.	It has graduated from being a commensal bacterium to becoming a leading pathogen in humans and animals. In humans, it causes urinary tract infections. In animals, it leads to endocarditis, diarrhoeae, septicaemia, and mastitis.	Transmission via handling and consuming contaminated foods or direct contact with animals or their environment.	Marques et al. [297]; Mubarak et al. [298]; Cebeci [299].

#### 3.2.1. Antibiotics and Antibiotic Resistance

Antimicrobial substances or agents are employed in huge quantities to sustain production in animal farming because they are used for prophylaxis and therapy of infectious diseases alongside growth promotion [300]. It is remarkable to mention that several antimicrobials employed in animal agriculture (veterinary medicine) are the same or surrogates of those used in human medicine, thus attracting attention [74]. Owing to the poor nature of absorption of antibiotics by the animal’s gut, animals excrete significant amounts of unmodified or still-active forms of antibiotics into the environment. Under such environmental conditions in the presence of antibiotic concentration, the microorganisms tend to adapt to the prevailing conditions to ensure their survivability [301]. Over time, microorganisms in the presence of relatively high concentrations of residual antibiotics develop resistance to combating these antibiotics [302] through long-term adaptation, resulting in the advent of bacterial species with antibiotic-resistant capabilities that would proliferate in the environment.

The indiscriminate and imprudent use of these antimicrobial substances has steered the quick evolution of antibiotic-resistant bacteria and antibiotic-resistance genes, decreasing the potential of these drugs for therapeutic functions or purposes against animal and human pathogens [303]. In addition, the occurrence of antibiotics in the environment may create a selective advantage for antibiotic-resistant bacteria, causing their propagation and spread in the environment [304]. The extensive use of antibiotics in both livestock and poultry production systems elevated the detection of antibiotic-resistant genes in livestock and poultry manures, which may be transferred to other indigenous bacteria in the environment via horizontal gene transfer [305]. Therefore, creating a pool of antibiotic-resistant bacteria and antibiotic-resistance genes presents health threats to animals and the human population [74].

#### 3.2.2. Inactivation/Reduction in Bacterial Pathogens

The substrates employed in anaerobic digestion may be contaminated with diverse categories of microorganisms, including viruses, bacteria, fungi, and parasites of veterinary and public health concern [306]. Accordingly, McDaniel et al. [307] noted that approximately 150 species of human and animal pathogens were identified in animal manure. These microorganisms can be described as disease-causing and non-disease-causing pathogens [146], with the potential to survive in the soil, water, and other environments for a long duration [74]. Subsequently, the microorganisms can be disseminated to humans and animals directly by contact or indirectly via contamination of food and water. They can equally be distributed through uncontrolled application of animal manure on land as fertiliser or during the processing of meat and milk [205]. Pathogen destruction and control can be performed via physical, chemical, and biological methods in addition to a combination of these. Manure occurs as a potent source of noxious and greenhouse gases that will end up in the atmosphere and environment during slatted storage and land spreading [308]. Composting and anaerobic digestion are presented as the two most common methods employed in the hygienisation of biowastes [146]. However, composting pays little attention to the loss of gaseous nitrogen (N) or methane (CH_4_) to the environment [309]. Moreover, other methods, such as acidification during storage, which target the reduction in ammonia or other greenhouse gases (GHG) from manure during the said treatment, rarely consider the fate of the pathogens [310].

Anaerobic digestion serves as a technological solution that holds great promise for the treatment of manure, recovering energy, mitigating noxious and GHG, as well as reducing the potential load of pathogens in the environment and the related risks of public health concerns [311]. AD (a biological process) results in the inactivation of bacterial pathogens based on a blend of parameters, including the initial microbial load, type of substrates, intermediate products, competition between microbes, pH, temperature, and ammonia production [311]. In detail, factors affecting the inactivation rate of pathogens during anaerobic digestion can be categorised into dependent and independent factors. Independent factors include temperature, hydraulic retention time (HRT), carbon-to-nitrogen ratio (C: N), solid retention time (SRT), and organic loading rate (OLR), while dependent factors, including pH and toxicity development caused by excessive free ammonia, can be monitored during the process of anaerobic digestion [312].

The recommended temperature at which anaerobic digestion of feedstock materials is performed occurs in the mesophilic range (25–45 °C) as opposed to thermophilic and psychrophilic temperature regimens, as it evades the need for additional heating supply systems, incurring cost associated with thermophilic treatment added to the instability that takes place during treatment at colder temperatures [120]. Pathogenic microorganisms that survived the anaerobic digestion process at mesophilic temperatures could possibly be disseminated in digestate (discharged effluent from biodigester) and spread as fertiliser on farmland. Thus, the pathogens may contaminate a host of environmental milieus, posing a potential threat to the health of the population [313]. Apparently, through food, water, and the air pathways, humans become exposed to these pathogens. In particular, the targeted pathogens can be transmitted through the food chain to humans, especially with ready-to-eat crops that may be eaten without further treatment or processing [149]. Therefore, this category of crops serves as a potential and crucial vector [312]. In this regard, varied findings conducted by several researchers pointed out the potential of anaerobic digestion in the inactivation of common bacterial pathogens found in animal manure [149]. This inactivation results in the destruction of the pathogens, which is expressed as reduced prevalence rates of the microorganisms. Table 5 shows different levels of inactivation of common bacterial pathogens occurring in animal manure via anaerobic mono-digestion or co-digestion.

Table 5 harbours survival data for selected bacterial pathogens found in animal manure, as it was subjected to anaerobic digestion. The data highlight the pathogen reduction capacity of anaerobic digestion, which is commonly termed hygienisation [53]. Although different measurements were obtained for the different studies based on the tested parameters, the data provide a good indication of the relative resistance of each bacterial pathogen in the substrates as observed under varying tested conditions [314]. The survival rates observed in the different studies vary because most of the studies on the inactivation of the pathogens were performed at the laboratory scale, but with few studies conducted at full scale. Even the studies performed at the full scale involved one or two digesters and focused on either one or a few bacterial species [149]. Therefore, owing to the lack of standardisation of the anaerobic digestion process and the varying pathogens found in the biowastes, case-by-case evaluation becomes crucial. The varying inactivation rates of the different bacterial pathogens are attributed to process efficacy [315], highlighting anaerobic digestion as an effective technique for animal manure treatment prior to their safe disposal and utilisation [206]. This is because Burch and colleagues [149] detected lower frequencies of virtually all microbes in the digestate, or effluent discharged, than in the influent material fed into the digester. The process creates an excellent opportunity to alleviate the biosafety of biowaste, alongside decreasing human exposure to foodborne-related pathogens [315].

As can be viewed in Table 5, the variation in the activation rates can be ascribed to different study methodology (culture-based or molecular methods for quantification), biodigester type (batch or continuous or semi-continuous operation), digestion temperature (mesophilic or thermophilic), substrate type (single or multiple), total solid concentration, pH, organic loading rate (OLR), hydraulic retention time (HRT), feedstock composition (in terms of initial load/concentration of specific microbes and the type of microorganisms) [203], expertise of the researcher, pretreatment method (storage or no storage, chemical, physical, biological treatments), digester design, and the region/country of study [146,179,204]. Burch et al. [149] stated that overloading, poor mixing, and poor temperature control are other reasons for slower rates of microbial inactivation. The authors further explained that overloading and poor mixing reduce effective residence time and permit the formation of dead zones in digesters, respectively, therefore providing less time to inactivate pathogens. Poor temperature control creates less than optimum values, and this level of temperature causes lower rates of microbial inactivation. Arthurson [316] studied proper sanitisation of sewage sludge and acknowledged that, in as much as heat could cause rapid pathogen inactivation, the combination of high temperature and short time (>7 min at 70 °C) or long time and low temperatures (>3 days at 55 °C) was found effective in reducing the pathogen in waste treated under laboratory conditions. Thus, emphasising that inactivation due to temperature is related to time [317].

In addition, Lin and colleagues [146] demonstrated that high temperatures, high volatile fatty acids, and ammonia concentration alongside long retention time exerted positive effects on pathogen inactivation during anaerobic digestion of biowaste. Salsali et al. [207] claimed that most pathogens survived for a longer duration when mesophilic treatment temperatures (30–42 °C) were employed as opposed to thermophilic temperatures (50–55 °C). Apparently, Manyi-Loh and Lues [204] affirmed that the inactivation of bacteria is temperature-dependent, so too, the activities performed by anaerobic microbes during anaerobic digestion are temperature-dependent. Similarly, Chukwu and colleagues [76] purported that the different temperature regimen processes (psychrophilic, mesophilic, and thermophilic anaerobic processes) exert varying impacts on pathogenic microorganisms, with the different microbes being reduced by different amounts through each process, especially as different organic wastes from diverse sources harbour different concentrations of these microbial pathogens. In this light, Akindolire and colleagues [120] explained that the anaerobic microbes secrete enzymes that are responsible for the degradation process, especially during the hydrolysis phase. The authors explained that a change in the temperature (from 37 to 70 °C) provokes increased fluidity and permeability of the cell membrane, allowing more rapid diffusion of toxic chemicals into the cytoplasm, thus leading to the inhibition of cell growth. In addition, Orzi and co-authors [311] opined that the effect of temperature can be directly acting on the pathogen or indirectly by heightening the impact of other chemicals, for example, volatile fatty acid toxicity and ammonia. Lin et al. [146] mentioned that bacterial inactivation due to temperature is related to time, but the retention time works together with other factors to inactivate the pathogens. Thus, suggesting that it is the combined effect of the above-mentioned factors that can cause the inactivation of the microbial pathogens.

As noted previously by Hofmann and colleagues [318], laboratory-scale systems may not be a true representation of full-scale continuous commercial bioreactors because of the disparities in the inoculation methods, rheology, and hydrodynamic methods. In addition, Burch and co-authors [149] remarked that full-scale studies usually investigate one or two digesters, which could not characterise variation in pathogen inactivation that would be expected to occur due to differences in operations on the different farms. Digesters (design and types) vary considerably in their capacity to reduce pathogenic microbes as they determine the organic loading rate as well as the hydraulic retention time [68]. The digester design and operational parameters strongly affect the performance of the system, and one of such parameters is operational temperature, which can fluctuate owing to changes in atmospheric, ambient, or environmental temperature. This is so because some digesters are more sensitive than others to the changing ambient temperature based on variation in digester depths as well as their degree of insulation.

Considering that thermophilic and higher mesophilic temperature treatments lead to the removal or inactivation of bacterial pathogens, a solar-powered heating element could be suggested to increase the temperature inside the digesters. However, the possible interventions are either cost-prohibitive or not available, depending on the region or country. Most studies on pathogen reduction were performed using a batch-operated digester, where the hydraulic retention is tightly controlled and, following the feeding of the digester, the feedstock is retained until the end of the process. Subsequently, the effluent is discharged. With these studies, it is obvious that longer retention times are more likely to result in greater pathogen elimination. On the other hand, continuous systems are associated with retention times that are changing in response to the feeding regimes. Herein, there is intermittent loading of the digester with feedstock and corresponding discharge of effluent from the digester; thus, undigested wastes may pass through the digester in several days. A situation that is very critical is when digestate must be employed immediately for land application, as the microbial load can possibly be very high.

The physiology, morphology, and metabolism of microorganisms affect their survival under different stress conditions. Therefore, diverse behaviours are exhibited by the different microbial groups (Gram-positive bacteria, Gram-negative bacteria, Gram-positive spore-formers, viruses, or parasites) during anaerobic digestion, risking the extrapolation of results from one group to others [53]. However, it is challenging, time-consuming, and impossible in practical scenarios to detect all the potential pathogens that occur in animal wastes or digestates [319]. In this light, the choice of pathogen indicators is crucial for the evaluation of effective quality/safety. To monitor key microbial groups in animal waste or digestates, the EU regulation incorporates specific indicators, including *Escherichia coli* (Gram-negative bacteria), *Enterococcus* spp. (Gram-positive bacteria) and *Clostridium perfringens* (Gram-positive spore-forming bacteria).

As shown in Table 5, *E. coli* and *Salmonella* spp. are highly studied pathogens as they appeared in all the studies performed in different countries because they are described as indicator organisms to evaluate environmental contamination. These indicator organisms, occurring in large numbers in the intestinal tracts of healthy and unhealthy animals and humans (though not necessarily pathogens), are easily detectable and countable. Therefore, they are very important for detecting and estimating the level of faecal contamination of water. The presence of faecal pathogens indicates the effect or impact of hygienic treatment of biowaste [76,146]. Correspondingly, Manyi-Loh et al. [320] explained the ordeal of having these pathogens in high concentrations, indicating potential threats to health owing to water and food contamination with bacterial pathogens of animal origin (zoonotic). Therefore, Motlagh and Yang [321] narrated that these pathogens, serving as indicators, provide a simple, standardised approach to evaluate the efficacy of the different treatment processes.

Notwithstanding, other pathogens were studied, including *Listeria monocytogenes* and *Enterococcus faecalis*, among others. Overall, these bacterial pathogens can be grouped into Gram-positive and Gram-negative organisms based on their cell wall composition, which has the potential to influence the responses of these organisms to anaerobic digestion; this, however, affects the degree of bacterial inactivation. Lin and co-authors [146] pointed out that Gram-negative bacteria possess thin cell walls (i.e., a single layer) that constitute only an outer membrane that is rich in lipids and a monolayer of peptidoglycan, causing their susceptibility to raised temperatures and levels of volatile fatty acids (VFAs); therefore, they demonstrate low resistance against the stress environment [311].

On the other hand, the thick layers of peptidoglycan occurring as multi-layers in the cell wall of Gram-positive bacteria act as a permeability barrier, preventing the penetration of toxic chemicals into the cells [311]. Moreover, even within these two categories of organisms, the organisms differ based on taxonomical hierarchy, including family, order, genus, and species. Therefore, they may demonstrate varying responses to the effect of anaerobic digestion, determined by their nutritional and physiological requirements/characteristics, competition, and antagonism from other microbes that occur within the closed biodigester system. The physiological structures and traits of the various pathogens influence their ability to resist adverse environmental conditions [146]. Therefore, the different bacterial species have different decimation, elimination, decay, or die-off rates [322]. In addition, these microorganisms could be quantified using molecular-based (qPCR) or culture-based techniques (total viable counts or total plate counts), both of which are associated with certain limitations [203]. In this light, real-time polymerase chain reaction (qPCR) is faced with the challenge of distinguishing between live and dead microbial cells. Thus, the magnitude of the estimated cells can be exaggerated, as dead DNA would be available for qPCR amplification in anaerobic digesters. Also, culture-based methods are fraught with the challenge of underestimation of the number of viable or live cells due to the phenomenon of a viable but non-culturable state (VBNC) or active but non-culturable state [323]. A survival strategy for bacteria is that most enter this state to avoid adverse environmental conditions, during which their metabolic activity is greatly reduced, as well as their ability to divide and proliferate, and even their ability to form colonies in the medium becomes lost [146]. Another category of microorganisms that is a challenge to the culture-based methods is the persister cells, which are formed by bacteria in their response to unfavourable conditions, especially antibiotics that are usually employed during animal farming. These cells are the dormant variants of regular cells, which are metabolically less active. The consequence of the resistance exhibited by the bacterial pathogens is obstacles to their effective inactivation.

Seemingly, Oliver [324] emphasised the possibility of the number of culturable bacterial cells declining over time in a specified environment at a much faster rate than viable bacteria. Overall, Ma and colleagues [315] substantiated the varying inactivation rates of <5 log10 and >5 log10 reduction in *Salmonella* spp. in anaerobic digesters operating at ambient, as well as mesophilic and thermophilic temperatures, respectively. The authors further stated that mesophilic and thermophilic AD were principally successful in inactivating bacterial pathogens without pretreatment. Wainaina and colleagues [325] explained that the destruction of bacterial pathogens originated from a rise in temperature and an alteration in pH as the substrates are converted into end products (biogas and digestate) during anaerobic digestion. The inactivation rate expressed in terms of logarithmic reduction in counts of a particular bacterium (log10 cfu/mL or log 10 cfu/g) differed among the studies presented in the table, but these rates occurred over time as the different substrates were treated via the anaerobic digestion process, which progresses through the four phases (i.e., hydrolysis, acidogenesis, acetogenesis, and methanogenesis).

In relation to pathogen inactivation during anaerobic digestion, the efficacy of the process is inconsistent [315], which can be explained by the fact that, for a long time, the primary focus has been on the efficiency of the anaerobic digestion process, producing biogas, while less attention is given to the aspect of controlling pathogens [146]. Therefore, it remains challenging to elucidate optimal pathogen reduction conditions because the experimental studies are limited in their scope, assessing the inactivation of certain pathogens under specified operational conditions [53]. Nonetheless, the logarithmic reductions in the bacterial pathogens harboured in the table indicate a direct reduction in the bacterial pathogenic load (lower contaminants) and the contamination possibility of the digestate, presenting the digestate obtained from anaerobic digestion as a better option of fertiliser in terms of the concentration of enteric bacteria [204] alongside lower greenhouse gas emissions than the direct application of the original or untreated waste or manure [325]. Apparently, the data explain that anaerobic digestion can effectively decrease foodborne pathogens, including faecal coliforms, *E. coli*, *Salmonella* spp., etc. As such, the decrease in bacterial pathogens is indicative of reduced potential pathogen load to the environment and accompanying public health risks [326]. Therefore, the data presented are very crucial because these pathogens have the potential to persist in the soils following the application of treated manure, and there exists a relationship between their presence and the security of agricultural goods [327]. Apparently, the actual risk of using the digestate is very site-specific owing to the unevenness in the prevalence and virulence of the pathogen, susceptibility of the human population (immune status, age, nutritional status, etc.), behavioural risks (use of personal protective equipment, time of application, and other local farm practices, etc.), and microclimatic conditions (humidity, temperature, storage conditions, etc.) [328]. Nevertheless, the use of digestate as fertiliser appears to be of great public health consideration or health as opposed to the discharge of effluent directly into waterways, especially in regions where there are no restrictions on the land application of manure-based solids, human excreta, and wastewater [68].

#### 3.2.3. Reduction in Antibiotic-Resistant Bacteria and Resistance Genes

In a setting where antibiotics are in routine use, such as agricultural settings, the emergence of antibiotic resistance is unavoidable. This phenomenon is natural, and it is an inherent response to the use of antibiotics over a prolonged period due to selective pressure and gene mutations in the bacterial species [76]. Antimicrobial resistance is an issue of the utmost importance on a global scale because of limited production of novel antibiotics, while the rate of antibiotic resistance is rising, along with the infections caused by antibiotic-resistant bacteria being very difficult to treat or untreatable [301]. In addition, even when treatment is possible, the very last-line antibiotics that are expensive and associated with adverse side effects are administered to patients. Moreover, antibiotic resistance escalates healthcare costs, prolongs disease, increases the risk of epidemic outbreaks and the occurrence of complications, reduces the effectiveness of the antibiotics, and increases the risk of failure in treatments and sometimes, death [329,330].

Antimicrobial resistance explains the capacity of microorganisms to survive antimicrobial treatments. Examples of antibiotic-resistant organisms frequently found in livestock that have demonstrated their ability to resist several antibiotics include *Escherichia coli* and *Salmonella* spp. [331]: therefore, making them suitable candidates for researching specific resistance genes as markers for environmental sample detection [330]. The distribution of antimicrobial resistance is encouraged by elevated and uncontrolled use of certain antibiotics. According to the Health Emergency Preparedness and Response Authority [332] and the World Health Organisation [333], antimicrobial resistance is grouped among the top three threats in the European Union and among the ten biggest threats to human health, respectively, making this aspect the focus of global attention [334]. Antimicrobial resistance genes can be spread vertically or horizontally among microorganisms, moving freely among the three One Health major compartments, i.e., humans, animals, and the environment. On several occasions, environmental microorganisms are regarded as the primary hosts of antimicrobial resistance genes that can be disseminated to human pathogens. Therefore, to attain optimal health for humans, animals, and the environment, including plants and soil, the One Health approach becomes vital, consisting of a collaborative effort of multiple disciplines [335].

More seriously, the World Bank estimates that by 2050, there will be 10 million deaths owing to antimicrobial resistance. Thus, it presents a warning to countries to regulate the sale and usage of antibiotics [336]. Limiting the use of antibiotics in the animal-agriculture system through policies is presented as a channel to coerce control of excessive use of antibiotics, but relying on the natural degradation of ARGs transferred to the environment is not realistic over time [337]. Therefore, there is an insistent need for methods that target the removal of residual antibiotics, eradication of antibiotic-resistant bacteria, and elimination of antibiotic-resistance genes from different biological wastes prior to their discharge into the environment [338]. Accordingly, manure management technology, anaerobic digestion, offers a huge potential remedy for the degradation of ARGs in wastes during treatment prior to land application of biofertiliser. This technology is widely used for manure management because of its potential to remove pathogens and ARGs, reducing the volume of the manure along with producing biogas [339]. Furthermore, Wang et al. [340] remarked about the feasibility of an anaerobic digestion approach for the removal of antibiotic-resistance genes. Some findings on the reduction in ARGs during anaerobic digestion of biowaste are presented in Table 6.

As shown in Table 6, tetracyclines, macrolides, sulfonamides, ciprofloxacin, and enrofloxacin antibiotics are the most used antibiotics in animal farming for growth promotion and disease control [350]. Due to the misuse of these antibiotics, antibiotic resistance develops, which consists of a group of microorganisms expressing their ability to grow or survive in the presence of a certain level of antibacterial agent that usually inhibits their growth or other members of the same species [334]. Antibiotic resistance can be described as natural or acquired resistance occurring through selective pressure/genetic mutation or a shift in bacterial genes between chromosomal and extrachromosomal DNA and subsequent transfer to progeny cells via vertical gene transfer (VGT) as well as between bacteria of different species and genera by horizontal gene transfer (HGT) [334]. Accordingly, bacteria develop resistance to these antibiotics as presented in the above-mentioned table.

Therefore, antibiotic resistance genes (ARGs) mediate antibiotic resistance as they are associated with these antibiotics and, as such, were tracked during anaerobic digestion. Naivol et al. [337] explained that the fate of ARGs in anaerobic-based processes can be affected by four main categories of factors, such as substrate characteristics, pretreatments, additives, and operational parameters. The fates and behaviours of these genes expressed as log reductions tend to vary across the different findings as presented in Table 6. In other words, the inactivation of ARGs is affected by factors such as substrate type, the level or concentration of antibiotic residues in substrates, co-digestion ratio, presence of heavy metals, and the type of anaerobic digestion (whether wet or dry), mono- or co-digestion, and thermophilic or mesophilic digestion (temperature) [196]. In other words, Gurmessa et al. [351] stated that the removal of ARGs can be affected by pretreatment, post-treatment, residence time, and temperature. Among the factors, temperature, which is regarded as the basic engineering control, is paramount, and the higher the temperature, the greater the removal of ARGs as opposed to the mesophilic treatment [300]. In this light, Budatala et al. [49], in their findings based on quantitative PCR and metagenomics sequencing, showed that higher temperatures improved ARGs removal, with the greatest substantial reductions observed at 65 °C. The authors described the temperature-dependent activity as correlating with alterations in the structure of microbial communities, where particular bacterial genera, including *Alicycliphilus*, *Macellibacteroides*, *Dokdonella*, *Ahniella*, *Thauera*, and *Zoogloea*, associated with ARGs, exhibit decreased abundance at elevated temperatures. However, Syafiuddin and Boopathy [352] reported that these factors can vary considerably between genes and across studies. In addition, Wang et al. [353] noted an improvement of up to 52.50–75.07% in the removal efficiency of total ARGs in sewage sludge pretreated differently through alkaline, thermal hydrolysis, and ultrasonic pretreatments. Han et al. [354] affirmed that pretreatment methods are performed prior to anaerobic digestion to enhance the performance of the process, and these methods are different and impact the process at different magnitudes. The authors further confirmed in their findings that thermal hydrolysis demonstrated the best performance in removing ARGs as opposed to other pretreatments, including free nitrous acid, free ammonia, ozone oxidation, alkaline, microwave-based, and ultrasonic pretreatments.

Seemingly, Olivier et al. [355] affirmed that because of the difficulties encountered in operating a full scale digester, the performance of anaerobic digesters operating at full-scale in the field, designed for removing ARGs, is not well understood and could vary from that of laboratory-scale studies. Burch et al. [345] pointed out that laboratory-scale and pilot-scale operations of anaerobic digester systems often reflect better control of the anaerobic digestion process; their results may not be a true representation of the real world. The authors further emphasised that laboratory-scale operating anaerobic digesters are usually batch operated, ensuring all the feedstock is exposed to the set residence time and has a relatively long residence time, which can promote the removal of ARGs to a greater extent. In addition, the laboratory- and pilot-scale digesters are relatively small, allowing easy control of temperature and mixing, unlike full-scale digesters. Therefore, they are relevant to serve as initial screens for experimental treatments (e.g., multiple temperatures and substrates) that cannot be easily manipulated at full-scale facilities destined for commercial purposes [356]. On the other hand, full-scale digesters are often operated as continuous or semi-continuous reactions with relatively short hydraulic residence times, and their designs do not permit all feedstocks to remain in the digesters over the specified residence time due to economic pressures and logistical factors at commercial livestock facilities [345]. Notwithstanding, Olivier et al. [355] opined that the findings obtained from full-scale operations give a representation of realistic conditions, which are not constantly steady or well controlled in terms of operational factors, entailing temperature, OLR, and mixing. The authors further expressed that the full-scale studies provide a more likely reflection of realistic mixtures and concentrations of selective agents such as antibiotics and heavy metals that might impact the antibiotic resistome in dairy manure and other substrates. Burch et al. [345] equally noted both season and facility-level effects on the logarithmic removal of ARGs via anaerobic digestion. The authors further revealed that there exist variations between facilities, and many may require optimisation to realise their full potential, and other management practices on farms could have equal or greater impacts on ARGs than anaerobic digestion.

The methods used to target or detect the ARGs studied, including culture-dependent (qualitative method) or culture-independent methods (e.g., traditional quantitative polymerase chain reaction, high-throughput polymerase chain reaction, or metagenomics), should also be considered [340,357]. Both qualitative and quantitative methods employed in detecting the ARGs have discrete strengths and limitations or weaknesses. Yamin and colleagues [358] highlighted the significance of culture-dependent methods in providing detailed insight into the phylogeny of antibiotic-resistant bacteria (ARB) and resistance phenotypes, in addition to recognising the networks of ARGs and ARB between clinical and non-clinical samples. However, the effectiveness of these methods in characterising the environmental resistome is faced with the main limitation of the large proportion of environmental microbes that are non-culturable under certain conditions [359]. On the other hand, culture-independent or non-cultural approaches, including quantitative polymerase chain reaction (qPCR) and metagenomics, are molecular biotechnologies and high-throughput sequencing techniques [360]. The developed techniques rely on nucleic acids extracted directly from the samples to enable the documentation of an environmental resistome [361]. These methods are directed or targeted at the nucleic acids and provide both qualitative data, indicating the presence and absence of ARGs, alongside quantitative data, allowing comparisons across samples and studies [340]. Notwithstanding, qPCR demonstrates numerous advantages over metagenomics by possessing a higher detection sensitivity with broad ranges of detection, shorter turnaround time from sampling to results, more cost-effectiveness, and having well-established protocols with multiple commercial software packages, etc. [361]. However, real-time quantitative polymerase chain reaction (qPCR) is fraught with technical mishaps such as the presence of inhibitors (e.g., metals and humic acid) of the reaction and amplification bias derived from primer design, template GC contents, different instruments, sample matrix effects, and the expertise of the operator [361]. In particular, metagenomics is very relevant for comprehensive surveillance of the environmental resistome, allowing the possibility for a wide array of ARGs to be detected, while qPCR is said to be highly effective for the routine monitoring of certain ARGs, especially when the profile of the ARGs is well-defined. Wang and colleagues [340] pointed out the advantage of an integrated approach, in which both methods can be combined to maximise the strengths of each method.

Thirdly, the subtypes or resistance mechanisms categorised as efflux pump, cellular protection gene, and enzymatic inactivation genes, or antibiotic deactivation and other unknown mechanisms, can determine the fates and behaviours of the ARGs. In terms of trends in specific ARGs with reduction, *aad*A and *str*B confer resistance on bacterial strains to aminoglycosides and streptogramins, respectively, and are described most times as plasmid-borne, being more susceptible to heat stress and instability as plasmid DNA; therefore, resulting in a loss or degradation of the plasmid carrying the gene [362]. Similarly, the *cml*A gene occurs in bacterial strains and encodes an efflux pump, which is highly sensitive to heat, causing its disruption at elevated temperatures. However, this ARG causes resistance against the antibiotic chloramphenicol [49]. In addition, *bla_OXA_* and *bla_TEM_* genes encode beta-lactamases (enzymes) that hydrolyse beta-lactam antibiotics but possibly lose their enzymatic activity due to protein denaturation following their exposure to increased temperatures [363]. Other ARGs, including *sul1*, *sul*II, and *tet*A and *tet*M genes, confer resistance to sulfonamides and tetracyclines, respectively. Reductions are observed with *sul*1 at elevated temperatures as dihydropteroate synthase enzymes develop thermal instability, indicating that heat disrupts their functions and reduces enzyme resistance [364]. Contrarily, *tet*M offers resistance against tetracyclines, and it appears more structurally stable at higher temperatures; it is more resilient or adaptable to heat-induced stress. Lastly, the *erm* B gene presents resistance to macrolide, dfrA to trimethoprim, while *intl*I is the integrase gene, which represents mobile genetic elements and is defined as a marker for class I integron, indicating the possibility of resistance gene transfer without necessarily being predominant [49]. The mobile gene elements (MGEs) are DNA segments that can move within or between genomes and are involved in the HGT process to cause ARGs transfer [365].

Among these classes of ARGs, the beta-lactam resistance genes can be easily reduced due to their instability [366]. Under alkaline and acidic conditions, such as in the presence of organic acids and ammonia produced during the anaerobic co-digestion processes, the hydrolysis and degradation of beta-lactam antibiotics are promoted, eliminating the selective pressure of beta-lactam on microorganisms that leads to a decrease in beta-lactam resistance genes [196]. Thus, the authors emphasised that the persistence of antibiotic-resistance genes presents threats to horizontal gene transfer when sludge is directly applied to soils mediated by transposons, integrons, and plasmids via transformation, transduction, and conjugation processes [367]. In the process of transformation, bacteria take up, integrate, and functionally express any freely available DNA in the environment. Transduction can be described as generalised or specialised, and it makes use of the role of bacteriophage. Through the bacteriophage, DNA is transferred from the donor to the recipient cell [368]. Conjugation explains cell-to-cell contact between the donor and the recipient through a pilus [369].

Of great consideration is any alteration in community dynamics. Microorganisms are the key active players in an efficient anaerobic digestion process, among other abiotic environmental parameters. However, some of these microorganisms harbour ARGs. Therefore, changes in the microbial dynamics can impact the fate and removal of ARGs in the process [337]. This may suggest that the change in ARGs detected could be directly associated with certain groups of microorganisms harbouring them and their capacity to grow under the tested conditions. In addition, the alteration in the detected ARGs could be attributed to the ability of genetic elements harbouring the individual resistance genes to be distributed among a broader scope of bacterial genera or be more constrained within some groups [370]. Accordingly, Li and Yuan [371] noted the enhanced proliferation of ARGs and mobile genetic elements based on the dosage of microplastics (e.g., polyethylene, polyethylene terephthalate, and polylactic acid), and the abundance of the ARGs increased, occurring at 29.90%, 18.64%, and 14.15%, respectively, during the anaerobic digestion of sewage sludge.

### 3.3. Biofertiliser Production

Digestate signifies the digested materials discharged from the anaerobic reactor following biogas recovery. It is described as a semi-solid, fibrous solid and liquid mass containing both organic and inorganic matter [179]. The mineralisation of organic matter in the feedstock during anaerobic digestion leads to the formation of inorganic compounds or nutrients, which are present in plant-usable forms at markedly higher levels as opposed to their level in the original/undigested feedstock [372]. It appears to be a rich source of nitrogen (N), phosphorus (P), potassium (K), sulphur, several micronutrients, and organic matter [11]. Apparently, the composition of the digestate is affected by the feedstock type and the digester’s parameters, including temperature and pH [373]. Following anaerobic digestion of animal manure, bacterial pathogens become inactivated, making treated manure better in quality than raw or untreated manure, thus offering better beneficial effects that contribute to the health of the soil (particularly, the metabolic activities of soil microbial biomass), in addition to minimising environmental pollution and public health effects.

The application of digestate as an organic fertiliser represents an alternative for sustainable agriculture that can provide adequate nutritious food for all, alongside decreasing the risks to the environment [9]. The fertiliser property of a digestate is defined as its usefulness in promoting plant growth [374]. Tampio et al. [375] stated that the fertilising value of the digestate is affected by the content of heavy metals, pathogens, and the substrates utilised as feedstocks. However, the limited use of digestate for fertilisation is observed based on its quality, sanitary safety, possible land contamination, nutrient content, volatile emissions, or even scarcity of fertile land [376]. Soil health is the capacity of the soil to provide a milieu or act as a vital living system for optimum growth and development of plants, thus improving soil ecosystem function while equally ensuring the health of humans and animals as well as maintaining or improving water and air quality [71,377].

As the digestate emerges from the biodigester after anaerobic digestion, the optimal fertilisation properties can be lost if it is not fully stabilised or contains biodegradable substances [11]. To solve these problems, numerous processing procedures can be employed to convert the digestate into value-added by-products. Increasing the profitability of the digestate involves the extraction of reusable nutrients to encourage biogas plant cost-effectiveness, sustainable management, and the circular economy [97]. The post-treatment techniques are implemented to ensure that the quality of the biofertiliser becomes compliant with environmental standards based on the country’s policies. Usually, digestates obtained following anaerobic digestion of manure or co-digestion with co-substrates are spread on agricultural land. However, their hygienic status based on pathogens must be verified to conform to the regulatory policies of the specific region or country [203]. The level of indicator pathogens (*Salmonella*, *E. coli*, and faecal coliforms) highlights the hygienic status of the bio-digestate, and the standards/levels vary from one country to another [209]. Surprisingly, Cermak et al. [378] emphasised that even though the level of the indicator is below the detection limit, it still cannot be regarded as without any potential pathogenic threat due to the presence of other pathogens. The author mentioned further that the presence of pathogens in both manure and digestates suggests the persistence of selected bacteria throughout anaerobic digestion. This is backed by the findings of Avery et al. [328], who stated that pathogen concentrations are reduced by only 1–2 log units via mesophilic anaerobic digestion. Therefore, the possibility of having pathogens in the digestate following anaerobic digestion performed at mesophilic temperatures.

Nevertheless, Jiang et al. [209] reported that several countries have set biosafety regulations for pathogenic bacteria in anaerobic digestion, but the standards tend to vary from country to country. Apparently, in certain nations, the concentration of pathogens in anaerobic residues is regulated, and the residues from anaerobic digestion can be permitted for application on land, unless they meet the requirements of environmental quality. In Kampala (Uganda), McCord and colleagues [68] monitored the microbiological quality of feedstock and digestates recovered from seven planted biodigesters and found that digestate samples did not meet standards for wastewater discharge or international safety standards for field application. The authors further explained that even though they had good levels of total Kjeldahl nitrogen and phosphorus, presenting as a suitable source of fertiliser, the digestates may provoke issues of water quality if not properly managed. Often, digestate is stored prior to land application to further reduce the pathogen level [205]. However, this practice has been observed with the potential for pathogens to regrow [311]. Stocker and colleagues [379] emphasised that the actual number of pathogens shed and the release rate of the pathogen from manure determine the rate and efficiency of transport of these pathogens found in manure into water bodies. Alegbeleye and Sant’Ana [380] substantiated that the release of pathogens from manure is a crucial factor, depending on its composition, since it regulates the availability of the pathogen for transport into environmental matrices. The viability and survival of several enteropathogens are quite challenging outside the host [381].

In minimising the potential for human infections by the residual pathogens in the digestate, knowledge of the fate of the pathogens upon land application becomes vital, and it is obtained from the understanding of the survival characteristics of the different pathogens [205]. The survival rates in the environment can be from days to months and even years, depending on the pathogen, environmental conditions (temperature, pH, and permeability of the soil), time of the year, active microbial movement, microbial surface properties, soil water content, medium (i.e., soil type), digestate form (liquid or solid fractions), handling and post-treatment method, and the presence or absence of plants [382,383]. On the other hand, the digestate serves as a channel in transporting pathogens from agricultural land via the food chain to humans. Thus, proper sanitation becomes obvious. Further treatment of the digestate is imperative to obtain a more efficient reduction in pathogens. The sanitary level or microbiological quality of the digestate can be affected by factors such as the quality of substrates or feedstocks used to charge the reactor, the temperature of the process, the reactor performance, slurry retention time, pH, and ammonium concentration [11].

Overall, anaerobic digestion systems have the potential to improve waste handling and meet standards, coupled with fertiliser application with proper post-digestion treatment. The efficient post-treatment of digestate is important to convert it from waste to resource and increase the options for its careful reuse [384]. Ekstrand et al. [58] argued that full hydrolysis of hard-to-degrade organic matter is not met in anaerobic digestion. Therefore, residual proteins and lignocelluloses have been widely detected in digestates from full-scale biogas processes. Similar observations have been noted with digestates recovered from biogas processes experiencing unstable process conditions (e.g., ammonia inhibition, trace element deficiency, and/or short residence time). Besides biomass recalcitrance based on its composition of lignin, hemicellulose, and cellulose, the semi-continuous operation of digesters, non-optimised process conditions, and economic reasons could be other reasons for the incomplete biomass degradation [12]. Romio et al. [12] affirmed that only 40–70% of the biomass substrate is transformed into biogas; thus, a substantial level of biodegradable organic matter and biogas potential remains in the digestate. To optimise substrate utilisation and lessen methane emissions during digestate storage and handling, the residual biogas potential needs to be recovered via further exploitation of the digestate. Accordingly, Cerda and colleagues [385] reported on a novel approach, solid-state fermentation (SSF), an attractive technology that allows digestate to be employed as a feedstock for biotransformation into value-added products, including hydrolytic enzymes, biosurfactants, aromas, biopesticides, or bioplastics. This technology is currently receiving great attention. Similarly, Isemin and colleagues [386] applied torrefaction or pyrolysis to recycle the digestate recovered during anaerobic digestion.

In addition, post-digestion processing can further lessen the risk accompanying the land application of digestate to crops grown for human consumption [33,68]. Ekstrand et al. [58] opined that post-digestion treatment (secondary digester) is an easy way to lessen methane emissions that usually occur during the storage of digestate and application as a fertiliser. The authors recommended the coupling of post-digestion with other chemical, biological, and thermal treatment methods, as these will allow access to the residual proteins and lignocelluloses in the digestate. Accordingly, several methods, including chemical hydrolysis, thermal or heat treatment, composting, UV irradiation, and ozonation, can be embraced for the post-treatment of the digestate to further reduce pathogens beyond what anaerobic digestion achieves and ensure safe use as a fertiliser or in other applications. These different processes involve significant capital costs in terms of suitable machinery, high energy, and reagent consumption, and they result in varying physical characteristics and fertiliser value of the final product or digestate [387]. Cucina and colleagues [73] pointed out that post-treatment of digestate should achieve the removal of pathogens, the reduction in biodegradable organic matter, and nutrient modulation. Increasing biomass degradation via post-treatment of the digestate results in reduced volume of the final digestate, thus decreasing the costs of transportation to farmlands and incineration sites as well as the related greenhouse gas emissions. It is worth mentioning that a large variation in outcomes of these different methods is observed, which mirrors the differences in treatment conditions and compositions of the digestate [12].

Overall, the processes involved in the treatment of digestate can be grouped into two different approaches: partial treatment and complete purification, with the major purposes of volume reduction to improve manageability and decrease the cost of transportation, and, secondly, for the recovery of nutrients in concentrated forms [387].

Storage: The digestate occurs as the remaining liquid/solid residue following anaerobic digestion. Its quality or characteristics/parameters vary depending on both the type of substrate utilised in the anaerobic digestion process and the process conditions [11]. Prior to the spread of the digestate on agricultural land, it was usually stored. Storage of the anaerobic digestates is one significant aspect of the legal requirements before their application [388]. This procedure is suitable for improving the microbiological quality of organic fertilisers by reducing microbial pathogens. The reduction or inactivation of pathogens in the anaerobic digestates during storage occurs over a defined period or duration [388]. Apparently, the efficacy of inactivation of pathogens during storage is partly influenced by the management and storage conditions, especially the temperature at which the process takes place. Schilling and colleagues [388] demonstrated that pathogen inactivation was strongly affected by temperature and, to some extent, by the type of anaerobic digestate (i.e., the kind and nature of substrates used as input material will influence the pH and the dry matter of the recovered digestate). The different bacterial species respond differently to the effect of temperature during storage. The authors registered a 5-log reduction in *S. typhimurium*, *E. coli*, and *L*. *monocytogenes* after 12 weeks, 20 weeks, and 24 weeks, respectively, in anaerobic digestates stored at higher temperatures (spring and summer profiles).

According to Kumar and Kanwar [389], the shorter or longer survival of the different bacterial species under identified conditions could appreciably depend on the inherent properties of the bacteria alongside biotic and abiotic factors. Therefore, the required duration of storage of digestate can be influenced by the environmental restrictions for application, digestate stabilisation, and its demand, soil and crop type, along with the geographic location [387]. The storage of digestate equally requires huge costs and land occupation. However, this practice is faced with the challenge of regrowth of pathogens, as this may occur [11]. As a matter of fact, the storage of every anaerobic digestate should be handled and assessed individually, considering the makeup of the anaerobic digestates and storage conditions, as there are no general biosafety recommendations available that are applicable to all biogas plants and anaerobic digesters.

Solid–liquid separation: This is a simple and low-cost-effective approach, which is usually performed prior to any further post-treatment of digestate, as it separates the digestate into two fractions, the solid and liquid fractions. Such separation tolerates enhanced management of the fractions, e.g., fewer processing and equipment requirements for storage [94]. Firstly, the solid concentration of the digestate is increased through sludge thickening, thereby reducing the water content. This process can take place naturally, under gravity (gravity settling), or mechanically (screw press). Moreover, Hyder et al. [384] expressed that polymers could be added to digestate to increase solid–liquid separation and reduce the volume. Monfet et al. [390] highlighted the different standard mechanical dewatering procedures, viz., screw press, centrifugation, filtration, air floatation, vacuum filtration, and chamber filter presses, together with the belt filter presses. The solid fraction, which is described as pressed cake, concentrates dry matter (between 20 and 30%) and nutrients, mainly organic N and P [391], and is generally used as an organic amendment.

On the other hand, the liquid fraction constitutes a dry matter content of approximately 2 to 6% and concentrates mineral N above all in the form of ammonia and thus can be employed as a substitute for mineral fertilisers [100]. Drosg et al. [392] remarked that solid–liquid separation is the very first step in the processing of digestates. It is a common practice, particularly following anaerobic digestion, which is significantly implicated in pathogen transmission because the separated solids and liquids are handled and managed independently [49]. The authors further observed a large difference in the distribution of microorganisms between the liquid and solid-separated fractions, as the microorganisms tend to lessen their attachment to solids when there is a high concentration of bioavailable organic matter in the substrate. Husfeldt et al. [393] noted that the separated solids can be reused as bedding for cattle animals, but the residual pathogens in this portion occur as a health risk to the animals and can contaminate raw milk owing to direct contact with the animals. In addition, Schilling et al. [388] noted the possible risk of transmission of infection to humans due to the potential contamination of groundwater or food, as well as via aerosols. Alternatively, the separated solid fraction can be composted, dried, and incinerated [378]. McCord et al. [68] expressed the benefits of safer and more cost-effective transport of lightweight solids to cropland for land application rather than hauling and applying high-moisture digestate.

In addition, Cameilleri-Rumbau et al. [394] pointed out that the liquid can be further concentrated using membranes; however, membrane fouling is the key issue challenging the use of membranes during processing, limiting the continuous operation of the membrane. On the other hand, the liquid portion can be used for irrigation, and, similarly, the residual pathogens in this fraction can contaminate surface water and groundwater via infiltration or surface runoff [149]. In addition, the nitrogen and nutrient content of the digestate can cause nitrogen leaching and pollution of groundwater and surrounding rivers, hence necessitating storage capacity until further treatment [94]. This procedure involving the liquid digestate fraction describes recirculation, which offers the potential for heightening the reduction in pathogens and widely lessens the demand for these systems by freshwater bodies. Kolar et al. [395] noted that the liquid fraction is regularly applied on arable land or permanent grassland, or, based on technology, it can be reverted into the biogas processing plant to carry out the fermentation process. Therefore, causing a serious upgrade in communities where freshwater resources are limited [68].

In addition, Sfetsas and colleagues [94] registered that biological, physicochemical, and mechanical procedures (e.g., reverse osmosis, algae treatment, and struvite precipitation) can be employed for nutrient recovery and removal from the liquid fraction. The authors further explained that these methods are complex and differ in terms of economic value, legislative restrictions, and performance, as well as feasibility. Notwithstanding, parameters such as topography, liquor characteristics, climatic conditions, and available resources should be checked, as they tend to influence the selection of the procedures.

Thermal post-treatment: It is necessary as a method of hygienisation of some biowastes to eliminate pathogens [183]. The thermal hydrolysis process (THP) occurring at 134 to 175 °C, for 20–30 min, and moderate thermal treatment (70 °C for 1 h and 55 °C for 24 h) has been examined for the post-treatment of digestate [396,397]. Nordell and colleagues [396] highlighted that moderate thermal treatment is favourable for post-treatment and represents the treatment conditions needed for digestate hygienisation. Thus, suggesting that thermal treatment is considered an effective option for the inactivation of pathogens. Svensson and colleagues [397] added that the application of treatment on digestate is rather more energy efficient than on substrate because the volume and quantity of materials that need to be subjected to heating have been drastically reduced following primary digestion.

Moreover, Abdelfatah-Aldayyat et al. [398] mentioned that gasification, pyrolysis, and hydrothermal carbonisation are alternative thermal treatments transforming digestate into valuable syngas, obtaining, in many cases, a carbonised stream referred to as biochar. The authors added that the feasibility of the process rests on these key parameters: (i) energy demand for the drying stage and (ii) available treatments intended for the removal of contaminants from syngas, achieving high-quality products, and treating the process-derived water. In addition, Bjerg et al. [79] in their findings noted that the thermal treatment (70 °C for 1 h) of digestate improved biomethane production during post-digestion by 21–22% (food waste-digestate) and 9% (agricultural waste-digestate). Overall, Kovačić et al. [14] highlighted that the exposure time (i.e., the time needed for the inactivation of the pathogens) may be influenced by factors such as uneven heating, changing temperatures, or shielding properties of solids. However, this procedure is faced with major technical and financial efforts by the operator [388].

Chemical post-treatment: Chemical treatment is regarded as a conditioning method. This relies on the use of chemical agents to alter or change the properties of the digestate [399]. Chemicals such as hydrogen peroxide, ferric chloride (Fe_2_Cl_3_), calcium hydroxide [Ca(OH)_2_], etc. Asensi et al. [400] mentioned that Fe_2_Cl_3_ acts as a coagulant, creating iron hydroxide, which is of low solubility and presents as bridges between particles, forming compressed and bigger flocs that settle rapidly. Free hydrogen is released from the digestate following the addition of the trivalent cation 3+, thus resulting in a decreased pH of the solution. Accordingly, Ca (OH)_2_ is added to the digestate to adjust its pH. Under alkaline pH conditions, organic matter becomes more soluble, decreasing the surface tension of water, causing it to flow more quickly, rendering it easily removed [401].

Composting: The biodegradation of organic matter in digestate via composting is viewed as a vital pathway for carbon flow and nutrient recycling in both developing and developed countries [402]. It is a biological process performed by microorganisms in the presence of oxygen (aerobic conditions). The process describes the degradation and stabilisation of organic wastes through four phases: mesophilic phase (10–42 °C), thermophilic phase (45–70 °C), cooling or middle mesophilic phase II (65–50 °C), and maturation or curing or finishing phase (50–23 °C) to yield compost [403]. Therefore, the process of composting transforms the digestate into a mature, stable, safe, humus- and nutrient-rich compost that presents an appropriate advantage over the digestate in maintaining and improving environmental quality and conserving resources [11]. “Compost” is derived from the Latin word “compositum” (meaning consisting of more than one substance). Accordingly, Kovačić et al. [11] mentioned that compost is regarded as a vital source of organic matter and nutrients for agriculture and, when used as a component in the preparation of pot substrates, performs a crucial role in upholding soil biodiversity and horticultural production. This self-heating, aerobic, solid-phase process can be described as nature’s way of recycling that involves microorganisms (including bacteria, fungi, and actinomycetes and their enzymes), which can be categorised into different classes of microbes as mesophiles and thermophiles [402]. Moreno and colleagues [404] noted that the dynamics of the microbiota during the composting of wastes are very much tightly coupled to the environmental conditions occurring inside the compost pile, as described, viz., the availability of nutrients, alterations in temperature, the starting materials, the oxygen concentration, pH, along with the size of the particle. In a study performed by López-Gonzáloez et al. [405], both culture-dependent and culture-independent approaches were applied to delineate the wide variety of microbial populations associated with composting. Jurado et al. [406] affirmed that the culture-independent techniques provide an advantage over the counterpart techniques, allowing for the identification of unknown sequences that can be ascribed to new taxa, therefore unravelling the great variety of mesophilic and thermophilic organisms that occur within the multifaceted environment of the compost.

The mesophile microbes engaged in the process are responsible for initiating the process of composting at temperatures between 30 and 50 °C. Following the formation of compost piles, the activity of microorganisms increases in tandem with the temperature plus density within the piles, and the process is taken over by thermophiles beyond 50 °C. There is an observed rapid rise in temperature in the compost from 50 to 70 °C within a day to three (3) days following pile formation. The temperature remains or is maintained at this level for several days based on the characteristics of the substrate, the size of the pile, and the environmental conditions [402]. This phase is described as the “active phase”, where hygienisation occurs. It is also at this phase that the most rapid decomposition occurs and continues until every material containing nutrients and energy within the pile is transformed. Subsequently, the microbial activity reduces, resulting in a gradual drop in the temperature within the compost pile to nearly 27 °C. The compost pile becomes recolonised by mesophilic microorganisms, and the process progresses to the curing phase. During the curing phase, the oxygen consumption by the microbes declines while organic matter continues to degrade and become converted to humic substances that are biologically stable [407].

As a biological process, composting can further reduce the pathogen concentration of the digestate [68] and, ultimately, convert digestate into mature, stable, safe, humus- and nutrient-rich compost [11]. Pathogen reduction occurs during the maturation phase. However, the process is associated with greater technical and logistical efforts [388]. In addition, Alegbeleye and colleagues [381] noted as a drawback that this technique, like anaerobic digestion, needs monitoring of temperature and holding times to achieve the inactivation of pathogens, and attention is needed to prevent the risks of the regrowth of pathogens. This is because agricultural soils must conserve acceptable levels of quantity and quality to produce food, fibre, and energy without becoming vulnerable to a negative impact on their nutrients and health balance or their potential to function [397]. Managing soil health is very significant for biodiversity conservation and safeguarding sustainable agricultural production; overall, soil health has primary importance for ecosystem sustainability [408].

Summarily, the microbiological quality and safety of anaerobic digestates are paramount in a bid to decrease the microbiological risk potential associated with this category of organic fertilisers prior to their applications in different scenarios [388]. In this light, three options are feasible, including inactivation of pathogens during the fermentation in the biogas plant, pretreatment of waste before charging the anaerobic biodigester, and lastly, the disinfection of the anaerobically digested digestate discharged from the biogas plant. Overall, the survival behaviour of the pathogens in anaerobic digestates is determined by a host of environmental factors, including temperature, indigenous microbiota, moisture, pH, and particle size. Apparently, Άlvarez-Fraga et al. [53] noted that it is vital to develop and implement detailed management and risk assessment protocols throughout the whole anaerobic digestion life cycle. Such practices, regulated at a national and international level, play a critical role in safeguarding the environment together with public health.

## 4. Mitigation of Biomass Recalcitrance in Anaerobic Digestion

Biogas can be employed in cooking or harnessed to produce electricity, while the digestate produced can be post-treated and employed as a biofertiliser to improve the growth of crops and plants needed for human consumption [409]. Anaerobic mono-digestion of lignocellulosic wastes is faced with challenges of process instability caused by the accumulation of volatile fatty acids and decreased biogas yield, owing to biomass recalcitrance offered by this group of wastes. Nevertheless, the situation can be addressed via pretreatment of the substrates and co-digestion with other substrates to ensure an adequate supply of nutrients, microorganisms, and better operating conditions, leading to improved biogas yields [410]. The mono-digestion of animal manure is hindered by an imbalanced C:N ratio, which eventually leads to decreased or low potential methane yields [411]. Regardless of the potential presented by lignocellulosic biomass, e.g., plant waste as a suitable substrate for anaerobic digestion, the materials display a resistance to degradation termed biomass recalcitrance. This is caused by their natural composition, ascribed to the crystallinity of cellulose, and the encapsulation of the lignin-hemicellulose added to the hydrophobicity of lignin [412]. Zhao et al. [413] demonstrated that recalcitrance leads to low yields of products and accumulation of undigested biomass and thereby decreases the overall economic feasibility of biochemical production processes. Furthermore, other lignocellulosic substrates (e.g., food waste) have high carbon content and low nitrogen levels, imbalanced trace elements, and a lack of diversity in the microbial community, and the effect originating from other operational factors could cause inhibition problems during their mono-digestion [414].

Anaerobic digestion of lignocellulosic wastes with co-substrates within a biodigester is observed as a turning point in the energy crisis, waste management, and hygiene. This practice culminates in a balanced C:N ratio between animal manure and plant-based substrates, taking advantage of the inherent buffering capacity, microbial populations, nutrients, and moisture content of animal manure. Hence, the relatively low methane yield is improved [415]. The two categories of lignocellulosic waste have different/complementary characteristics; therefore, co-digestion helps in the improvement and optimisation of the anaerobic digestion of both wastes separately. Aboudi et al. [147] stated that co-digestion of animal manure (e.g., pig and cow) and sugar beet by-products mitigated the inhibitory effect of volatile fatty acids at high organic loading rates, which led to increased production of methane by 70 and 31%, respectively, relative to individual digestion of sugar beet by-products. With reference to the aforementioned findings, the type of manure has a vital role to play in the co-digestion process. Manure tends to differ, which in turn affects the anaerobic digestion process differently, as some may be more effective than others during the co-digestion process with agro-industrial substrates. This discrepancy could be attributed to the composition of each manure, which is determined by its origin (pig or cattle, etc., digestive tracts), animal feed administered during animal husbandry, and the synergy that exists between the animal manure and the agro-industrial co-substrate [416]. Nolan and colleagues [326] opined about the multi-beneficial effects of co-digestion ascribed to energy recovery, elevated farm incomes, and reduction in noxious gases. Moreover, Xie et al. [417] expressed that even with the numerous benefits associated with co-digestion, the blend of substrates with different characteristics could create synergistic interactions or antagonistic interactions, which in this case exacerbate the anaerobic process.

The biomass recalcitrance can be addressed by subjecting the materials to pretreatment, performed via biological, physicochemical, thermal, and biological approaches [24]. Pretreatment of lignocellulosic wastes and anaerobic co-digestion of lignocellulosic wastes are the different approaches through which the anaerobic digestion of lignocellulosic biomass to recover energy will be positively augmented, resulting in a stable process and improved yields in biogas [62].

### 4.1. Pretreatment Methods

The choice of pretreatment is one of the key players governing the biogas yield, particularly when dealing with lignocellulosic feedstocks [418]. Pretreatment of the lignocellulosic material is vital in preparation for anaerobic digestion as it may result in physical or chemical changes, causing alterations in the size and porosity of the particle, facilitating the penetration of solvents and catalysts. Pretreatment occurs prior to biological deconstruction to increase the accessibility of lignocellulose to biological attack, thereby improving the efficiency of subsequent bioconversion processes [419]. Apparently, the process might affect the crystallinity of cellulose, the linkage between the three molecular compounds (cellulose, hemicellulose, and lignin), and the degree of polymerisation [1]. Overall, the pretreatment methods can be categorised into chemical, physical, biological, and enzymatic pretreatments. He and colleagues [399] remarked that the different methods are associated with both advantages and disadvantages, and yet were said to function as pretreatments in (i) improving the surface characteristics of substrates and enhancing the adaptability of microorganisms, (ii) reducing/eliminating potentially toxic and harmful substances, and (iii) improving the biodegradability of complex compounds. The nature of solvents and lignocellulosic materials, plus the cost of the pretreatment method, are of great consideration in the choice of pretreatment to be employed. Owing to the variation in lignocellulose across this group of biomass, the different types of substrates respond differently to the different pretreatment techniques. Therefore, no single pretreatment method is ideal for all substrate types. Consequently, each method must be assessed in detail to delineate its advantages and disadvantages [24]. Among these challenges are cost, operational robustness, chemical recycling, and/or production of compounds that inhibit processing.

The different pretreatment methods require different levels of energy consumption and exert differing effects on the degradation of the lignocellulose. Therefore, while choosing a pretreatment method, consideration should be taken regarding energy balances and economic feasibility [62]. In this light, Olatunji and colleagues [24] commended the use of two or more of these pretreatment methods as opposed to a single treatment, because it is economical, creating substantial improvement in the effectiveness of the process. Pretreatment is achieved only when the feedstock composition appropriately corresponds to the pretreatment technique, aiming for optimal biogas yields. Contrary to pretreatment, Bharadwaj et al. [419] noted that cotreatment, which reduces recalcitrance, takes place during fermentation. The authors reported that cotreatment for 0.5–10 min increased sugar solubilisation by 5–13% as opposed to the unmilled control, with larger solubilisation correlating with improved milling duration. Cotreatment in anaerobic digestion systems, therefore, provides an alternative approach to conventional pretreatments to increase biogas production from lignocellulosic grassy material.

### 4.2. Co-Digestion

Co-digestion involves treating combined or multiple wastes to produce biogas and digestate while recycling nutrients from wastes back into food production, thus presenting as a vital activity in sustainable farming and the circular economy [147]. The mono-digestion of both lignocellulosic biomass and animal manure is faced with challenges because of biomass recalcitrance and inadequate nutrient supply, respectively. Manure is described to contain high levels of nitrogenous compounds, which tend to elevate the level of free ammonia and ammonium ions produced, hence provoking inhibition by ammonia [420]. Achi et al. [421] explained that animal manure, a nutrient-rich substrate, could reduce the carbon-to-nitrogen (C: N) ratio and provide buffering capacity for stabilising the pH in order to increase methane (CH_4_) production. In addition, animal manure is already predigested in the animal’s intestines; thus, its mono-digestion results in reduced biogas yield [62]. Owing to the occurrence of rumen microbes in animal manure that are endowed with great potential to demonstrate anaerobic digestion at a faster rate than plant microorganisms, animal manure is usually employed as a base substrate for co-digestion with other substrates, more especially because of its ready availability in agricultural farms [145]. Li and colleagues [422] suggested that combining livestock manure with vegetable or crop residues could improve the efficiency of anaerobic digestion, energy efficiency, and profitability. Co-digestion addresses the low nutrient levels in particular wastes, which are insufficient for standalone anaerobic digestion, presenting it as a viable option for improving methane yield [423]. Achi and colleagues [421], in their findings, explained that rising concentrations of biochar and zeolite produced alternating effects on carbon dioxide (CO_2_) and methane (CH_4_) generation, with cassava wastewater greatly contributing to CH_4_ production.

Knowledge of the potential of profitability is of great value to farmers, policymakers, communities, and leaders to assess the costs and returns and make better decisions [424]. High biogas and methane yields are possible when pretreatment is performed prior to co-digestion. Co-digestion is the term used to describe the process involving the anaerobic digestion of multiple substrates. Thus, a great chance of treating two or more or a wide spectrum of substrates helps in reducing the quantity or volume of waste available for disposal, resulting in sanitised wastes rendered safe for human handling via the inactivation of pathogenic microbes in the wastes during the process [62] and harbouring plant growth-promoting factors necessary as biofertiliser to enhance plant growth and crop yield [145]. Apparently, it tackles waste problems, and the digestate recovered from co-digesting multiple substrates is usually very high in nutrient levels as opposed to that produced by anaerobic mono-digestion, which happens to involve just one or a single substrate (waste). Co-digestion equally enhances biogas production owing to the synergistic activities of microbes occurring in the different substrates as well as owing to the balance of nutrients and microbial components [92]. Furthermore, Borowski et al. [425] mentioned other advantages of co-digestion, including dilution of toxic substances, increased buffer capacity, enhanced loading of biodegradable organic matter, and greater digested product quality. According to Suhartini et al. [410], co-digestion is a sustainable process that improves detoxification from toxic substances.

### 4.3. Supplementation of Additives

Nutrients are essential for every microbial species in monitoring their growth, metabolic, and enzymatic functions. Consequently, they play a vital role in anaerobic digestion in the functioning of microorganisms [426]. Some substances or elements, such as trace elements or metals, are employed as additives in the anaerobic degradation process involving lignocellulosic wastes to promote the performance of the process via enriching microorganisms, increasing biogas yields, and shortening the digestion time [399]. In addition to the trace elements, Altamerano-Corona et al. [427] gave examples of additives to include biological (fungi, microbial consortium, enzymes), chemical reagents, macronutrients, minerals (copper, nickel, manganese, molybdenum, zinc, and iron), transition metal compounds, and carbon materials. Moreover, Hassanein et al. [428] mentioned biochars, hydrochars, zeolites, and modified hydrochars and biochars as other additives employed in anaerobic digestion, which contributed significantly to increased methane production by enhancing the synergistic microbial interactions between different microbial species, through direct interspecies electron transfer (DIET).

DIET is critical for enhanced and efficient bio-methanation. Almegbl et al. [429] narrated that DIET alters metabolic pathways expressed through microbial diversity, abundance, and associated enzymes, resulting in biogas production and methane content, and augmenting volatile fatty acid and ammonia-stressed digesters. Zamel et al. [430] expressed that electrically conductive pili (e-pili) found in microorganisms facilitate DIET. For example, Geobacter produces conductive pili known as nanowires (containing Pil A protein monomers) that allow the transfer of electrons from the electron-donating species to the electron-accepting species across cell surfaces. Nguyen et al. [431] elaborated that microorganisms involved in DIET exchange directly through conductive pili or external conductive materials, sidestepping the requirement of intermediate molecules. This direct channel, particularly in the acetogenesis and methanogenesis steps, augments syntrophic metabolism by enabling faster and more stable electron flow between syntrophic partners [429]. The authors further confirmed that the integration of DIET into the anaerobic digestion process also boosts system resilience under stress conditions, such as volatile fatty acids or ammonia accumulation. Almegbl et al. [429] registered that different materials, including carbon-based sources (biochar, graphite, carbon fibres, carbon nanotubes, etc.) and iron-based sources (magnetite, Fe_3_O_4_, haematite, Fe_2_O_3_, goethite, FeOOH) act as intermediates in DIET.

In addition, the additives act by preventing the accumulation of toxic concentrations of ammonia, encouraging the formation of biofilm/anaerobic granular sludge, providing nutrient supplements, removing H_2_S, sequestering CO_2_, and incorporating bio-augmentation [421]. The choice of the additives is determined by the elemental analysis of the biomass. Pilarska et al. [186] added that the selection of the additives should be targeted to meet the exact needs and conditions of the digester to maximise benefits and guarantee sustainability. These may include magnesium oxide (MgO), iron chloride (FeCl_3_), or cellulase. Through microbial community analysis, Kim and co-authors [432] demonstrated that different additives provoked the proliferation of different microbial species. Microorganisms essential in the process of anaerobic digestion consist of enzymes whose activities bring about the degradation of the biomass. These enzymes need cofactors such as trace elements, e.g., cobalt (Co), nickel (Ni), selenium (Se), iron (Fe), molybdenum (Mo), and tungsten (W), to catalyse methane production. Trace elements, also described as micronutrients, are crucial additives needed in small quantities but are essential to perform a vital role in the metabolic pathways of microorganisms to enhance the efficiency and stability of biogas production via anaerobic digestion [126,186]. Combining these trace elements can provoke synergistic effects. In this light, Mary et al. [426] in their study supplemented the co-digesting mixture of food waste and cow dung in a biodigester with optimum dosages of micronutrients (1 mg/L of Fe, 0.5 mg/L of Cu, 1 mg/L of Zn, 0.5 mg/L of Mn, 1 mg/L of Mg, and 0.5 mg/L of Ni) and produced a biogas of 850 mL/g VS 10 day retention time. The authors further acknowledged an increase in the biomethane yields of the substrate by about 60% relative to reactors that had no micronutrient supplementation.

The overall functions of additives, as presented by Pilarska et al. [186], include the following: (i) Improved syntrophic interactions; (ii) Adsorption of toxic substances that may inhibit microbial activity; (iii) Improving microbial activity, and increasing process stability; and (iv) Accelerating the decomposition of complex organic materials, thereby increasing the rate of hydrolysis.

## 5. Conclusions

The interplay between the environment, animals, and humans is obvious, and the presence of crises in one of these domains or components can directly or indirectly affect the other because of the activities of humans and animals in the environment in the quest to sustain a living. Every human desires a healthy and convenient life, resulting in communication and relatedness with the environment, where crops and plants are grown. The over-exploitation of fossil fuel usage and the increase in energy demand have caused a decrease in fuel abundance in the earth’s natural reserves [433]. As a result, major nations are targeting to cut off fossil fuel usage and GHG emissions by supporting the Kyoto Protocol [434]. In addition, the rising demand for food by the population, especially meat and its derivatives, has caused the misuse and abuse of antibiotics in animal farming, resulting in increasing levels of antibiotic-resistant bacteria and their resistance genes. Antibiotic resistance is a global menace that is life-threatening; thus, it needs great attention. The microbial agents (antibiotic-resistant bacteria and their resistance genes) can be found in the gastrointestinal tract of animals, but can ultimately end up in animal manure. Since time immemorial, animal manure has been used as a biofertiliser to grow crops for both human and animal consumption. Therefore, creating a channel for the transfer of antibiotic-resistant bacteria and their resistance genes into the food chain. In a bid to mitigate the transfer of these microbial components, the animal manure, alongside other biomass, can be employed for treatment via anaerobic digestion within a closed container to reduce their concentrations (inactivation) while producing biogas, a clean and renewable energy that can complement or replace fossil fuels. This is the point of focus, and the process depends on various factors, mostly related to the substrates employed, microbial pathways, and the changing environmental conditions [435]. Mu and colleagues [184] described anaerobic digestion as a triple-benefit biotechnology for organic waste treatment, renewable energy production, and carbon emission reduction. In other words, AD is presented with multiple purposes: treating lignocellulosic wastes and animal manure for biogas production and ensuring the sanitisation of the wastes and the environment. Simply, anaerobic digestion produces renewable energy, functions as sustainable waste management, and permits the recycling of nutrients in organic waste for use as a fossil-free fertiliser [435]. The digestate, being of better microbiological quality than the untreated animal manure, can still be further treated to improve its quality, which will have a tremendous impact on the soil, crops, and eventually, humans through the food chain. Lin and colleagues [146] expressed that it has been a challenge to achieve efficient methanogenesis and effective sterilisation of wastes in anaerobic digestion. At the same time, however, combining the inactivation efficiency of pathogens and the energy production of the anaerobic digestion process will assist in guiding the practical design of the system.

## Figures and Tables

**Table 1:** Differential composition of agricultural residues and animal manures based on physicochemical and microbiological parameters.

Agricultural Wastes	Composition of the Waste		References
	Physicochemical	Microbiological (Before or During or After AD in Terms of Quantity or Diversity)	
Barley straw	Crude protein (3.8%), dry matter (90.0%)	Not applicable	Shah et al. [26]
Rice straw	Crude protein (3–7%), dry matter (92–96%)		
Cow dung	TS (77.33%), VS (67.65%), MC (9.68%)	TAMB (25 × 10^8^), yeasts and fungi (11 × 10^9^) cfu/g	El Asri et al. [27]
Horse manure	TS (83.29%), VS (74.5%), MC (8.7%)	TAMB (92 × 10^8^), yeasts and fungi (57 × 10^5^) cfu/g	
Chicken manure	TS (82.9%), VS (72.82%), MC (10.08%)	TAMB (11 × 10^4^), yeasts and fungi (12 × 10^8^) cfu/g	
Wheat straw	Crude protein (3.36%)	Not applicable	Praspaliareskau et al. [28]
Sorghum stover	Crude protein (6.6%), MC (8%), dry matter (91.25%)		
Sugarcane bagasse	Crude protein (3%), dry matter (89.8%)	*Lactobacillus*	
Corn stover	Crude protein (4.05%), MC (5.33%), dry matter (93.38%)	Not applicable	
Sugarcane tops	Crude protein (7.85%), MC (50–55%), dry matter (41.86%)		
Rice straw	Crude protein (3.15%), MC (4.62%)		
Pig manure	Copper, Zinc, quaternary ammonium	*E. coli*, *Salmonella*, *Trichuris* and *Trichostrongylus*	Beily et al. [29]
Finishers	pH (6.3), TS (18 g/L), VS (12.9 g/L);	Faecal coliforms (9.96 × 10^3^ MPN/mL), *E. coli* (9.50 × 10^3^ MPN/mL).	
Weaners	pH (6.5), TS (13 g/L), VS (7.6 g/L)	Faecal coliforms (8.4 × 10^3^ MPN/mL), *E. coli* (8.4 × 10^3^ MPN/mL).	
Cow dung	Dry matter (15.5%), organic matter (95.82%), C/N ratio (27.78)	Not applicable	Louh et al. [30]
Pig dung	pH (7.9), TS (29.9%), VS (84.2%), C/N (12:01); total Nitrogen (305), potassium (4.2), magnesium (32), manganese (0.028), iron (3.8), Zinc (12), aluminium (0.36), copper (2.3) [mg/L]	Not applicable	Ejigboye et al. [31]
Cow manure	TS (14%), VS (11.76), pH (6.55)	*Romboutsia*, *Turicibacter*, *Clostridium*, *Ruminococcus*, *Peptostreptococcus*	Castro-Ramos et al. [21]
Cattle slurry	pH (6.4), C/N (21:1); MC (84.42) VS (82), Ash content (4.06) [%]; calcium (261.42), nickel (5.38), iron (9.46), potassium (14.35), manganese (1.35), zinc (12.36), aluminium (3.23) [g/kg/TS]	Total viable counts (1.6 × 10^6^ cfu/100 mL)	Odekanle et al. [32]
Pig manure	pH (6.90–7.79); MC (87), TS (6.90–12.34), VS (70.07–81.18) [%], total nitrogen content (6.50), total sulphur (6.50–9.71), total ammonia nitrogen (6.09–7.62), phosphorus (54.60–76.42), potassium (23.60–62.25) [g/kg]; zinc 568.6–206.9, mg/kg and others (copper, chromium, nickel, lead, cadmium, mercury).	*Pseudomonas*, *Enterococcus*, *Lactobacillus*, *Streptococcus*, *Staphylococcus*, *Clostridium*, *Halomonas*, *Bacteriodes*, *Tissierella*, *Acholeplasma*	Li et al. [33]
Sheep manure	pH (7.86–8.44); MC (64.86–77.46) TS (22.54–35.14), VS (75.85–77.05) [%]; total nitrogen (16.46–20.22), total ammonium nitrogen (3.60–4.099), total phosphorus (4.67–8.14), sulphur (2.89–3.65) [g/kg] and others (potassium, chromium, nickel, lead, aluminium)		
Pig manure	pH (7.86), C/N (8.18); TS (31.93), VS (25.03), ash content (9.56) [%]	Not applicable	Tian et al. [34]
Rice straw	C/N (40.04); TS (94.79), VS (81.80), ash content (10.12) [%]		
Inoculum	pH (7.58), C/N (5.12); TS (13.57), VS (5.33)		
Cow dung	Carbon (20.0), hydrogen (3.56), nitrogen (7.17), sulphur (0.313), oxygen (69.0) [%]	*Clostridium*, *Lactobacillus*, *Enterococcus*, *Escherichia*, *Prevotella*, *Joetgalicoccus*, *Streptococcus*, *Romboutsia*, *Ruminococcus*	Mutungwazi et al. [35]
Chicken manure	Carbon (33.0), hydrogen (3.56), nitrogen (1.69), sulphur (0.205), oxygen (57.3) [%]		
Pig manure	Carbon (29.9), hydrogen (4.96), nitrogen (3.63), oxygen (0.234), oxygen (61.3) [%]		
Horse manure	Carbon (27.7), hydrogen (3.63), nitrogen (1.85), sulphur (0.219), oxygen (66.6) [%]		
Chicken manure	C/N ratio (9.77); TS (27.29), VS (23.33), total carbon (46.08), total nitrogen (4.728) [%]	*Lactobacillus*, *Bacteroides*, *Acidaminococcus*, *Clostridium*, *Msthanosarcina*, *Methanosaeta*, *Methanobacterium*, *Methanospirillum*, *Methanosphaera*, *Methanoculleus*	Feng et al. [36]
Corn stalk	C/N ratio (57.038); TS (91.44), VS (86.50), total nitrogen (0.877), total carbon (50.22) [%]		
Inoculum	pH (6.41); TS (18.12), VS (8.36) [%]		
Chicken manure	TS (16), VS (76) [%]	*Lactobacillus*, *Wiessella*, *Bacteroides*, *Proteiniphilium*, *Tepidimicrobium*, *Caldicoprobacter*, *Flexilinea floccule*, *Methanobacterium*, *Methanosaeta, Methanolinea*, *Methanoculleus*	Chen et al. [37]
Rice straw	Magnesium (3.68), calcium (24.07), potassium (6.85) (Cmol/kg), phosphorus (697.4 mg/kg); C/N (60.33)	Not applicable	Karanja et al. [38]
Donkey manure	Magnesium (13.85), calcium (30.38), potassium (8.81) (Cmol/kg), C/N (52.0)		
Chicken manure	Magnesium (17.99), calcium (39.41), potassium (10.0) (Cmol/kg); C/N 58.67		
Rice straw	Total carbon (40.1), total nitrogen (0.66), TS (93.5), VS (87.5), MC (6.5), ash content (12.5) [%], C/N (60.4)	Bacteriodetes, Firmicutes, Proteobacteria, Spirochaeta, Euryarchaeota, Chloroflexi, Clostridiales, Anaerolineaceae, Christensenellaceae, Rhodobacteriaceae, Peptostreptococcacea, Spirochaetaceae, *Ruminococcus*, *Proteiniphilium*, *Methanobacterium*, *Methanosarcina*, *methanobrevibacter*, *Clostridium*	Zealand et al. [39]
Dairy manure	Total carbon (40.9), total nitrogen (4.09), TS (11.1), VS (84.5), MC (89.7), ash content (25.7) [%], C/N (10.0)		
Maize silage	pH (3.77), TS (37), VS (96) [%]; chemical oxygen demand (38.9), volatile fatty acids (1.05) [g/L]	Draconibacteriaceae, Rikenellaceae, Anaerolinaceae, Ruminococcaceae, Pseudomonadaceae, Carnobacteriaceae, Porphyromonadaceae, Campylobacteriaceae, moraxellaceae, Lacnospiraceae, Aeromonadaceae, Bacteriodaceae, Acidaminicoccaceae, Prevotellaceae, Desulfovibrionaceae, Spirochaetaceae, Synergistaceae, Xanthomonadaceae, Christensenellaceae, Methanoplasmatales, methanosaetaceae, Methanobacteriaceaea, thermoplasmatales, Methanomicrobiaceae	Wojcieszak et al. [40]
Cattle slurry	pH (7.45), TS (2.21), VS (45.60) [%]; chemical oxygen demand (18.40), volatile fatty acids (11.30) (g/L)		
Ensiled maize stalk	Dry matter (301), organic dry matter (276.6), ash content (24.7) [g/kg], neutral detergent fibre (65.45), acid detergent fibre (3.69), Crude protein (5.35) [%DM]	Firmicutes, Chloroflexi, Bacteriodetes, Proteobacteria, Synergistes, Actinobacteria, artribacteria, Clostridia, Anaerolinea, Bacteriodia, Carnobacteriaceae, Moraxellaceae, Lachnospiraceae, Porphyromonadaceae, Corynebacteriaceae, Anaerolinaceae, Ruminococcaceae, Streptococcaceae, Rikenellaceae, *Trichococcus*	Zhang et al. [41]

TS, total solids; VS, volatile solids; MC, moisture content; DM, dry matter; C/N, carbon to nitrogen ratio.

**Table 2:** Differentiating psychrophilic, mesophilic, and thermophilic AD based on different process parameters [23,53,73,136,137,138,139,140,141].

Process Parameters	Categories of Anaerobic Digestion Based on Temperature		
	Psychrophilic AD	Mesophilic AD	Thermophilic AD
Temperature range	15–25 °C	35–39 °C	50–56 °C
Pathogen inactivation	Between 1 and 2-log removal of *E. coli*, *Salmonella* spp., *Yersinia* enterocolitica, *Campylobacter* spp., and Listeria monocytogenes cells by a co-digesting mixture of pinewood sawdust and pig manure treated over a temperature of 13.16–24.56.	>2 log removal of the indicator bacterium, *E. coli*, during the anaerobic mesophilic treatment of black water in upflow anaerobic sludge blanket (UASB) reactors at 35 °C.	Temperature is the most important parameter, based on bacterial inactivation, and thermophilic conditions led to the highest reductions in pathogens. For example, 4–5 log removal of the *E. coli* indicator bacterium during the anaerobic thermophilic treatment (55–60 °C) of black water, resulting in complete elimination of the indicator bacterium in the effluent discharged from upflow anaerobic sludge blanket (UASB) reactors at 35 °C.
Process stability	A critical parameter in the performance and stability of AD animal manure is ammonia nitrogen. Extended solids/hydraulic retention times in psychrophilic AD in sequencing batch reactors (PADSBR) enhanced biomass acclimation even at high ammonia. So VFA, an indicator of process stability, did not accumulate in PADSBR.	During the anaerobic co-digestion of two-phase olive-mill waste and cattle manure, the accumulation of propionic acid was the process control parameter, causing imbalance. To achieve a stable performance, a total of 140 days was required, but the start phase was operated in a batch mode for 97 days, reaching a final propionic acid of 12.77 mg/L. Subsequently, a semi-continuous mode was applied with an HRT of 40 days, yielding methane productivity of 0.34 LCH_4_/L_R_d.	The key stability factor is the ratio of acidity (VFAs) versus alkalinity. The accumulation of acetic acid in the system co-digesting sun-dried sugar beet pulp and cow manure at thermophilic temperature was the main cause of instability in the system at HRT. Nevertheless, the best global system performance was achieved at an HRT as short as 5 days (OLR of 12.47 gVS/L_reactor_∙d) with a biogas yield of 315 mL/gVS_added_.
Methane yield	The solids retention time of psychrophilic AD is twice to thrice compared to mesophilic AD because of a lower rate of hydrolysis and a decrease in the population, growth, and activity of microbial consortia. Nonetheless, producing methane under such low temperatures is possible via the application of a high inoculation rate. The psychropilic anaerobic digestion of cow manure and food waste together with cold-adapted inoculum resulted in cumulative specific methane yields of 0.874 ± 0.231 and 0.552 ± 0.089 L CH_4_ g^−1^ volatile solids, respectively, after 14 weeks. In addition, the absence of a cold-adapted inoculum led to acidification and no methane production during the process.	The methane production of four animal wastes, including carnivore, herbivore and omnivore, was investigated at both thermophilic and mesophilic anaerobic digestion. Accordingly, the methane yield recovered at thermophilic treatment was higher than the mesophilic ones. For example, 0.326 L/g VS and 0.391 L/g VS methane yields realised from lion manure under the mesophilic and thermophilic AD, respectively, were higher than those of herbivore and omnivore waste.	This process could accelerate biochemical reactions, leading to higher efficiency of degradation together with higher methane production rates. It also involves improved kinetics, economic and environmental sustainability. Cumulative volumes of biogas yields were registered: 4.78 L for 1 L of the bioreactor working volume with substrate loading 30 g/L of wheat straw, 7.39 L for 40 g/L, and 8.22 L for 45 g/L. The degree of biodegradation was calculated to be 68.9%, 74% and 72%. The biomethane content of biogas was 60%.

**Table 3:** Summary of factors affecting anaerobic digestion.

Parameters	Description, Optimal Ranges, and Impact on Biogas Yield	References
Organic loading rate (OLR)	OLR determines the quantity of volatile solids that are to be fed into the digester per unit time (day). Therefore, OLR relies on the nature of the substrate employed as input into the digester. For a specific lignocellulosic biomass (LCB), the typical range is from 1 to 6 kg of volatile solids (VS) per cubic metre per day (kg VS/m^3^/day). It is equally affected by other factors such as temperature, digestion type, and the stirring or mixing rate. The OLR is indirectly related to HR. Process stability is the driving factor behind the successful operation of a digester treating food waste. Gradually, increasing the OLR leads to increasing biogas yields, but excessive organic loading rates subject an operating digester to instabilities, improving process efficiency, but affecting the balance of metabolic activities within the distinct microbial groups. The lack of balance at a single degradation step will disrupt the entire process, causing a decrease in both biogas and methane yields. This is because a balanced interaction between microbes in the bio-digestion chain is central to stable and efficient gas production. For example, a temperature of 45 °C and OLR of 6.99 kg VS/m^3^·day were observed as the optimum conditions for the anaerobic digestion of municipal solid wastes at Alepo, enhancing biogas production.	[175,176,177,178]
Hydraulic retention time (HRT)	It denotes the duration needed for the complete degradation of the organic wastes within the biodigester. In other words, it is the mean residence time of the substrate in the system or the average process time of the influent in the reactor, and, theoretically, it is equivalent to the ratio of the digester volume to the daily intake flow rate. HRT is regarded as one of the most significant parameters affecting the performance of AD. However, changes in HRT may also affect the microbiome community structure and thus the microbial balance within the digester. Reducing the HRT leads to an increase in methane production and thus economic efficiency. However, changes in HRT may also affect the microbiome community structure and thus the microbial balance within the digester. HRT represents the available duration for microbial growth and for bioconversion of the wastes to biogas. Primarily, it relies on the type of substrate, in terms of its concentration and microbial concentration, and the operational conditions, including temperature, process stability (pH), and organic loading rate. Depending on the substrate, longer HRT is needed for lignocellulosic biomass, allowing ample time for optimal breakdown of the complex compounds because of their persistence with anaerobic microbes, in addition to preventing the washout of slowly growing microbes. The longer the HRT, the higher the biogas yields. However, the shorter HRT is desirable as it is associated with reduced capital cost and an increase in the efficiency of the process. For a single-stage anaerobic co-digestion of agricultural residues, 20 days was required, but depending on the substrate and temperature of operation, HRT can occur in the range 10–60 days.	[179,180,181,182,183,184]
Carbon to nitrogen ratio (C/N)	It is part of the substrate characteristics, which can be described in relation to macromolecule or polymer compositions, including carbohydrates, lipids, and proteins. Unbalanced nutrients are a limiting factor for the AD of organic waste, but can be addressed via the co-digestion of organic mixtures to augment nutrition and C/N ratios. Proper composition of feedstock in terms of carbon and nitrogen is vital for a balanced C/N ratio that is needed for an efficient AD process. Both elements, C and N, are necessary for the growth of anaerobic microorganisms in that C is utilised as a source of energy while N is employed in the synthesis of proteins, amino acids, and building cell structures. The optimal range of C/N needed by microbes during AD is dependent on the substrate, but the ratio of 20–30:1 is endorsed. A low C/N ratio leads to ammonia accumulation, inhibiting methanogenesis, while a high C/N ratio leads to slower decomposition rates due to nitrogen limitation. The right balance of C and N in the substrate guarantees stable and efficient biogas production owing to balanced microbial activity. The ratio of C to N is therefore used to optimise the process of co-digestion. The co-digestion of cabbage and cauliflower leaves and stalks (CCF) together with food waste at a C/N ratio of 45 added value to the agricultural wastes, yielding a high biodegradability (98%), a methane yield of 475 mL_STP_ CH_4_/g VS, and an organic loading rate (OLR) of 0.06 kg of VS/m^3^ h for the CCF and FW mixture (CCF + FW).	[58,185,186,187,188]
Ph (pH)	To ensure the stability of the digester, pH is the key parameter to be considered and monitored. The performance of the AD process is greatly impacted by the pH; therefore, it is a fundamental parameter for the growth of different microbes at various stages. The optimal range is 6.8–7.4. The degradation of substrates at each stage of the process leads to different metabolites affecting the pH (volatile fatty acids) and alkalinity of the medium under decomposition. Overall, the pH exerts an effect on the chemical equilibria of ammonia, hydrogen sulphide, and VFAs, which could inhibit microbial activity, thus negatively impacting biogas production. The pH can drop meaningfully to less than 3 if the VFAs generated during acidogenesis are not metabolised, thus leading to process failure. A low pH demonstrates an inherent inhibitory effect on the entire process, while a higher pH causes partial chemical breakdown of the complex organic matter with a potential for faster microbial conversion. It offers advantages with respect to CO_2_ capture and the direct production of methane gas. Small variations in pH of approximately 0.5 can greatly impact microbial metabolism and reaction kinetics, affecting gas production. Thus, methanogens are particularly sensitive to alterations in pH and function best within a pH close to 7.	[90,186,188,189,190]

**Table 5:** Inactivation of bacterial pathogens found in animal manure through anaerobic digestion.

Bacterial Pathogens	Substrate Types/Biodigester Type	Rate and Duration of Reduction (Log)	Anaerobic Digestion Types and Retention Periods	Methods of Assessing Pathogen Survival	Countries	References
*Escherichia coli* 0157	Food waste, bovine slurry grease trap waste/Ramboldi tubes (100 mL) that were batch operated	1 log reduction from 1.6 to 2.8 days	Co-digestion at 37 °C (mesophilic temperature) for 10 days	Enumeration by cultivation and confirmation of isolates via polymerase chain reaction	Ireland	Russell et al. [314].
*Listeria monocytogenes*		1 log reduction from 3.1 to 23.5 days				
*Enterococcus faecalis*		1 log reduction from 2.2 to 6.6 days				
*Clostridium sporogenes*		1 log reduction from 2.4 to 9.1 days				
*Salmonella Newport*		1 log reduction from 1.5 to 2.8 days				
*Escherichia coli*	Dairy manure fermented using balloon type biodigester under batch operation	1 log reduction for 62 days	Mono-digestion at ambient temperature for 6 months	Culture-based method (total viable plate method)	South Africa (Eastern Cape province)	Manyi-Loh et al. [42].
*Salmonella* species		1 to 2 log reduction for 133 days				
*Campylobacter* species		1 log reduction for 18 days				
*Vibrio*	Brewers spent grains, palm oil mill effluent, and livestock manure (cow dung, swine slurry, and poultry droppings). 100 mL amber borosilicate serum bottles, which were batch operated	2.3 log reduction	Co-digestion at 40 °C for 30 days	Standard plate count method	Nigeria	Ndubuisi-Nnaji et al. [179].
*Salmonella* sp.		0.20 log reduction				
*Staphylococcus*		0.27 log reduction				
*Escherichia coli*	Pig manure and pine wood sawdust were digested using a 75 mL capacity single-stage steel biodigester, which was batch operated	1 log reduction for 77 days	Co-digestion at a psychrophilic temperature range for seven (7) months	Standard plate count method	South Africa (Eastern Cape Province)	Manyi-Loh et al. [204].
*Salmonella* species		1–2 log reduction for 84 days				
*Yersinia enterocolitica*		1 log reduction for 98 days				
*Campylobacter* species		1 log reduction for 112 days				
*Listeria monocytogenes*		1 log reduction for 175 days				
Total coliforms	Pig manure fermented with maize silage in agricultural biogas plants, which were operated under a continuous mode	2.3 log reductions	Co-digestion at mesophilic temperature regime (30–42 °C)	Most probable number method	Poland	Grudziński et al. [253].
Enterococci		Below detection limits				

**Table 6:** Logarithmic removal of antibiotic resistance genes through anaerobic digestion of different biowaste, including animal manure.

Investigations	Antibiotic Resistance Genes Involved	Methods for Detecting ARGs	Inactivation/ Reductions	Countries	References
50 samples of sewage sludge extracted from two wastewater plants.	(bla_OXA_, bla_TEM_, *erm*B, *qnr*B, *tet* (A)-(W), *sulI*-II).	Antibiotic susceptibility testing and quantitative polymerase chain reaction.	1 log	Northern Italy	Franchitti et al. [334].
31 sludge samples from the organic fraction of municipal solid waste treatment plants.	*bla*_TEM_, *bla*_OXA_, *erm*B, *qnr*B, *sul*I, *sul*II, *tetA*, and *tetW.*	Quantitative polymerase chain reaction.	<1 log	Northern Italy	Franchitti et al. [334].
Swine wastewater, including urine, residual faeces, and flushing water.	*erm*B, *tet*X, *mef*A, *erm*f, *sul*2, *tet*M.		0.21–1.34 logs	North and South China	Sui et al. [341].
Anaerobic co-digestion of food waste and pig manure together with dewatered sludge (inoculum).	A total of 199 ARGs and 12 mobile genetic elements clustered based on antibiotics such as tetracycline, vancomycins, sulfonamides, beta-lactams, aminoglycosides, macrolides, lincosamide, streptogramin, etc.	High-throughput quantitative polymerase chain reaction.	1.24 logs	Ireland	Wang et al. [342]
Dairy manure mixed with granules-inocula subjected to thermophilic anaerobic digestion.	16S rRNA, *tnp*A (mobile genetic element), *intl*1(class 1 integrase), *sul*II.	Real-time quantitative polymerase chain reaction.	0.6–1.3 log	California, USA	Wang et al. [196].
Dairy cow manure subjected to anaerobic mono-digestion at mesophilic temperatures.	*bla*_TEM_, *tet*A, and *tet*B.	Quantitative polymerase chain reaction.	3-log	Hokkaido, Japan	Katada et al. [343].
Zootechnical wastes subjected to anaerobic mono-digestion at mesophilic temperatures.	*sul*1, *sul*2, *qnr*S, *qep*A, aac-(6′)-Ib-cr, *intl*1	Real-time quantitative polymerase chain reaction.	0–1.08-log	Italy	Visca et al. [344].
Cattle manure treated under anaerobic digestion operated at mesophilic temperature.	*intI*1, *sul*1, and *tet*(A)	Real-time quantitative polymerase chain reaction.	≥0.3-log	Southern-eastern Wisconsin, USA	Burch et al. [345].
Mixed raw sludge digester anaerobically using a bench-scale continuously stirred anaerobic at thermophilic temperatures.	*aad*A, bla_OXA1_-bla_OXA30_, *str*B, *sul*I, *int*I, *cml*A, *erm*B, *tet*M.	Quantitative polymerase chain reaction.	≥1.5-log	Prague, Czech Republic	Budatala et al. [49].
Waste-activated sludge from a wastewater treatment plant (WWTP) subjected to anaerobic digestion performed under two temperature conditions: (i) Mesophilic temperature, 35 °C, (ii) Thermophilic temperature, 55 °C	Extracellular ARGs (eARGs): *sul*I, *sul*II, *tet*A, *tet*O, *tet*X, *bla*_TEM_, *bla*_SHV_.	Quantitative polymerase chain reaction.	0.1–1.2 Log 0.33–1.5-log	Beijing, China	Zou et al. [346].
Sludge procured from a full-scale WWTP employed in temperature-phased anaerobic digestion [i.e., sequential thermophilic (55 °C) and mesophilic (37 °C) anaerobic digestion].	aac(6′)-Ib-cr, *bla*_TEM_, *drf*A, *sul*I, *sul*2, *erm*B, *mef*A, *tet*A, *tet*B and *tet*X.	Real-time quantitative polymerase chain reaction.	0–0.9-log	Australia	Liu et al. [347].
Mixed sludge from two WWTP subjected to thermal hydrolysis prior to mesophilic anaerobic digestion.	*tet*M, *bla-_IMP_*, *bla-_CTX-M_*, *qnr*S, *aac*(3)-1, *dfr*A1, *dfr*A5, *dfr*A7, *dfr*A12, *erm*F, *sul*I, *intl*1, 16S rRNA.	Single-gen quantitative polymerase chain reaction and high-throughput quantitative polymerase chain reaction.	4–4.8 log	United Kingdom	Redhead et al. [348].
Fresh cattle manure subjected to anaerobic digestion conducted at ambient temperature during winter and summer periods.	aphA2, ermB, blaTEM-1.	Quantitative polymerase chain reaction.	0.72–1-log (winter) 1.2–2.2-log (summer)	Minas Gerais State, Brazil	Resende et al. [349].
Waste-activated sludge for Urban WWTP exposed to anaerobic digestion at mesophilic and thermophilic temperatures following pH and oxidising agent pretreatments.	*tet*A, *tet*C, *tet*Q, tetX, *erm*B, *sul*I, *sul*2, *sul*3, *aad*A-1, *intl*1.	Qualitative polymerase chain reaction and high-throughput real-time fluorescence quantification polymerase chain reaction.	0.6-log	Baoding, China	Wang et al. [340].

ARG, antibiotic resistance genes; *erm*, erythromycin resistance gene; *sul*1, sulfonamide resistance genes; *tet*, tetracycline resistance gene; bla_TEM_, *bla*_SHV_ & *bla*_OXA_, beta-lactam resistance genes; *drf* A, dihydrofolate reductase; *cml*, chloramphenicol resistance gene; *str*A, streptomycin resistance gene; *aad*, aminoglycoside adenyl transferase; *qnr*S, plasmid-mediated quinolone resistance gene; *qep*A, plasmid-mediated efflux pump; *tnp*A, transposase gene; *aac-(6′)-Ib-cr*, plasmid-mediated quinolone resistance; *mef*A, macrolide efflux gene; *aph*, aminoglycoside phosphotransferase.

## Data Availability

No new data were created or analyzed in this study. Data sharing is not applicable.
